# Guidelines for Optimizing Type S Nonribosomal Peptide Synthetases

**DOI:** 10.1021/acssynbio.3c00295

**Published:** 2023-07-31

**Authors:** Nadya Abbood, Juliana Effert, Kenan A. J. Bozhueyuek, Helge B. Bode

**Affiliations:** † Max-Planck-Institute for Terrestrial Microbiology, Department of Natural Products in Organismic Interactions, 35043 Marburg, Germany; ‡ Molecular Biotechnology, Institute of Molecular Biosciences, Goethe University Frankfurt, 60438 Frankfurt am Main, Germany; § Myria Biosciences AG, Mattenstrasse 26, 4058 Basel, Switzerland; ∥ Chemical Biology, Department of Chemistry, Philipps-University Marburg, 35043 Marburg, Germany; ⊥ Senckenberg Gesellschaft für Naturforschung, 60325 Frankfurt am Main, Germany; # Center for Synthetic Microbiology (SYNMIKRO), Phillips University Marburg, 35043 Marburg, Germany

**Keywords:** synthetic biology, natural products, NRPS engineering, nonribosomal peptides, biocombinatorial approach, iterative optimization

## Abstract

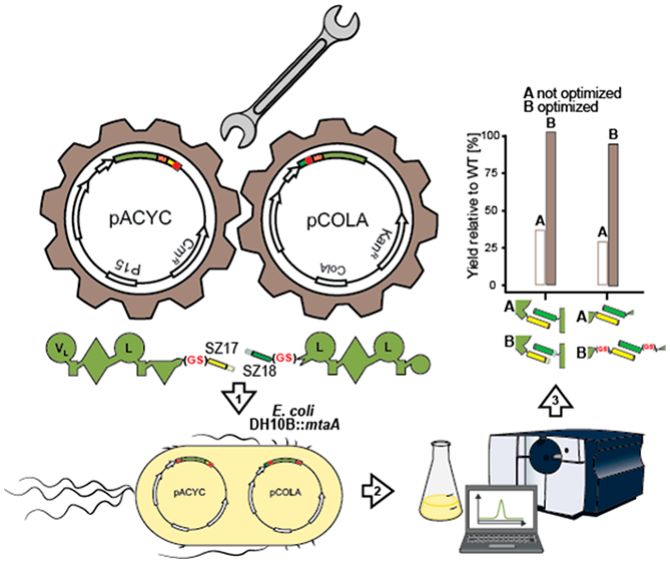

Bacterial biosynthetic assembly lines, such as nonribosomal peptide synthetases (NRPSs) and polyketide synthases (PKSs), play a crucial role in the synthesis of natural products that have significant therapeutic potential. The ability to engineer these biosynthetic assembly lines offers opportunities to produce artificial nonribosomal peptides, polyketides, and their hybrids with improved properties. In this study, we introduced a synthetic NRPS variant, termed type S NRPS, which simplifies the engineering process and enables biocombinatorial approaches for generating nonribosomal peptide libraries in a parallelized high-throughput manner. However, initial generations of type S NRPSs exhibited a bottleneck that led to significantly reduced production yields. To address this challenge, we employed two optimization strategies. First, we truncated SYNZIPs from the N- and/or C-terminus of the NRPS. SYNZIPs comprise a large set of well-characterized synthetic protein interaction reagents. Second, we incorporated a structurally flexible glycine–serine linker between the NRPS protein and the attached SYNZIP, aiming to improve dynamic domain–domain interactions. Through an iterative optimization process, we achieved remarkable improvements in production yields, with titer increases of up to 55-fold compared to the nonoptimized counterparts. These optimizations successfully restored production levels of type S NRPSs to those observed in wild-type NRPSs and even surpassed them. Overall, our findings demonstrate the potential of engineering bacterial biosynthetic assembly lines for the production of artificial nonribosomal peptides. In addition, optimizing the SYNZIP toolbox can have valuable implications for diverse applications in synthetic biology, such as metabolic engineering, cell signaling studies, or engineering of other multienzyme complexes, such as PKSs.

## Introduction

Multimodular enzyme complexes, such as polyketide synthases (PKSs) and nonribosomal peptide synthetases (NRPSs), are often the subject of synthetic biology (SynBio)^1,2^—because they produce a variety of valuable chemicals or pharmaceutically relevant peptides. Engineering these biosynthetic assembly lines can produce artificial polyketides, nonribosomal peptides (NRPs), or hybrids thereof with new or improved properties.^3^

Recently, we have focused in particular on developing more efficient SynBio methods for engineering modular NRPSs (type I NRPSs),^4,5^ which have been in their infancy for decades.^6,7^ By introducing the exchange unit (XU) and the XU condensation domain (XUC) concepts, we provided the necessary means to enable more reproducible, predictable, and robust engineering than before.^4,5^ The XU concept, for example, leverages a fusion point located between the NRPS’s condensation (C) and adenylation (A) domains within the structurally flexible region of the C–A interdomain linker.^4^ A-domains in NRPSs are responsible for ATP-dependent selection and activation of specific amino acids (AAs), while C-domains catalyze peptide-bond formation between two AA residues. Together with the T domain, which transports the activated AA from the A-domain to the C-domain, they form the core domains of a canonical minimal NRPS module (C–A–T).^8^ NRPSs usually consist of not just one but several sequentially repeating modules, each responsible for the incorporation and optional functional group modification of a specific AA. Commonly, the number of modules in an NRPS corresponds directly to the number of AAs incorporated into the associated NRP.^8−10^ Iterative systems, such as the enniatin^11^ or rhabdopeptide^12^ producing NRPSs, however, deviate from this collinearity rule and reuse individual modules.

Despite recent advancements such as the successful *in vivo* engineering of complex NRPS systems using CRISPR-Cas9 technology^13^ and the modification of NRPS through evolutionary-inspired A subdomain swaps,^14^ the process of assembly line engineering still poses challenges due to the large size and repetitive nature of these systems.^7^ These factors make the engineering process difficult and time-consuming. Traditional NRPS cloning and engineering often require elaborated cloning strategies such as yeast cloning, LLHR^15^ (linear plus linear homologous recombination-mediated recombineering), or ExoCET^16^ (exonuclease combined with RecET recombination), which are frequently accompanied by technical limitations.^7^ Therefore, we established a new synthetic type of NRPS (type S) that allows easier and faster cloning by splitting large single-protein multimodular NRPSs into two or three smaller and independently expressible subunits that are reconstituted to full length in the living cell via the interaction of high-affinity leucine zippers (SYNZIPs).^17,18^ Further studies have recently used the same or similar high-affinity tags, e.g., SpyTag/SpyCatcher,^19^ zinc fingers,^20^ and SYNZIPs,^21^ to mediate protein–protein interaction of split NRPSs or PKSs. Introduction of these protein tags has increased productions titers (in vanlinomycin synthesis),^19^ mediated DNA–protein recognition (of DNA-templated NRPS),^20^ or enabled generation of chimeric PKS (of 6-deoxyerythronolide B synthase),^21^ emphasizing the diverse applicability of the various interaction toolboxes for SynBio applications.

**Figure 1:**
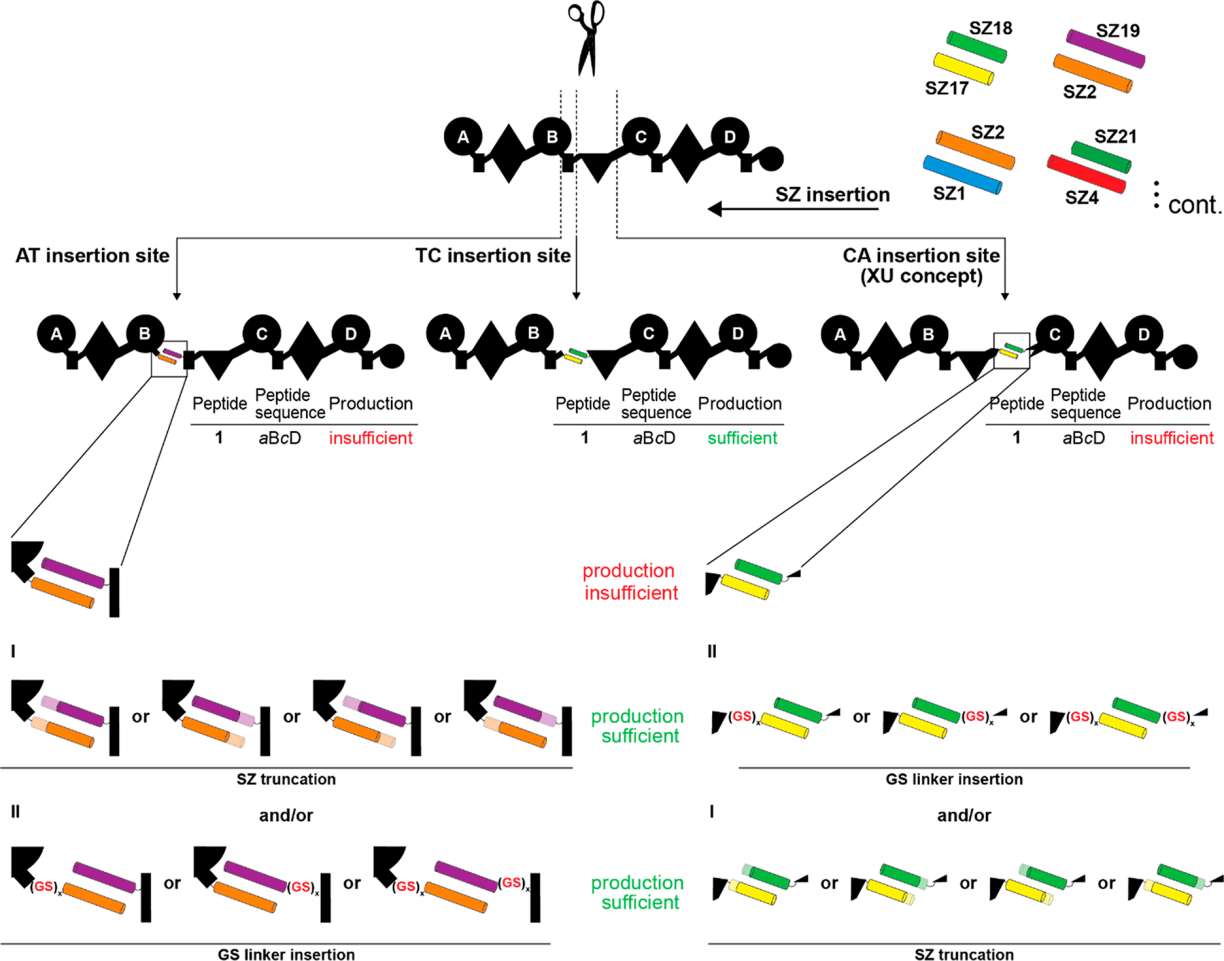
Optimization of type S NRPSs. From a pool of 27 biophysically characterized pairs,^25^ SYNZIPs (SZ) can be introduced in three different positions within the A–T, T–C, and C–A linker regions, respectively. For type S NRPSs yielding insufficient production titers, we explored two optimization strategies: (I) the truncation of SYNZIPs either from the C-terminal, N-terminal, or both sides; (II) the insertion of flexible GS linkers upstream of the SYNZIP donor, downstream of the SYNZIP acceptor, or into both positions. For domain assignment, the following symbols are used: (A, large circles), (T, rectangle), (C, triangle), (C/E, diamond), (TE, small circle); imaginary substrate specificities are assigned for all A-domains and indicated by capital letters.

The ability to separate NRPS-encoding biosynthetic gene clusters into smaller DNA fragments that encode partial NRPS proteins (subunits) and then distribute the respective gene fragments onto different expression plasmids naturally simplifies cloning – making “standard” *in vitro* cloning strategies such as Gibson,^22^ HiFi, and Hot Fusion^23^ (Isothermal-) assembly sufficient. To reconstitute the communication of generated NRPS protein subunits, we attached SYNZIPs that post-translationally restore the full-length biosynthetic capacity of the modular NRPS system in the living cell.^17,18,24,25^ However, type S NRPSs not only simplify NRPS engineering but also offer, for the first time, the possibility of true biocombinatorial approaches to the design of natural product-like NRP libraries.^17^ Type S NRPSs can thus be created much faster and in unprecedented numbers compared to conventional bioengineering approaches (Figure S1). Previously, we demonstrated the vast biocombinatorial potential of type S NRPSs by creating bi- and tripartite NRPS libraries, wherein each library member consists of two or three type S subunits, respectively, which are post-translationally assembled to full length *in vivo* via the interaction of SYNZIPs, biosynthesizing about 50 NRPs, NRP derivatives, and new to nature artificial NRPs.^17^ In this study, compared to our original proof of concept study on type S NRPSs, wherein we functionally introduced SYNZIPs within C–A linker regions at the previously defined splicing position in between individual XUs (A–T–C tridomain units), we further broadened the applicability of SYNZIPs by successfully introducing them in between A–T and T–C interdomain linker regions.^17^ However, one bottleneck, particularly for type S NRPSs with attached SYNZIPs within the C–A linker region and type S NRPSs with certain SYNZIPs pairs (i.e., SZ1:2 in tripartite type S NRPSs), was the significant drop in production yield observed.^17,18^ Herein, we will describe the iterative optimization process (Figure 1) of SYNZIPs and type S assembly lines, respectively, yielding up to >50-fold increased titers compared to their nonoptimized equivalents. To achieve the desired optimization of NRP biosynthesis, we applied two strategies: (I) truncation of SYNZIPs from the N- and/or C-terminus (Figure 1, I); and (II) introduction of structurally flexible GS (glycine–serine) linkers into type S subunits—in between the NRPS protein subunit and the attached SYNZIP(s) (Figure 1, II).

## Results and Discussion

### Insertion of Glycine–Serine Linker Sequences

**Figure 2:**
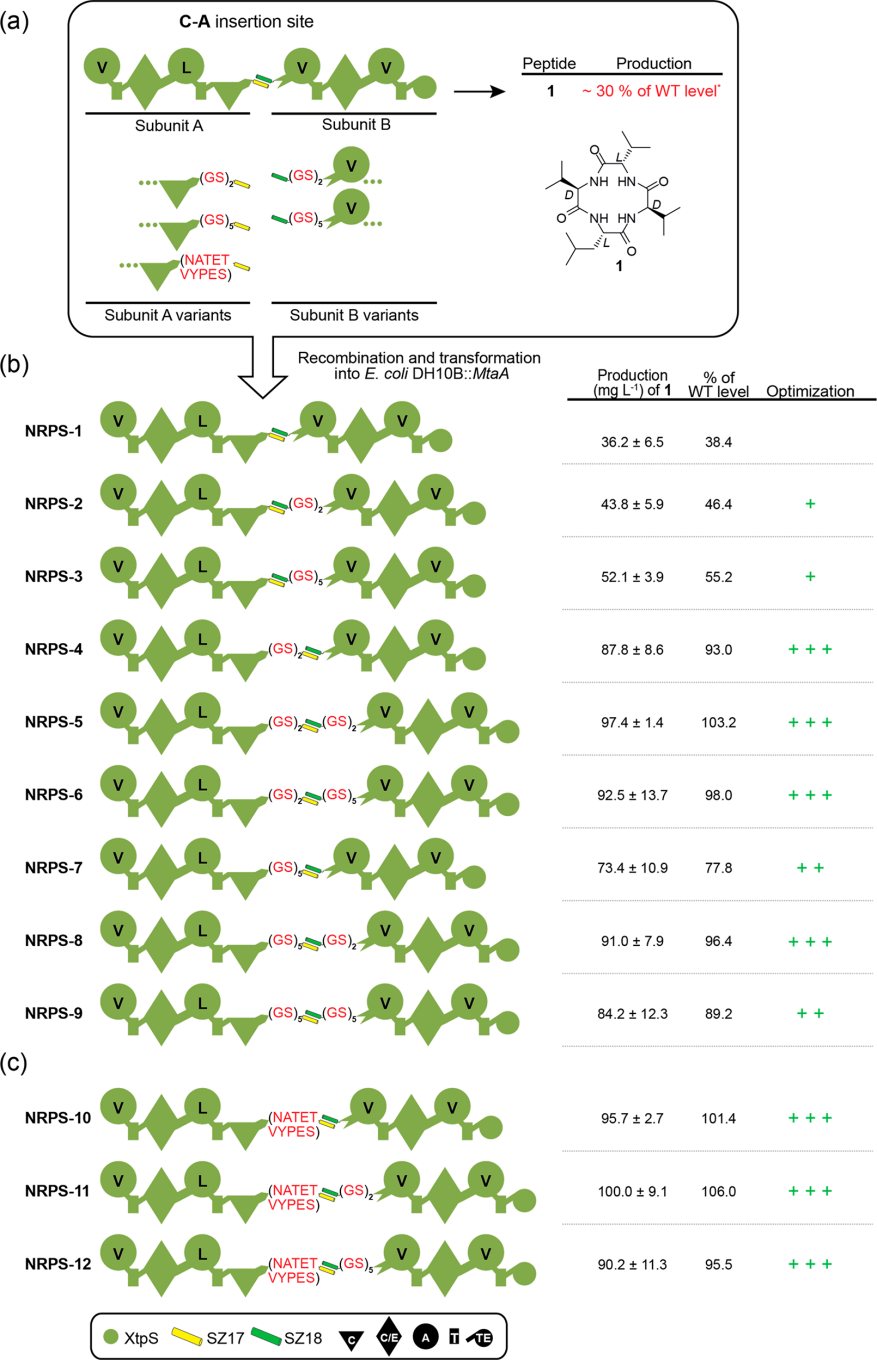
Insertion of synthetic GS stretches of varying length into a type S XtpS system. (a) On average, the production of nonoptimized NRPS-1 is at ∼30% of WT level having a full-length XtpS. A set of modified subunit A and B variants were constructed by inserting GS stretches of 4–10 AAs between the C-terminus of XtpS subunit A and SZ17 and SZ18 and the *N*-terminus of subunit B. (b) Generated modified subunits were recombined with nonmodified subunits and transformed into *E. coli* DH10B::*mtaA* to obtain NRPS-2 to -9. (c) The native 10 AAs (NATETVYPES) were additionally inserted between the C-terminus of subunit A and SZ17. Production titers of NRPS-1 to -12 were compared with each other and rated + , ++, and +++. Corresponding peptide yields (mg L^–1^) and standard deviations are obtained from biological triplicate experiments. For domain assignment, the following symbols are used: (A, large circles), (T, rectangle), (C, triangle), (C/E, diamond), and (TE, small circle); substrate specificities are assigned for all A-domains and indicated by capital letters.

Previously, we introduced the antiparallel SYNZIP pair 17 and 18 (SZ17:18) at three different positions within the A–T, T–C, and C–A linker regions (the exact introduction sites are highlighted in Figure S2) of the xenotetrapeptide-producing synthetase (XtpS) from *Xenorhabdus nematophila* ATCC 19061,^26^ to create three different two protein type S XtpS variants.^17^ Although all variants showed catalytic activity, synthesizing the expected cyclic xenotetrapeptide (**1**, cyclo(*v*L*v*V); D-AA with small letters and in italics throughout the paper), we also found that the type S XtpS split within the C–A linker region (cf., NRPS-1, Figure 2), resulting in the lowest production of **1** with ∼30% compared to the WT level (Figure 2a). In contrast, both type S XtpS splits within the A–T and T–C linker regions produced **1** at ∼86% compared to WT XtpS level (Figure S2).^26^ Of note, throughout the present work, mentioned recombinant type S and WT NRPSs were produced heterologously in *E. coli* DH10B::*mtaA*.^27^*MtaA* encodes a phosphopantetheinyl transferase (PPtase) with a broad substrate specificity from *Stigmatella aurantiaca*, which is required to convert the T domain to its active holo form.^28^ The resulting peptides (Table S1) and yields were confirmed by HPLC-MS/MS and comparison of retention times with synthetic standards (Supporting Information Figures S3–24).

In our endeavor to further optimize type S assembly lines, we concluded from these initial results, along with available structural data^29^ of the C–A-domain–domain interface and linker regions, that the introduction of SYNZIPs, due to their sheer size and rigidity, could hinder the necessary structural rearrangements and thus catalytically ideal positioning of involved C, A, and T domains during the NRPS catalytic cycle. We hypothesized that more spatial flexibility would enhance dynamic domain–domain interactions of the type S XtpS (NRPS-1) variant. Hence, we introduced synthetic glycine–serine (GS) segments of 4 and 10 AAs in length between the C-terminus of NRPS-1 subunit A (subA_GS0) and SZ17 (subA_GS2, subA_GS5) as well as SZ18 and the N-terminus of NRPS-1 subunit B (sub_GS0, sub_GS2, and sub_GS5), respectively (Figure 2a).

To test these modifications, we cotransformed, produced, and analyzed all possible type S subA:subB combinations (NRPS-2 to -9) and compared peptide yields of **1** with the previously created unmodified type S NRPS-1 (subA_GS0:subB_GS0) and WT XtpS. In brief, all modified type S NRPSs (NRPS-2 to -9) showed increased catalytic activity compared to NRPS-1 (38 mg L^–1^), producing **1** at titers of 44–100 mg L^–1^. Interestingly, while insertions of GS2 (NRPS-2; 46 mg L^–1^) and GS5 (NRPS-3; 55 mg L^–1^) stretches downstream of SZ18 (subB_GS2 and sub_GS5), respectively, led to the least increase in peptide production, any modification upstream of SZ17 (subA_GS2, subA_GS5) significantly raised production titers of **1** close to (NRPS-7; 78 mg L^–1^) or back to (NRPS-4; 88 mg L^–1^) WT level.

Additionally, to run a control experiment, we reinserted the original 10 AAs (NATETVYPES) of the C–A linker that we removed in our original feasibility study^17^ to maintain the native spacing of the C- and A-domains involved. As we identified the position upstream of SZ17 as structurally more suitable, we chose this position to reinsert the original AAs into NRPS-1 subunit A (subA_NATETVYPES), shifting the original XU fusion site by 10 AAs (Figure S2). From the three additionally generated synthetases harboring subA_NATETVYPES (NRPS-10 to -12, Figure 2c), NRPS-11 was the best-producing synthetase resulting from a combination of subA_NATETVYPES with subB_GS2 (100 mg L^–1^), biosynthesizing **1** at 106% compared to WT XtpS and 278% compared to NRPS-1.

**Figure 3:**
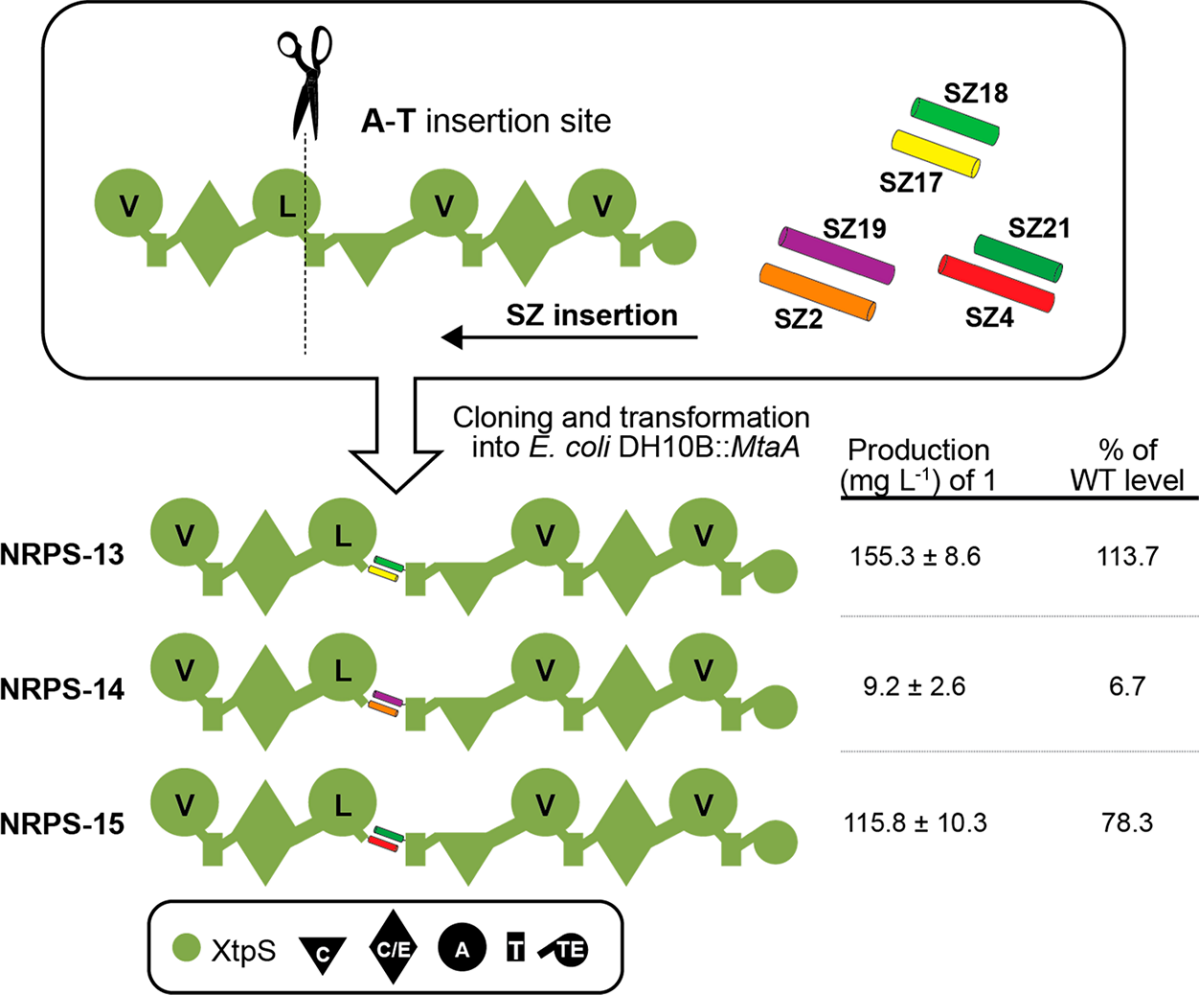
Introduction of other SYNZIP pairs into XtpS. Two parallel interacting SYNZIPs, SZ2:19 and SZ4:21, were introduced into the A–T position of module two to generate NRPS-14 and NRPS-15. Corresponding peptide yields (milligrams per liter) and standard deviations are obtained from biological triplicate experiments. Domain assignment is as described before. For domain assignment, the following symbols are used: (A, large circles), (T, rectangle), (C, triangle), (C/E, diamond), (TE, small circle); substrate specificities are assigned for all A-domains and indicated by capital letters.

**Figure 4:**
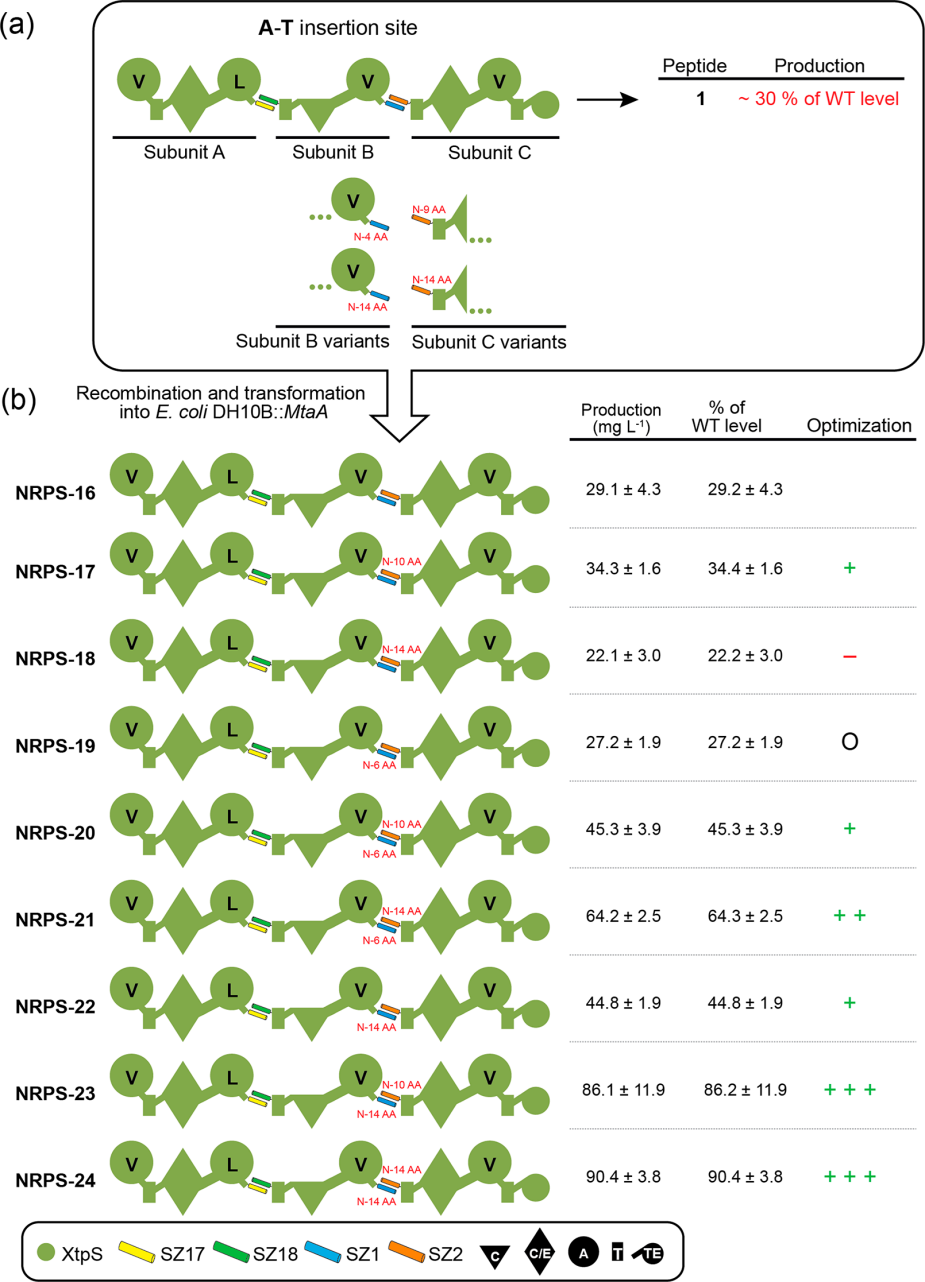
Truncation of SZ1:2 in the tripartite XtpS. The production of nonoptimized NRPS-16 is on average ∼30% of the WT level of a single-protein XtpS variant. Here, WT XtpS produced **1** at titers of 99.9 mg L^–1^. A set of modified subunit B and C variants were constructed by N-terminally truncating SZ1 by 6 and 14 AAs and SZ2 by 10 and 14 AAs. Generated modified subunits were recombined with nonmodified subunits and transformed into *E. coli* DH10B::*mtaA* to obtain NRPS-16 to -24. Corresponding peptide yields (mg L^–1^) and standard deviations are obtained from biological triplicate experiments. Production titers of NRPS-16 to -24 were compared with each other and rated −, O, +, ++, and +++. For domain assignment, the following symbols are used: (A, large circles), (T, rectangle), (C, triangle), (C/E, diamond), (TE, small circle); substrate specificities are assigned for all A-domains and indicated by capital letters.

Further modified type S XtpS with GS2 and GS5 linker insertions are shown in SI Figure S25 (NRPS-49 to -56). Here, GS stretches were introduced into a tripartite type S XtpS system (also depicted in Figure 4, NRPS-16) partitioned in between the A2–T2 and A3–T3 linker regions by introducing SZ17:18 and SZ1:2 pairs, respectively (SI Figure S2, NRPS-16) – with emphasis on optimizing the latter SYNZIP pair facilitating the specific interaction of the tripartite NRPS’s subunits B and C. Interestingly, again, all created type S assembly lines (NRPS-49 to -56) showed catalytic activity with yields ranging from 30 to 52 mg L^–1^, and all but two (NRPS-51 & -49) showed slightly increased amounts of **1** compared to unmodified type S NRPS-16 but still only at 25% compared to its bipartite counterpart (Figure 3, NRPS-13). From these insights, we concluded that impairments caused by introducing SZ1:2 cannot be overcome by simply introducing flexible GS stretches, making a different optimization strategy necessary, which will be discussed in the following sections.

### Introducing Additional SYNZIP Pairs

Depending on the experimental approach, it might be necessary to introduce additional or other SYNZIP pairs in comparison to the previously applied pairs SZ17:18 and SZ1:2. Based on the observed mixed effects of the previously used SYNZIP pairs on the productivity of type S NRPSs,^17,18^ we decided to evaluate the effects of other SYNZIP pairs on the functionality and productivity of NRPSs. Again targeting XtpS, we introduced two additional parallel interacting SYNZIP pairs, SZ2:19 (NRPS-14) and SZ21:4 (NRPS-15), at the A–T position within module two (Figure 3).

While both NRPS were functional, NRPS-14 (10 mg L^–1^) and -15 (115 mg L^–1^) resulted in reduced biosynthesis of **1** compared to that of WT XtpS (136 mg L^–1^) and NRPS-13 (155 mg L^–1^) (Figure 3), and the impairing effects of SZ2:19 appeared to be significantly greater. Since the experimental setup for NRPS-13 to -15 was identical except for the selected SYNZIP pair, it is obvious that the respective selected SYNZIP pairs are responsible for the observed effects on peptide yields. We, therefore, raised the question: What does the catalytic activity depend on, and which biophysical parameters of SYNZIPs have the most significant influence on the productivity of type S NRPS-13 to -15?

In search of an answer, we took a closer look at the biophysical data of all SYNZIPs used, compiled in a specification sheet provided by Thompson et al.^25^ We found that all three SYNZIP pairs have similar properties, e.g., similar affinities of <10 nm and nontoxicity to the living cell (demonstrated by yeast-two-hybrid experiments with two different reporter genes) but considerably differ in length.^25^ Here, SZ17:18, used in the best-producing NRPS-13, harbors the shortest SYNZIP pair with lengths of 42 AAs (SZ17) and 41 AAs (SZ18), respectively. All other SYNZIPs available and tested so far are significantly longer, ranging from 45 to 54 AAs. Taking into account our previous research,^17,18^ demonstrating that the introduction of SZ1:2, a relatively long SYNZIP pair with 47 (SZ1) and 50 amino acids (SZ2), into tripartite NRPSs substantially reduced production yields, along with the results of GS linker modifications of SZ1:2 (Figure S25), we inferred a direct correlation between the length of SYNZIPs and the productivity of type S NRPSs. Consequently, our next objective was to enhance chimeric type S NRPSs by truncating the utilized SYNZIPs.

### SYNZIP Truncations

We previously demonstrated the possibility of generating tripartite type S assembly lines assembled from three independent type S subunits.^17^ Although type S tripartite NRPSs further enhance the advantages associated with the insertion of SYNZIPs, i.e., increased biocombinatorial potential, they have so far suffered from low production titers (∼30% of WT level, cf., NRPS-16 Figure 4) compared to their WT and dipartite counterparts (∼86% of WT level, cf., Figure S2).^17^ Thus, to optimize these systems, we again targeted the SYNZIP pair 1:2 of NRPS-16 (Figure 4) – which was already the target of our GS linker insertion strategy but resulted in moderate optimization results (Figure S25).

As a rationale to guide our optimization attempt, we took the results from Reinke et al., who investigated the truncation of SYNZIPs (exemplified for SZ4) and its effect on their stability.^24^ They found that the N-terminal truncation of SZ4 did not affect its stability, while the C-terminal truncation noticeably destabilized SZ4. Consequently, we decided to truncate SZ1 (-6 and -14 AAs) N-terminally and SZ2 (-10 and -14 AAs) attached to subunits B and C, respectively, of NRPS-16. Coproduction of all modified and unmodified subunit B and C variants together with unmodified subunit A resulted in NRPS-17 to NRPS-24 (Figure 4). Of note, whereas the SYNZIP6 pair SZ1_-6_AAs and SZ2_-10_AAs was created to simulate the length of SZ17:18, the SYNZIPs pair SZ1_-14_AAs and SZ2_-14_AAa was created because, for SZ4, the two most N-terminal heptads were described as dispensable.^24^

All resulting assembly lines showed catalytic activity in biosynthesizing **1** in a range of 22–90 mg L^–1^. Of these, all but NRPS-18 and -19 resulted in a 17–210% increase in **1** compared to NRPS-16, with NRPS-23 (86 mg L^–1^) and -24 (90 mg L^–1^) showing the highest titers almost restoring WT XtpS production, highlighting the great potential of truncating SYNZIPs for type S NRPS optimization. However, to test the effects of further, more invasive truncations, we also attempted to remove 28 AAs from the N-terminus of SZ1 and 2 but found that the synthesis of **1** decreased to 62% compared to WT XtpS (SI Figure S3, NPRS-57), suggesting that the ideal truncation is probably in the range of 14 AAs. For more truncations, see SI Figures S27–S29. A comparative overview of all truncated SYNZIPs and their impact on peptide synthesis is shown in SI Figure S30. In brief, truncation of SZ2:19 in NRPS-14 resulted in an increased production of four constructs (NRPS-58, -61, -63, and -64) with NRPS-64 even restoring the synthesis of **1** to WT levels (Figure S27; NRPS-58 to -65). Truncation of SZ17:18 at the C–A position of XtpS (NRPS-1), however, resulted in strongly decreased yields of **1** (3–25 mg L^–1^; Figure S5: NRPS-66 to -80), indicating that truncation of SZ17:18 is not recommendable.

**Figure 5:**
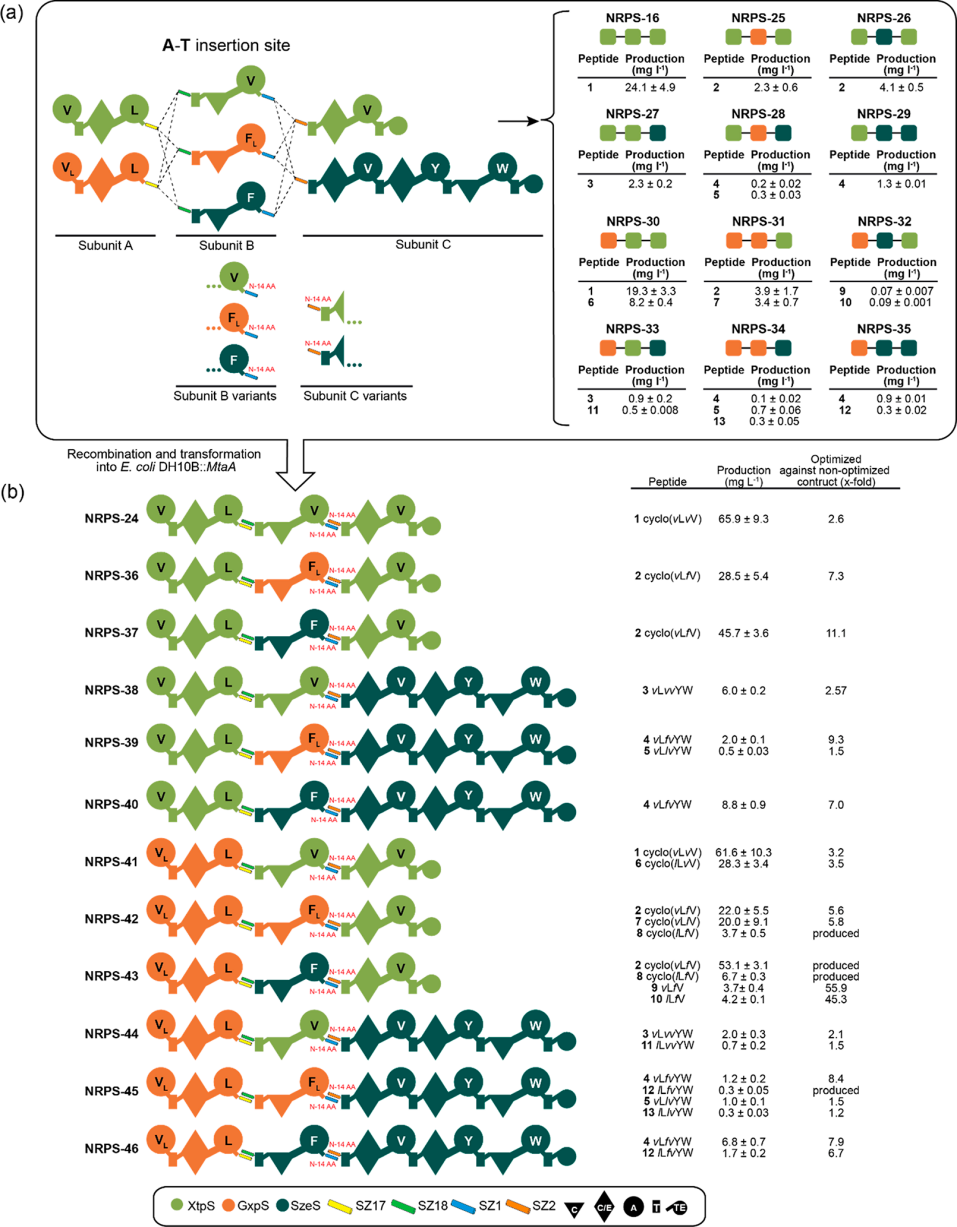
Optimized tripartite NRPS library. Production titers of nonoptimized constructs NRPS-16 and NRPS-36 to -46 are shown on the upper right corner. Subunits B and C were modified by removing 14 AAs from the N-terminal site of SZ1 and SZ2. Generated modified subunits were recombined with nonmodified subunits A and transformed into *E. coli* DH10B::*mtaA* to obtain NRPS-24 to -46. Production titers of NRPS-24 to -46 were compared with nonoptimized NRPS-15 to -35. Corresponding peptide yields (mg/L) and standard deviations are obtained from biological triplicate experiments. Domain assignment is as described before. For domain assignment, the following symbols are used: (A, large circles), (T, rectangle), (C, triangle), (C/E, diamond), (TE, small circle); substrate specificities are assigned for all A-domains and indicated by capital letters.

Lastly, we applied the identified best-performing SZ1:2 variant to our previously published tripartite SYNZIP library to determine whether the observed product-yield-increasing changes are exclusively linked to Xtps type S assembly line variants or whether there is an observable generality in this approach. To this end, we modified all functional (12 of 18) type S NRPS subunit B and subunit C variants by removing 14 AAs from the N-terminal sites of SZ1 and SZ2, respectively. As shown in Figure 5, all optimized type S NRPSs resulted in a strong increase in production with an optimization between 2- to 56-fold, ranging from 123.2 to 5592.4% compared to the nonoptimized constructs. These results not only confirm the apparent correlation between the length of SYNZIPs and the productivity of type S NRPSs but also infer the general applicability of this particular optimized SYNZIP pair for the generation of high-yield artificial type S assembly lines rather than being exclusively tied to a particular NRPS. In conclusion, truncating the SYNZIPs can completely eliminate their adverse effects, allowing SYNZIPs to be used without any restrictions for the generation of reprogrammed NRPSs.

### Other SYNZIP Networks

**Figure 6:**
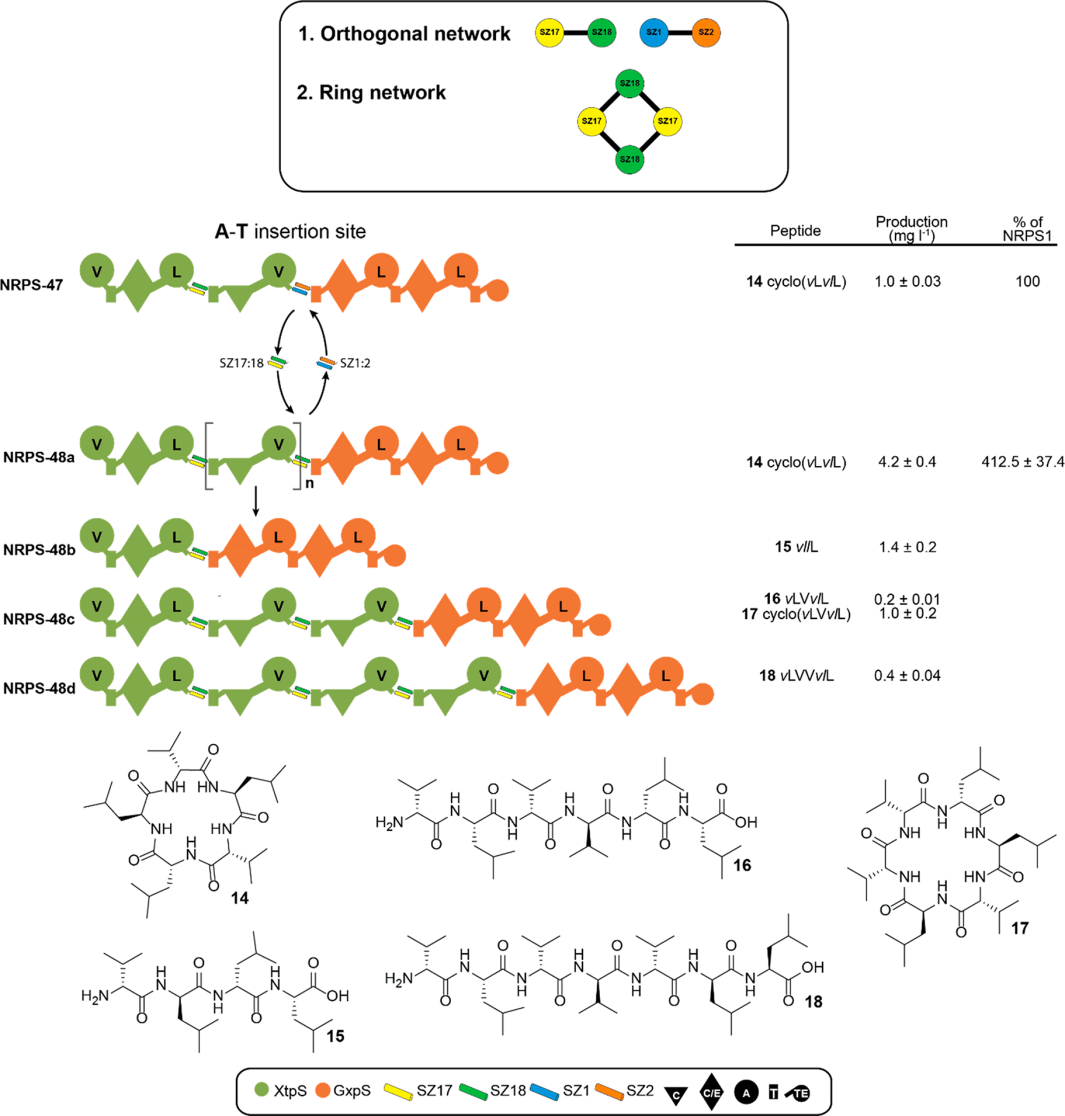
Introduction of a ring network. (A) Applied orthogonal and ring networks. SZ17:18 and SZ1:2 form an orthogonal network, meaning that both SYNZIPs do not interact with each other. Introducing two SZ17:18 pairs results in a ring network whereby applied SYNZIPs cross-talk. (B) For the construction of a ring interaction network, SZ1:2 in NRPS-47 was changed against SZ17:18, resulting in NRPS-48. NRPS-48 was capable to incorporate subunit B not at all or up to three times (NRPS-18a to -18c), leading to the production of peptides **15**–**18** shown at the bottom. Corresponding peptide yields (mg/L) and standard deviations are obtained from biological triplicate experiments. For domain assignment, the following symbols are used: (A, large circles), (T, rectangle), (C, triangle), (C/E, diamond), (TE, small circle); substrate specificities are assigned for all A-domains and indicated by capital letters.

As SYNZIPs have several interaction partners providing access to distinct interaction networks,^24,25^ we tried to establish the SYNZIP-mediated *in vivo* assembly of type S NRPSs beyond tripartite assembly lines by establishing a ring network. Initially, when we created the tripartite library (Figure 5), we decided to choose the orthogonal network, in which applied SYNZIPs 17:18 and 1:2 cannot communicate with each other. To demonstrate the applicability of other SYNZIP networks for NRPS engineering, we recreated a previously constructed type S NRPS^17^ (NRPS-47), assembled from building blocks of XtpS and the GameXPeptide synthetase (GxpS) from *Photorhabdus luminescens* TT01,^30^ by replacing SZ1:2 by SZ17:18 to generate NRPS-48 (Figure 6). With two SZ17:18 pairs, cross-talk between both type S NRPSs should be possible, theoretically leading to no or multiple incorporations of subunit B in NRPS-48. HPLC-MS analysis of extracts from NRPS-48 producing cultures indeed suggested none or multiple, up to three times, use of subunit B (NRPS-48a to -48d), resulting in the production of peptides **15**–**18**, which are not synthesized by NRPS-47 (Figure 6). Additionally, with these results we were able to demonstrate that we can build not only functional di- or tripartite type S NRPSs but also functional pentapartite systems that theoretically allow for almost inconceivably large combinatorics. With 16 building blocks per subunit already, more than a million new NRP combinations can be generated. With just a few more building blocks, this number can be driven exponentially into previously unimaginable new dimensions.

## Conclusion

In the field of natural products research, there are only a few examples of successfully applied SynBio for the development of novel drugs or their manufacturing, such as SynBio’s first malaria drug artemisinin.^32^ Large-scale bioengineering of NRPSs using SYNZIPs is thus a promising strategy for obtaining a variety of new valuable natural products, but as with many new technologies, there are limitations, namely, low yields for some type S NRPS constructs. To overcome this bottleneck turning type S NRPSs into a valuable tool for the production of NRPs with high yields and the development of novel bioactive molecular scaffolds with high confidence, we pursued two strategies: first, the insertion of flexible and unstructured GS stretches (Figure 2); and second, the targeted truncation of available SYNZIP pairs (Figures 4 and 5). With those approaches, we aimed to reduce the presumably introduced rigidity of type S NRPSs – probably caused by the insertion of the structurally stable α-helical SYNZIPs that can have a size of up to 54 AAs – while simultaneously enhancing the highly dynamic domain–domain interactions of NRPSs during the catalytic biosynthesis cycle. In particular, the possibility of introducing a plethora of distinct SYNZIP pairs into three interdomain linker regions increases the likelihood of creating impaired type S NRPSs, making efficient, rational optimization strategies necessary. With the iterative optimization strategy outlined in this paper, we have not only presented two extremely efficient SYNZIP pairs (SZ17:18 & SZ1:2) for the generation of di- and tripartite type S NRPSs but also paved the way for the rapid optimization of other SYNZIP pairs of interest.

Currently, SZ17:18 with 42 and 41 AAs is not only the shortest readily available pair but also the most efficient to generate unimpaired high-yielding type S NRPSs that even outcompete WT NRPSs (cf., NRPS-13, Figure 3). These extraordinary capabilities of SZ17:18, if introduced correctly (cf. NRPS-1 vs NRPS-10, Figure 2), also appeared in our proof of concept study, in which we compared covalently fused recombinant NRPSs with analogues of SYNZIP-linked type S variants to examine the impact of SZ17:18. Even with the introduction of the respective unoptimized SYNZIP pair (cf., Figure 2, NRPS-1) into recombinant NRPSs, observed peptide yields did not decrease compared to the recombinant covalent counterparts. Moreover, truncating SZ17:18 in NRPS-1 (Figure S5) led to drastically reduced production of **1**, indicating its ideally suited biophysical character for NRPS bioengineering purposes. We therefore recommend using the length of SZ17:18 as a guide for the optimization of other SYNZIP pairs.

Nevertheless, to optimize further SYNZIP pairs, the ideal length and composition should still be determined experimentally for every unique SYNZIP pair analogous to the workflow presented here. Noteworthy, in prior work,^18^ we observed decreasing peptide production to ∼30% compared to WT levels at the C–A position upon the insertion of SZ17:18 in XtpS (NRPS-1), which, however, was not due to the length of SZ17:18 but rather caused by the deletion of the native 10 AAs of the particular C–A linker region. This AA stretch has been deleted because we assumed that maintaining the native distance of the C- and A-domains is essential. Apparently, we underestimated the structural flexibility of NRPSs and noticed that once the native AAs were reinserted, WT-level peptide production could be restored (cf. NRPS-10, Figure 2). Therefore, we would like to revise our initial design and recommend keeping the native C–A linker AAs in type S NRPSs and choosing the fusion site for SYNZIP insertion as depicted in Figure S2.

In contrast to SZ17:18, unmodified SZ1:2 is significantly longer, resulting in a reduced production of peptides in several constructs. The detrimental impact of SZ1:2 is also apparent in NRPS-17 (Figure 4). Replacing SZ1:2 with SZ17:18 in NRPS-18 resulted in a 4-fold increase in production. Truncating SZ1:2 by 14 AAs restored production to WT levels (NRPS-24) and increased the productivity ∼100-fold in SZ1:2-optimized chimeric type S NRPSs (NRPS-24 to -46) compared to nonoptimized assembly lines (NRPS-16).

In case the truncation of any other inserted SYNZIP pair does not lead to an increased or restored peptide production, or if the truncated SYNZIPs lose their stability and thus affinity, we strongly recommend using GS stretches to increase the enzyme’s spatial flexibility (Figure 1). The reduced productivity does not exclusively depend on the length of applied SYNZIP but also on the insertion point itself and the targeted NRPS system, which might not provide enough spatial flexibility to allow the efficient progressing of the catalytic cycle.

Last but not least, the implementation of the ring network has opened up exciting prospects in the field of NRPS research. The generation of bi-, tri-, tetra-, and pentapartite NRPSs has expanded the realm of (bio)combinatorial possibilities to unprecedented levels. This breakthrough opens avenues to the creation of peptide libraries containing thousands to millions of distinct type S NRPS constructs. By systematically exchanging SYNZIP-linked NRPS units, we can gain insights into structure–activity relationships, the compatibility of building blocks, and the functionality of individual components. These advancements will drive the rational design of peptides and enzymes for tailored applications. In our view, type S NRPS peptide libraries possess tremendous potential to propel NRPS engineering and revolutionize NRPS-based drug discovery. This potential becomes even more significant as we continue to expand the scope of biocombinatorial possibilities. By harnessing the power of these libraries, we can expect substantial advancements in the field, leading to innovative approaches and breakthroughs in drug development.

## Material and Methods

### Cultivation of Strains

Cultivation was done as described before:^18^ All *E. coli*, *Photorhabdus*, and *Xenorhabdus* strains were cultivated in LB (10 g/L Tryptone, 5 g/L yeast extract, 10 g/L NaCl, pH 7.5) or TB liquid medium (12 g/L tryptone, 24 g/L yeast extract, 0.4% (v/v) glycerin, 10% (v/v), 17 mM KH_2_PO_4_, 72 mM K_2_HPO_4_, pH 6.5) at 37 °C (*E. coli*) or 30 °C (*Photorhabdus*, *Xenorhabdus*) for 16–18 h at 160–200 rpm. 1% (w/v) agar was added for growth on solid LB. If necessary, medium was supplemented 1:1000 with kanamycin (50 μg/mL in sterile ddH_2_O), chloramphenicol (34 μg/mL in ethanol), and/or spectinomycin stock solution (50 mg/mL in sterile ddH_2_O). For short-time storage, LB agar plates were stored either at 4 °C (*E. coli*) or 18 °C (*Photorhabdus*, *Xenorhabdus*). For permanent storage, liquid cultures were supplemented with 20% (v/v) glycerol and frozen at −80 °C.

### Plasmid Assembly

Genomic DNA from *Xenorhabdus* and *Photorhabdus* was isolated using the Gentra Puregene Yeast/Bact Kit (Qiagen) according to the manufacturers’ instruction for Gram negative bacteria. Plasmid DNA was isolated using PureYield Plasmid Miniprep System (Promega). PCRs were performed with oligonucleotides obtained from Eurofins Genomics (Table S4) containing homology arms of ∼20 bp in a one- or two-step PCR program. Phusion Hot Start Flex (New England Biolabs) was applied as High Fidelity DNA Polymerase and used accordingly to the manufacturers’ instruction. PCR fragments were digested with DpnI (Thermo Fisher Scientific). Purification of all fragments was performed with Monarch PCR & DNA Cleanup Kit or from 1% TAE agarose gel using Monarch Gel Extraction Kit. Plasmid assembly was done by HiFi (New England Biolabs) or Hot Fusion cloning, and DNA mix was transformed into *E. coli* DH10B via electroporation. Cells were regenerated in LB for 1 h at 37 °C and plated on LB agar plates containing appropriate antibiotics. Plasmids were isolated an verified by plasmid digest and DNA sequencing using Sanger sequencing (Eurofins Genomics).

### Heterologous Expression of NRPS Templates and HPLC-MS Analysis

Constructed plasmids were transformed into *E. coli DH10B*::mtaA, and cells from one colony were grown overnight in LB medium containing all necessary antibiotics (50 μg/mL kanamycin, 34 μg/mL chloramphenicol, and 50 μg/mL spectinomycin). 100 μL of the overnight culture was used to inoculate 10 mL of LB medium containing antibiotics, 0.002 mg/mL l-arabinose, and 2% (v/v) XAD-16. After 72 h at 22 °C, XAD-16 beads were harvested and incubated with one culture volume of methanol for 60 min at 180 rpm. The organic phase was filtrated, and extracts were evaporated to dryness. With 1 mL of MeOH, extracts were resolved, centrifuged for 20 min, and diluted 1:10 for HPLC-MS analysis. Liquid chromatography was performed on an UltiMate 3000 LC system (Dionex) with an installed C18 column (ACQUITY UPLCTM BEH, 130 Å, 2.1 × 100 mm, Waters). Separation was conducted at a flow rate of 0.4 mL/min using acetonitrile (ANC) and water containing 0.1% formic acid (v/v) in a 5–95% gradient over 16 min. Mass spectrometric analyses were performed using an ESI ion-trap mass spectrometer (AmaZon X, Bruker) or ESI. ESI-MS spectra were recorded in positive-ion mode with the mass range from 100 to 1200 *m*/*z* and ultraviolet (UV) at 200–600 nm. Evaluation was performed using DataAnalysis version 4.3 software (Bruker).

### Peptide Quantification

All of the peptides were quantified by generating a calibration curve. 10 different concentrations of a synthetic standard were measured by HPLC-MS, and the peak areas were plotted to the corresponding concentrations..^4^ Synthetic standard **1** (for the quantification of **1**, **2**, **6**, **7**, and **8**), **3** (for the quantification of **4**, **5**, **9**, **10**, and **11**), and **12** (for the quantification of **12**) were obtained from Synpeptide. Synthetic standards **15**, **16**, **17**, and **18** were synthesized as described below.

### Chemical Synthesis

Peptide synthesis was performed automatically with the Syro Wave peptide synthesizer (Biotage, Sweden) using standard Fmoc/*t*-Bu chemistry on a 25 or 50 μM scale. Fmoc amine-protected AAs in dimethylformamide (DMF) was added to preloaded H-AAn-2-CT resin, and the coupling reaction was performed by adding HCTU (*O*-(6-chlorobenzotriazol-1-yl)-*N*,*N*,*N*′,*N*′-tetramethyluronium hexafluorophosphate) in DMF (25 μmol: 250 μL, 0.54 mol/L, 5.4 equiv; 50 μmol: 500 μL, 0.27 mol/L, 2.7 equiv) and DIPEA (*N*,*N*-diisopropylethylamine) in NMP for 50 min alternating between shaking (15 s) and pausing (2 min). Washing the resin with 800 μL of NMP was followed by adding the capping solution (0.45 mL of DIPEA, 0.95 mL of Ac_2_O, 40 mg of HOBt in 20 mL of NMP; 25 μmol: 500 μL; 50 μmol: 1000 μL) and incubating for 5 min (15 s shaking, 1 min pausing). The Fmoc protecting group was cleaved off by incubation with 40% piperidine in NMP (25 μmol: 300 μL; 50 μmol: 600 μL) for 3 min (shaking 10 s and pausing 1 min) and 20% piperidine in NMP for 10 min (shaking 10 s and pausing 2 min). Between each reaction step, resin was washed with 800 μL of NMP. After synthesis, the resin was washed 5 times each with NMP, DMF, and DCM and dried.

The peptide was cleaved off from the solid phase by adding the cleavage cocktail (1:4 HFIP (hexafluoroisopropanol)/DCM) for 1 h and rinsed twice with the cleavage cocktail afterward. The resin was removed by filtration, and the cleavage cocktail was evaporated. For intramolecular cyclization, the peptide was dissolved in DMF/DCM (25 μmol, 25 mL, 1 mM) and mixed with HATU (38 mg, 100 μmol, 4 equiv) and DIPEA (13 mg, 17 μL, 100 μmol, 4 equiv) followed by incubating for 20 min at 60 °C. The cyclized or linear peptide was dissolved in DMSO, DMF, and MeOH and purified by preparative HPLC (Pure chromatography system, Büchi). The purity was determined by HPLC-MS.

## Supporting Information Available

Table S1: ESI-MS data of all produced peptides; Table S2: Strains used in this work; Table S3: Plasmids used in this work; Table S4: Oligonucleotides used in this work; Figure S1: Advantages of type S NRPS; Figure S2: Other splicing positions; Figures S3–24: HPLC-MS data of compounds produced in *E. coli* DH10B::*mtaA*; Figure S25: More GS-optimized chimeric tripartite XtpS NRPSs split at the A–T position; Figure S26: SZ1:2 truncation of chimeric dipartite XtpS NRPSs split at the A–T position; Figure S27: SZ2:19 truncation of chimeric dipartite XtpS NRPSs split at the A–T position; Figure S28: SZ17:18 truncation of chimeric dipartite XtpS NRPSs split at the C–A position; Figure S29: SZ4:21 truncation of chimeric dipartite XtpS NRPSs split at the A–T position; Figure S30: Overview of truncated SYNZIPs (PDF)
