# Supplementary material for: Guidelines for Optimizing Type S Nonribosomal Peptide Synthetases
Source: ACS Synth Biol. 2023 Jul 31;12(8):2432–43. doi: 10.1021/acssynbio.3c00295 (PMC10443035; doi:10.1021/acssynbio.3c00295)
Supplement: Supplementary file 1 — sb3c00295_si_001.pdf [file sb3c00295_si_001.pdf]

Supporting Information

Guidelines for Optimizing Type S Non-Ribosomal Peptide Synthetases

Nadya Abbood[1,2], Juliana Effert[1], Kenan A. J. Bozhueyuek[1,2,3,7*], Helge B. Bode[1,2,4,5,6*]

1 Max-Planck-Institute for Terrestrial Microbiology, Department of Natural Products in Organismic Interactions, 35043, Marburg, Germany.

2 Molecular Biotechnology, Institute of Molecular Biosciences, Goethe University Frankfurt, 60438, Frankfurt am Main, Germany.

3 Myria Biosciences AG, Mattenstrasse 26, 4058 Basel, Switzerland

4 Chemical Biology, Department of Chemistry, Philipps-University Marburg, 35043, Marburg, Germany

5 Senckenberg Gesellschaft für Naturforschung, 60325, Frankfurt am Main, Germany

6 Center for Synthetic Microbiology (SYNMIKRO), Phillips University Marburg, 35043 Marburg, Germany

7 Present address: Synthetic Biology of Microbial Natural Products, Helmholtz Institute for Pharmaceutical Research Saarland (HIPS), Helmholtz Centre for Infection Research, Saarland University Campus, 66123 Saarbrücken, Germany

* Corresponding author: kenan.bozhueyuek@myria.bio; helge.bode@mpi-marburg.mpg.de

1. Supplementary tables 4
Table S1. ESI-MS data of all produced peptides. 4
Table S2. Strains used in this work. 4
Table S3. Plasmids used in this work. 4
Table S4. Oligonucleotides used in this work. 7
2. Supplementary Figures 10
Figure S1. Advantages of Type S NRPS. 10
Figure S2. Other splicing positions.. 11
Figure S3. HPLC/MS data (Figure 2) of compound 1 produced in *E. coli* DH10B::*mtaA*. 13
Figure S4. HPLC/MS data (Figure 3) of compounds 1 produced in *E. coli* DH10B::*taA*. 14
Figure S5. HPLC/MS data (Figure 4) of compound 1 produced in *E. coli* DH10B::*mtaA*. 16
Figure S6. HPLC/MS data (Figure 5) of compound 1 produced in *E. coli* DH10B::*mtaA*. 16
Figure S7. HPLC/MS data (Figure 5) of compound 2 produced in E. coli DH10B::mtaA. 17
Figure S8. HPLC/MS data (Figure 5) of compound 2 produced in *E. coli* DH10B::*mtaA*. 17
Figure S9. HPLC/MS data (Figure 5) of compound 3 produced in *E. coli* DH10B::*mtaA*. 17
Figure S10. HPLC/MS data (Figure 5) of compounds 4 and 5 produced in *E. coli* DH10B::*mtaA*. 18
Figure S11. HPLC/MS data (Figure 5) of compound 4 produced in *E. coli* DH10B::*mtaA*. 19
Figure S12. HPLC/MS data (Figure 5) of compounds 1 and 6 produced in E. coli DH10B::mtaA. 19
Figure S13. HPLC/MS data (Figure 5) of compounds 2, 7 and 8 produced in *E. coli* DH10B::*mtaA*. 20
Figure S14. HPLC/MS data (Figure 5) of compounds 2, 8, 9 and 10 produced in *E. coli* DH10B::*mtaA*. 21
Figure S15. HPLC/MS data (Figure 5) of compounds 3 and 11 produced in E. coli DH10B::mtaA. 22
Figure S16. HPLC/MS data (Figure 5) of compounds 4, 12, 5 and 13 produced in *E. coli* DH10B::*mtaA*. 23
Figure S17. HPLC/MS data (Figure 5) of compounds 4 and 12 produced in *E. coli* DH10B::*mtaA*. 24
Figure S18. HPLC/MS data (Figure 6) of compound 14 produced in *E. coli* DH10B::*mtaA*. 24
Figure S19. HPLC/MS data (Figure 5) of compounds 14, 15, 16, 17 and 18 produced in *E. coli* DH10B::*mtaA*. 26
Figure S20. HPLC/MS data (Figure S25) of compound 1 produced in *E. coli* DH10B::*mtaA*. 28
Figure S21. HPLC/MS data (Figure S26) of compound 1 produced in E. coli DH10B::mtaA. 28
Figure S22. HPLC/MS data (Figure S27) of compound 1 produced in *E. coli* DH10B::*mtaA*. 30
Figure S23. HPLC/MS data (Figure S28) of compound 1 produced in *E. coli* DH10B::*mtaA*. 33
Figure S24. HPLC/MS data (Figure S29) of compound 1 produced in *E. coli* DH10B::*mtaA*. 34
Figure S25. More GS-optimized chimeric tri-partite XtpS NRPSs split at the A-T position. 36
Figure S26. SZ1:2 truncation of chimeric di-partite XtpS NRPSs split at the A-T position. 36
Figure S27. SZ2:19 truncation of chimeric di-partite XtpS NRPSs split at the A-T position. 38
Figure S28. SZ17:18 truncation of chimeric di-partite XtpS NRPSs split at the C-A position. 40
Figure S29. SZ4:21 truncation of chimeric di-partite XtpS NRPSs split at the A-T position. 41

Figure S30. Overview of truncated SYNZIPs.. 43
3. References 43

1. Supplementary tables

Table S1. ESI-MS data of all produced peptides.

Peptide (#)	mass-to-charge ratio (*m/z*)	Molecular formula	AA sequence	Reference
**1**	411.29	$C_{21}H_{38}O_4N_4$	cyclo(*v*L*v*V)	[1]
**2**	459,30	$C_{25}H_{38}N_4O_4$	cylco(*v*L*f*V)	[2]
**3**	778,45	$C_{41}H_{59}N_7O_8$	*v*L*vv*YW	[2]
**4**	826.45	$C_{45}H_{59}N_7O_8$	*v*L*fv*YW	[2]
**5**	792.47	$C_{42}H_{61}N_7O_8$	*v*L*lv*YW	[2]
**6**	425.31	$C_{22}H_{40}N_4O_4$	cyclo(*l*L*v*V)	[2]
**7**	425,31	$C_{22}H_{40}N_4O_4$	cyclo(*v*L*l*V)	[2]
**8**	472.31	$C_{26}H_{40}N_4O_4$	cyclo(*l*L*f*V)	[2]
**9**	476.62	$C_{25}H_{40}N_4O_5$	*v*L*f*V	[2]
**10**	490.65	$C_{26}H_{42}N_4O_5$	*l*L*f*V	[2]
**11**	792.47	$C_{42}H_{61}N_7O_8$	*l*L*vv*YW	[2]
**12**	840.47	$C_{46}H_{61}N_7O_8$	*l*L*fv*YW	[2]
**13**	806.48	$C_{43}H_{63}N_7O_8$	*l*L*lv*YW	[2]
**14**	538.40	$C_{28}H_{51}O_5N_5$	cyclo(*v*L*vl*L)	[2]
**15**	470.35	$C_{24}H_{46}O_5N_4$	*vl*L	this study
**16**	655.47	$C_{33}H_{62}O_7N_6$	*v*LV*vl*L	this study
**17**	637.46	$C_{33}H_{60}O_6N_6$	cyclo(*v*LV*vl*L)	this study
**18**	754.54	$C_{38}H_{71}O_8N_7$	*v*LVV*vl*L	this study

Table S2. Strains used in this work.

Strain	Genotype/ NRPS	Reference
E. coli DH10B	F_mcrA (*mrr-hsd*RMS-*mcr*BC), 80*lac*ZΔ, M15, Δ*lac*X74 *rec*A1 *end*A1 *ara*D 139Δ(*ara*, *leu*)7697 *gal*U *gal*K λ *rps*L (*Strr*) *nup*G / -	[3]
E. coli DH10B::*mta*A	DH10B with *mtaA* from pCK_*mtaA*Δ*entD* / -	[4]
P. luminescens TTO1	- / *gxpS*	DSMZ
X. nematophila ATCC 19061	- / *xtpS*	ATCC
X. szentirmaii DSM 16338	- / *szeS*	DSMZ

Table S3. Plasmids used in this work.

Plasmids	Genotype	Reference
pCOLA_ara/tacI	ori ColA, kan^R, *araC-P*$_{BAD}$ and *tac*I	unpublished
pCK_0402	ori p15A, cm^R, *araC-P*$_{BAD}$ and *tac*I-*araE*	[5]

pCOLA_ara_xtpS_tacI_JW	ori ColA, kan^R, *araC-P$_{BAD}$ xtpS* and *tac*I	[5]
pCOLA_ara_gxpS_tacI_JW	ori ColA, kan^R, *araC-P$_{BAD}$ gxpS* and *tac*I	[5]
pNA2	ori p15A, cm^R, *araC-P$_{BAD}$ xtpS_* $A_1T_1C/E_2A_2T_2$-SYNZIP17 und *tac*I-*araE*	[2]
pNA3	ori ColA, kan^R, *araC-P$_{BAD}$* SYNZIP18-*xtpS_* $C_3A_3T_3C/E_4A_4T_4$TE und *tac*I	[2]
pNA4	ori p15A, cm^R, *araC-P$_{BAD}$ xtpS_* A_1T_1C/E_2A_2-SYNZIP17 und *tac*I-*araE*	[2]
pNA5	ori ColA, kan^R, *araC-P$_{BAD}$* SYNZIP18-*xtpS_* $T_2C_3A_3T_3C/E_4A_4T_4$TE und *tac*I	[2]
pNA8	ori p15A, cm^R, *araC-P$_{BAD}$ xtpS_*$A_1T_1C/E_2A_2T_2C_3$-(GS)$_5$-SYNZIP17 and *tac*I-*araE*	this study
pNA9	ori p15A, cm^R, *araC-P$_{BAD}$ xtpS_*$A_1T_1C/E_2A_2T_2C_3$-(GS)$_4$-SYNZIP17 and *tac*I-*araE*	this study
pNA10	ori p15A, cm^R, *araC-P$_{BAD}$ xtpS_*$A_1T_1C/E_2A_2T_2C_3$-(GS)$_2$-SYNZIP17 and *tac*I-*araE*	this study
pNA15	ori ColA, kan^R, *araC-P$_{BAD}$* SYNZIP18-*xtpS_*$T_2C_3A_3$-SYNZIP1 and *tac*I	[2]
pNA16	ori CloDF13, spec^R, *araC-P$_{BAD}$* SYNZIP2-*xtpS_*$T_3C/E_4A_4T_4$TE and *tac*I	[2]
pNA17	ori ColA, kan^R, *araC-P$_{BAD}$* SYNZIP18-*xtpS_*$C_3A_3T_4$-SYNZIP1 and *tac*I	[2]
pNA18	ori CloDF13, spec^R, *araC-P$_{BAD}$* SYNZIP2-*xtpS_*$C/E_4A_4T_4$TE and *tac*I	[2]
pNA26	ori p15A, cm^R, *araC-P$_{BAD}$ gxpS_*A_1T_1C/E_2A_2-SYNZIP17 and *tac*I-*araE*	[2]
pNA27	ori ColA, kan^R, *araC-P$_{BAD}$* SYNZIP18-*gxpS_*$T_2C_3A_3$-SYNZIP1 and *tac*I	[2]
pNA30	ori ColA, kan^R, *araC-P$_{BAD}$* SYNZIP18-*szeS_*$T_2C_3A_3$-SYNZIP1 and *tac*I	[2]
pNA31	ori CloDF13, spec^R, *araC-P$_{BAD}$* SYNZIP2-*szeS_*$T_3C/E_4A_4T_4$ $C/E_5A_5T_5C_6A_6T_6$TE and *tac*I	[2]
pNA40	ori p15A, cm^R, *araC-P$_{BAD}$ xtpS_* A_1T_1C/E_2A_2-SYNZIP2 und *tac*I-*araE*	this study
pNA41	ori ColA, kan^R, *araC-P$_{BAD}$* SYNZIP19-*xtpS_* $T_2C_3A_3T_3C/E_4A_4T_4$TE und *tac*I	this study
pNA42	ori p15A, cm^R, *araC-P$_{BAD}$ xtpS_* A_1T_1C/E_2A_2-SYNZIP21 und *tac*I-*araE*	this study
pNA43	ori ColA, kan^R, *araC-P$_{BAD}$* SYNZIP4-*xtpS_* $T_2C_3A_3T_3C/E_4A_4T_4$TE und *tac*I	this study
pNA72	ori p15A, cm^R, *araC-P$_{BAD}$ xtpS_* A_1T_1C/E_2A_2-N-terminally truncated SYNZIP2 (-9 AA) und *tac*I-*araE*	this study
pNA73	ori ColA, kan^R, *araC-P$_{BAD}$* N-terminally truncated SYNZIP19 (-2 AA)-*xtpS_* $T_2C_3A_3T_3C/E_4A_4T_4$TE und *tac*I	this study
pNA145	ori p15A, cm^R, *araC-P$_{BAD}$ xtpS_*$A_1T_1C/E_2A_2T_2C_3$-N-terminally truncated SYNZIP17 (-7 AA) and *tac*I-*araE*	this study
pNA146	ori ColA, kan^R, *araC-P$_{BAD}$* N-terminally truncated SYNZIP18 (-7 AA)-*xtpS_*$A_3T_3C/E_4A_4T_4$TE and *tac*I	this study
pNA147	ori p15A, cm^R, *araC-P$_{BAD}$ xtpS_*$A_1T_1C/E_2A_2T_2C_3$- N-terminally truncated SYNZIP17 (-14 AA) and *tac*I-*araE*	this study
pNA148	ori ColA, kan^R, *araC-P$_{BAD}$* N-terminally truncated SYNZIP18 (-14 AA)-*xtpS_*$A_3T_3C/E_4A_4T_4$TE and *tac*I	this study

pNA149	ori ColA, kan^R, *araC-P_{BAD}* SYNZIP18-*xtpS*_$T_2C_3A_3$- N-terminally truncated SYNZIP1 (-14 AA) and *tac*I	this study
pNA150	ori CloDF13, $spec^R$, *araC-P_{BAD}* N-terminally truncated SYNZIP2 (-14 AA)-*xtpS*_$T_3C/E_4A_4T_4TE$ and *tac*I	this study
pNA151	ori ColA, kan^R, *araC-P_{BAD}* N-terminally truncated SYNZIP4 (-14 AA)-*xtpS*_ $T_2C_3A_3T_3C/E_4A_4T_4TE$ und *tac*I	this study
pNA152	ori ColA, kan^R, *araC-P_{BAD}* SYNZIP18-*xtpS*_$T_2C_3A_3$-SYNZIP17 and *tac*I	this study
pNA153	ori CloDF13, $spec^R$, *araC-P_{BAD}* SYNZIP18-*xtpS*_$T_3C/E_4A_4T_4TE$ and *tac*I	this study
pNA154	ori ColA, kan^R, *araC-P_{BAD}* SYNZIP18-$(GS)_5$-*xtpS*_$A_3T_3C/E_4A_4T_4TE$ and *tac*I	this study
pNA155	ori ColA, kan^R, *araC-P_{BAD}* SYNZIP18-$(GS)_2$-*xtpS*_$A_3T_3C/E_4A_4T_4TE$ and *tac*I	this study
pNA156	ori p15A, cm^R, *araC-P_{BAD} xtpS*_$A_1T_1C/E_2A_2T_2C_3$-SYNZIP17-(NATETVYPES) and *tac*I-*araE*	this study
pNA157	ori ColA, kan^R, *araC-P_{BAD}* SYNZIP18-*xtpS*_$T_2C_3A_3$-$(GS)_5$-SYNZIP1 and *tac*I	this study
pNA158	ori ColA, kan^R, *araC-P_{BAD}* SYNZIP18-*xtpS*_$T_2C_3A_3$-$(GS)_2$-SYNZIP1 and *tac*I	this study
pNA159	ori CloDF13, $spec^R$, *araC-P_{BAD}* SYNZIP2-$(GS)_5$-*xtpS*_$T_3C/E_4A_4T_4TE$ and *tac*I	this study
pNA160	ori CloDF13, $spec^R$, *araC-P_{BAD}* SYNZIP2-$(GS)_2$-*xtpS*_$T_3C/E_4A_4T_4TE$ and *tac*I	this study
pNA161	ori p15A, cm^R, *araC-P_{BAD} xtpS*_ A_1T_1C/E_2A_2-N-terminally truncated SYNZIP2 (-14 AA) und *tac*I-*araE*	this study
pNA162	ori ColA, kan^R, *araC-P_{BAD}* N-terminally truncated SYNZIP19 (-7 AA)-*xtpS*_ $T_2C_3A_3T_3C/E_4A_4T_4TE$ und *tac*I	this study
pNA163	ori p15A, cm^R, *araC-P_{BAD} xtpS*_$A_1T_1C/E_2A_2T_2C_3$- C-terminally truncated SYNZIP17 (-7 AA) and *tac*I-*araE*	this study
pNA164	ori ColA, kan^R, *araC-P_{BAD}* C-terminally truncated SYNZIP18 (-7 AA)-*xtpS*_$A_3T_3C/E_4A_4T_4TE$ and *tac*I	this study
pNA165	ori ColA, kan^R, *araC-P_{BAD}* SYNZIP18-*xtpS*_$T_2C_3A_3$-N-terminally truncated SYNZIP1(-28 AA) and *tac*I	this study
pNA166	ori CloDF13, $spec^R$, *araC-P_{BAD}* N-terminally truncated SYNZIP2 (-28 AA)-*xtpS*_$T_3C/E_4A_4T_4TE$ and *tac*I	this study
pNA167	ori ColA, kan^R, *araC-P_{BAD}* SYNZIP18-*gxpS*_$T_2C_3A_3$-N-terminally truncated SYNZIP1 (-14 AA) and *tac*I	this study
pNA168	ori ColA, kan^R, *araC-P_{BAD}* SYNZIP18-*szeS*_$T_2C_3A_3$-N-terminally truncated SYNZIP1 (-14 AA) and *tac*I	this study
pNA169	ori CloDF13, $spec^R$, *araC-P_{BAD}* N-terminally truncated SYNZIP2 (-14 AA)-*szeS*_$T_3C/E_4A_4T_4$ $C/E_5A_5T_5C_6A_6T_6TE$ and *tac*I	this study
pNA170	ori pUC19, kan^R, *araC-P_{BAD}* SYNZIP18-*xtpS*_$T_2C_3A_3$-SYNZIP17 and *tac*I	this study
pJW61	ori p15A, cm^R, *araC-P_{BAD} xtpS*_$A_1T_1C/E_2A_2T_2C_3$-SYNZIP17 and *tac*I-*araE*	[5]
pJW62	ori ColA, kan^R, *araC-P_{BAD}* SYNZIP18-*xtpS*_$A_3T_3C/E_4A_4T_4TE$ and *tac*I	[5]

Table S4. Oligonucleotides used in this work.

Plasmids	Oligo-nucleotides	Sequence (5' →3'; overlapping ends)	Template
pNA8	KB-pACYC-II-FW	AACGAGAAGGAGGAATTAAAATCG	pJW61
	na17_RV	CGATTTTAATTCCTCCTTCTCGTTTGATCCCGAACCTGAGCCGGATCCAGACCCCCAGGTTTTTAACAACAATGTGC	pJW61
pNA9	KB-pACYC-II-FW	AACGAGAAGGAGGAATTAAAATCG	pJW61
	na19_RV	CGATTTTAATTCCTCCTTCTCGTTCGAACCTGAGCCGGATCCAGACCCCCAGGTTTTTAACAACAATGTGC	pJW61
pNA10	KB-pACYC-II-FW	AACGAGAAGGAGGAATTAAAATCG	pJW61
	na20_RV	CGATTTTAATTCCTCCTTCTCGTTGGATCCAGACCCCCAGGTTTTTAACAACAATGTG	pJW61
pNA40	na3	TGGGCTAACAGGAGGAATTCCATGAAAGATAGCATGGCTAAAAAGGG	*X. nematophila* ATCC 19061
	na87	CTTACGCAGATACGCGTTACGCGCATAAATCTGGCGGGCGAA	*X. nematophila* ATCC 19061
	na85	GCTGGAACGTGATGAACAGAACCTGGAAAAAATCATCGCGAACCTGCGTGACGAAATCGCGCGTCTCGAAAACGAAGTTGCGTCTCACGAACAGTGACAATTAATCATCGGCTCG	pCK_0402
	na43	CATGGAATTCCTCCTGTTAGCC	pCK_0402
	na86	GCGCGTAACGCGTATCTGCGTAAGAAAATCGCACGTCTGAAAAAAGACAACCTGCAGCTGGAACGTGATGAACAGAAC	pNA40_BB1_SZ2 half
	na43	CATGGAATTCCTCCTGTTAGCC	pNA40_BB1_SZ2 half
pNA41	na90	GACGCGTACAAAAACCGTCTGGTTGCGCCACAAGGAGAA	*X. nematophila* ATCC 19061
	na7	CGAGCCGATGATTAATTGTCACAGCGCCTCCACTTCG	*X. nematophila* ATCC 19061
	jw61	TGACAATTAATCATCGGCTCG	pCOLA_ara/tacI
	na88	GTTTCTGTTTCAGCTGTTCACGTTTCTGCTTCAGCTCTTCGTTACGGTTCTTCAGTTCTTCTTTTTTGTTCTCCAGAGATTCCAGTTCGTTCATGGAATTCCTCCTGTTAGCC	pCOLA_ara/tacI
	jw61	TGACAATTAATCATCGGCTCG	pNA41_BB1_SZ19half
	na89	CAGACGGTTTTTGTACGCGTCCAGTTTGTTACGCAGAGCCGCCAGTTTCTGTTTCAGCTGTTCACG	pNA41_BB1_SZ19half
pNA42	na3	TGGGCTAACAGGAGGAATTCCATGAAAGATAGCATGGCTAAAAAGGG	*X. nematophila* ATCC 19061
	na93	TTCCAGCTGCGCAACTTCGTTATAAATCTGGCGGGCGAA	*X. nematophila* ATCC 19061
	na91	GCGTACCTGGAGAAGGAGATCGCGCGTCTGCGTAAAGAAATTGCGGCGCTGCGTGACCGTCTGGCGCACAAAAAATGACAATTAATCATCGGCTCG	pCK_0402
	na43	CATGGAATTCCTCCTGTTAGCC	pCK_0402
	na92	AACGAAGTTGCGCAGCTGGAAAACGACGTTGCGGTTATCGAAAATGAAAACGCGTACCTGGAGAAGGAGATC	pNA42_BB1_SZ21half
	na43	CATGGAATTCCTCCTGTTAGCC	pNA42_BB1_SZ21half
pNA43	na96	TGGAAAACGACGTTGCAGAAGTTGCGCCACAAGGAGAA	*X. nematophila* ATCC 19061
	na7	CGAGCCGATGATTAATTGTCACAGCGCCTCCACTTCG	*X. nematophila* ATCC 19061
	jw61	TGACAATTAATCATCGGCTCG	pCOLA_ara/tacI
	na102	CGTTACGATTCAGTTTAACCGCAACACGGTTTTTGAGTTCCGCAACTTTCTGCATGGAATTCCTCCTGTTAGCC	pCOLA_ara/tacI

	jw61	TGACAATTAATCATCGGCTCG	pNA41_BB1_SZ4 half
	na103	GCGTTACGGTTCTTCAGCTCTTCAACTTTGTTTTTCAGCTGTTCGTTACGATTCAGTTTAACCGC	pNA41_BB1_SZ4 half
	jw61	TGACAATTAATCATCGGCTCG	pNA41_BB2_SZ4 half
	na95	TTCTGCAACGTCGTTTTCCAGACGCGCAACCTCGTTCTCCAGGGTCGCCAGTTCGTTCTTGAGGTAAGCGTTACGGTTCTTCAGCTC	pNA41_BB2_SZ4 half
pNA72	na141	ATCGCACGTCTGAAAAAAGAC	pNA40
	na144	GTCTTTTTTCAGACGTGCGATATAAATCTGGCGGGCGAA	pNA40
pNA73	na143	CTGGAATCTCTGGAGAACAAAAAAG	pNA41
	Na145	CTTTTTTGTTCTCCAGAGATTCCAGCATGGAATTCCTCCTGTTAGCC	pNA41
pNA145	na304	CATTGTTGTTAAAAACCTGGTCGAAAAAGGCTGAATTGC	pJW61
	jw62	CCAGGTTTTTAACAACAATGTGC	pJW61
pNA146	na305	CTAACAGGAGGAATTCCATGCTGAAAGCCTTGGACCGC	pJW62
	jw64	CATGGAATTCCTCCTGTTAGCC	pJW62
pNA147	na306	CATTGTTGTTAAAAACCTGGAATCGCATCGAACAGTTAAAACAG	pJW61
	jw62	CCAGGTTTTTAACAACAATGTGC	pJW61
pNA148	na307	CTAACAGGAGGAATTCCATGTTAAATGCCATTGACAAAGAGCTG	pJW62
	jw64	CATGGAATTCCTCCTGTTAGCC	pJW62
pNA149	na308	GTTTGCCCGGCAGGTCTATAATGAGAACGAAACCCTGAAGAAAAAG	pNA125
	na286	ATAGACCTGCCGGGCAAAC	pNA125
pNA150	na309	CTAACAGGAGGAATTCCATGAAAGACAACCTGCAGCTGGAAC	pNA126
	na43	CATGGAATTCCTCCTGTTAGCC	pNA126
pNA151	na310	CTAACAGGAGGAATTCCATGAATCGTAACGAACAGCTGAAAAAC	pNA43
	jw64	CATGGAATTCCTCCTGTTAGCC	pNA43
pNA152	jw61	TGACAATTAATCATCGGCTCG	pNA15
	na286	ATAGACCTGCCGGGCAAAC	pNA15
	na311	GTTTGCCCGGCAGGTCTATAACGAGAAGGAGGAATTAAAATCG	pJW61
	na312	CGAGCCGATGATTAATTGTCACTTGTAGGCTTCGATCTCCTTACG	pJW61
pNA153	na315	CAAGCGCCACAAGGGGA	pNA28
	na43	CATGGAATTCCTCCTGTTAGCC	pNA28
	na313	GGCTAACAGGAGGAATTCCATGTTCTATGCTGAAGAGCGTGAACTG	pJW62
	na314	TTCCCCTTGTGGCGCTTGTGAGATAGCTGCAGTCAGCTCG	pJW62
pNA154	na316	AACGAGCTGACTGCAGCTATCTCAGGGTCTGGATCCGGCTCAGGTTCGGGATCATTATGTATTCATCAACTTTTTGAACAGC	pJW62
	na317	TGAGATAGCTGCAGTCAGCTC	pJW62
pNA155	na318	AACGAGCTGACTGCAGCTATCTCAGGTTCGGGATCATTATGTATTCATCAACTTTTTGAACAGC	pJW62
	na317	TGAGATAGCTGCAGTCAGCTC	pJW62
pNA156	KB-pACYC-II-FW	AACGAGAAGGAGGAATTAAAATCG	pJW61

	na319	CGATTTTAATTCCTCCTTCTCGTTCGATTCAGGATACACGGTTTCAGTGGCATTCCAGGTTTTTAACAACAATGTGC	pJW61
pNA157	na320	GTTTGCCCGGCAGGTCTATGGGTCTGGATCCGGCTCAGGTTCGGGATCAAACCTGGTTGCGCAGCTC	pNA15
	na286	ATAGACCTGCCGGGCAAAC	pNA15
pNA158	na321	GTTTGCCCGGCAGGTCTATGGTTCGGGATCAAACCTGGTTGCGCAGCTC	pNA15
	na286	ATAGACCTGCCGGGCAAAC	pNA15
pNA159	na322	GAAGTTGCGTCTCACGAACAGGGGTCTGGATCCGGCTCAGGTTCGGGATCAGCGGCTCCGCAGGG	pNA16
	na290	CTGTTCGTGAGACGCAACTTC	pNA16
pNA160	na323	GAAGTTGCGTCTCACGAACAGGGTTCGGGATCAGCGGCTCCGCAGGG	pNA16
	na290	CTGTTCGTGAGACGCAACTTC	pNA16
pNA161	na324	TTCGCCCGCCAGATTTATAAAGACAACCTGCAGCTGGAAC	pNA44
	na142	ATAAATCTGGCGGGCGAA	pNA44
pNA162	na325	GGCTAACAGGAGGAATTCCATGAACAAAAAAGAAGAACTGAAGAACCG	pNA45
	na43	CATGGAATTCCTCCTGTTAGCC	pNA45
pNA163	jw61	TGACAATTAATCATCGGCTCG	pJW61
	na326	CGAGCCGATGATTAATTGTCAACGCAGATTGGCGATCTTTTG	pJW61
pNA164	jw63	TTATGTATTCATCAACTTTTTGAACAGC	pJW62
	na327	GCTGTTCAAAAAGTTGATGAATACATAAGTTATCAAGGGCGCGAAGTT	pJW62
pNA165	na329	GTTTGCCCGGCAGGTCTATGACCTGATCGCGTACCTGG	pNA15
	na286	ATAGACCTGCCGGGCAAAC	pNA15
pNA166	na330	GGCTAACAGGAGGAATTCCATGAAAATCATCGCGAACCTGC	pNA16
	na43	CATGGAATTCCTCCTGTTAGCC	pNA16
pNA167	na331	AATGAGAACGAAACCCTGAAGAAAAAG	pNA27
	na333	CTTTTTCTTCAGGGTTTCGTTCTCATTGTAAGCTTGGCGAGCAAAGG	pNA27
pNA168	na331	AATGAGAACGAAACCCTGAAGAAAAAG	pNA30
	na332	CTTTTTCTTCAGGGTTTCGTTCTCATTATAATGCTGACGGGCAAACG	pNA30
pNA169	na309	CTAACAGGAGGAATTCCATGAAAGACAACCTGCAGCTGGAAC	pNA31
	na43	CATGGAATTCCTCCTGTTAGCC	pNA31

2. Supplementary Figures

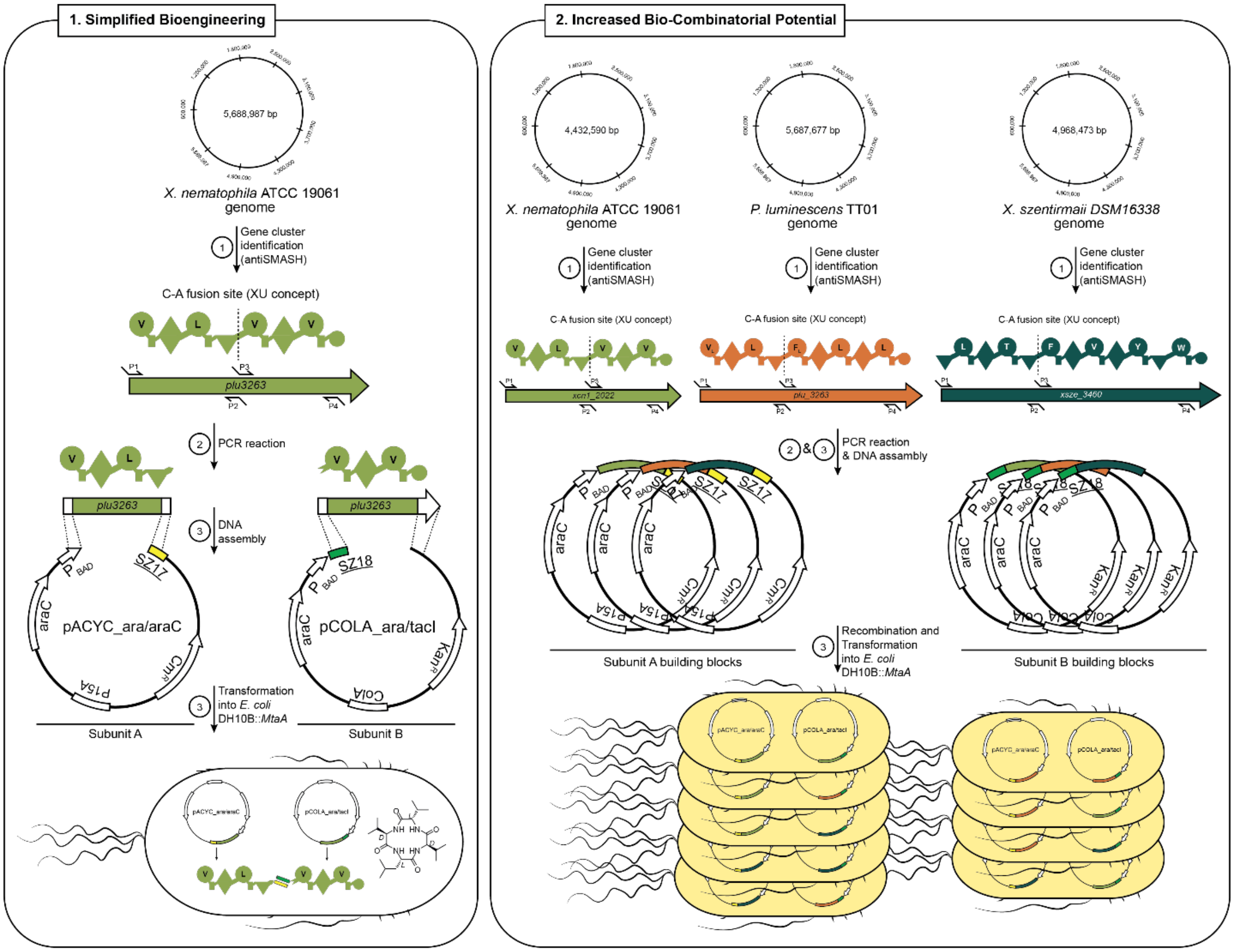

Figure S1. Advantages of Type S NRPS. 1) Simplified bioengineering: Splitting NRPS into two or three independently expressed SYNZIP linked subunits enables easier and faster cloning. Traditional NRPS engineering often requires elaborated cloning strategies (yeast cloning,[6] LLHR[7] or ExoCET[8]) which are frequently accompanied with technical problems and limitation. By breaking NRPSs into smaller subunits, cloning can be simplified, making standard strategies such as Gibson[9], HiFi and Hot Fusion[10] assembly sufficient. **2) Increased bio-combinatorial potential:** With SYNZIPs, type S NRPSs can be created faster and to a greater extent than before, as the number of artificial NRPSs increases exponentially with the number of subunits. Once generated, subunits can be reused at any time and for any experimental approaches without any additional cloning efforts.

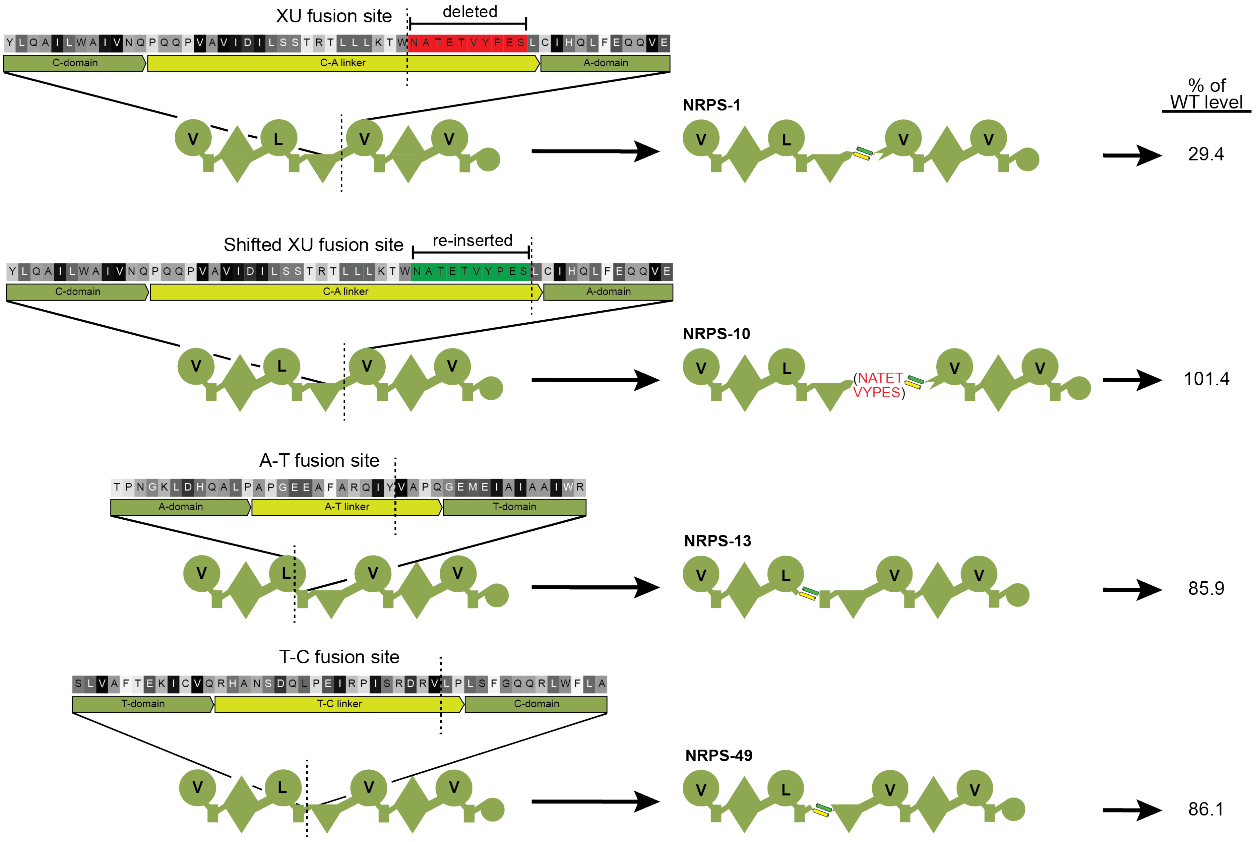

Figure S2. Other splicing positions. SZ17:18 Introduction at three different positions within the C-A, T-C and A-T linker region to create two protein type S XtpS variants. AS sequences and exact SZ17:18 introduction sites are highlighted with a vertical dashed line. Initially, for the introduction of SYNZIPs into the C-A position, 10 AAs were deleted (highlighted in red) to meet the distance between the C- and A-domain. Re-insertion of the 10 AAs (highlighted in green) and shifted fusion site restored peptide production. Production of 1 (cyclo (vLvV)) relative to WT level are indicated on the right hand site.

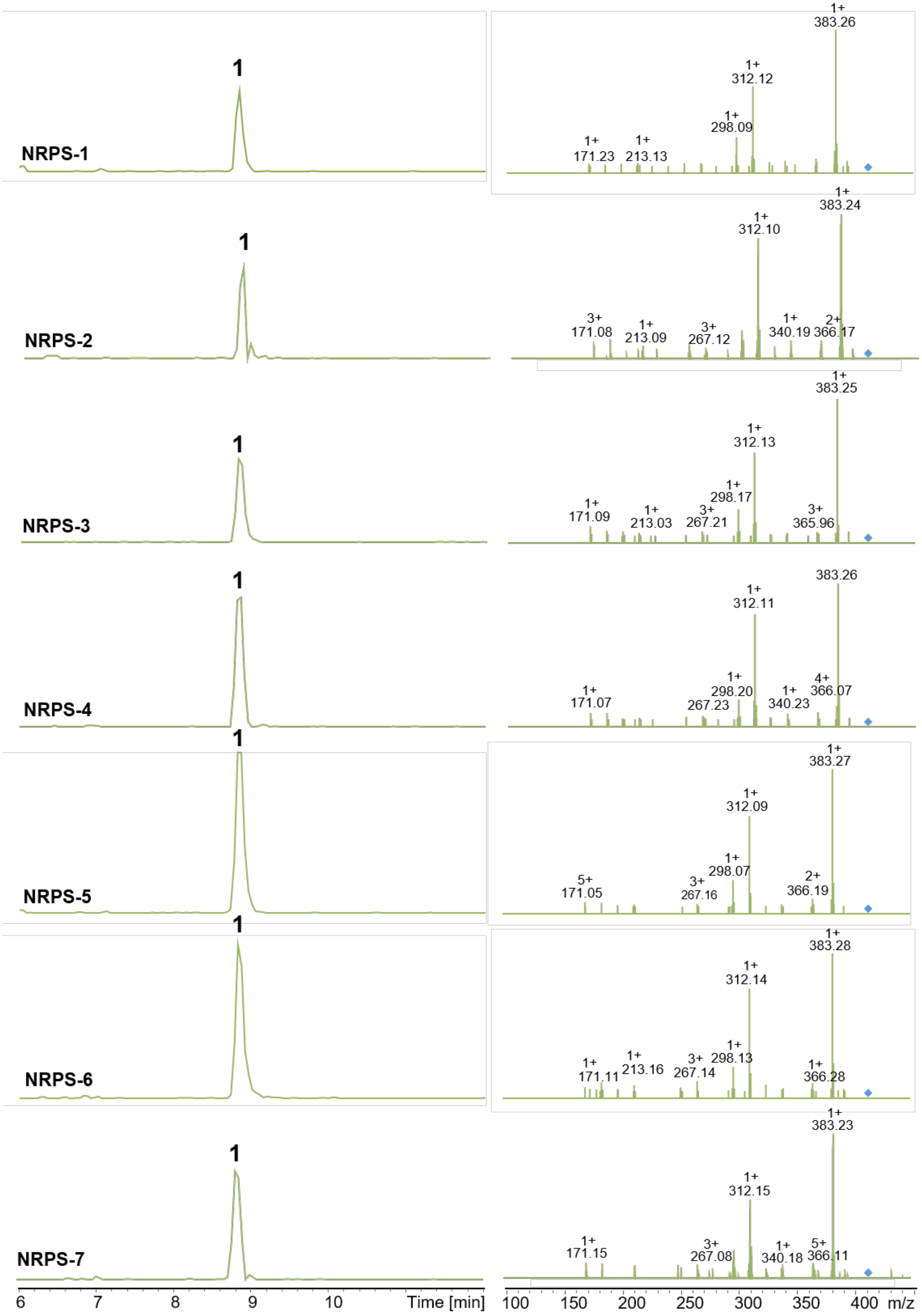
1
NRPS-1
1+ 383.26
1+ 312.12
1+ 298.09
1+ 171.23
1+ 213.13
NRPS-2
1+ 383.24
1+ 312.10
3+ 171.08
1+ 213.09
3+ 267.12
1+ 340.19
2+ 366.17
NRPS-3
1+ 383.25
1+ 312.13
1+ 298.17
1+ 171.09
1+ 213.03
3+ 267.21
3+ 365.96
NRPS-4
383.26
1+ 312.11
1+ 298.20
1+ 171.07
267.23
1+ 340.23
4+ 366.07
NRPS-5
1+ 383.27
1+ 312.09
1+ 298.07
5+ 171.05
3+ 267.16
2+ 366.19
NRPS-6
1+ 383.28
1+ 312.14
1+ 298.13
1+ 171.11
1+ 213.16
3+ 267.14
1+ 366.28
NRPS-7
1+ 383.23
1+ 312.15
1+ 171.15
3+ 267.08
1+ 340.18
5+ 366.11
6 7 8 9 10 Time [min]
100 150 200 250 300 350 400 m/z

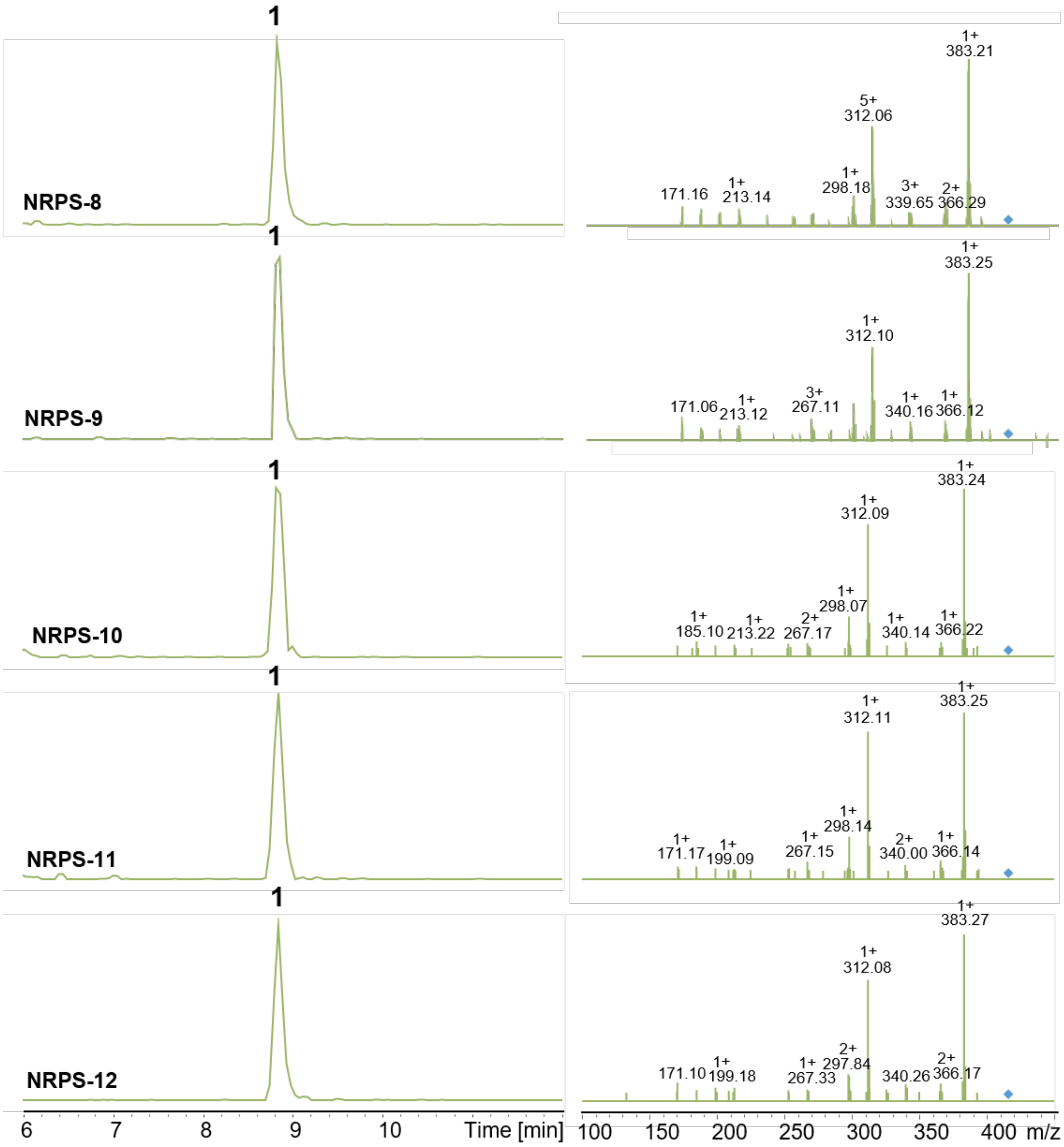

Figure S3. HPLC/MS data (Figure 2) of compound 1 produced in *E. coli* DH10B::*mtaA*. EIC/MS^2 of 1 (m/z $[M+H]^+$ = 411.29) produced by NRPS-1 to -12.

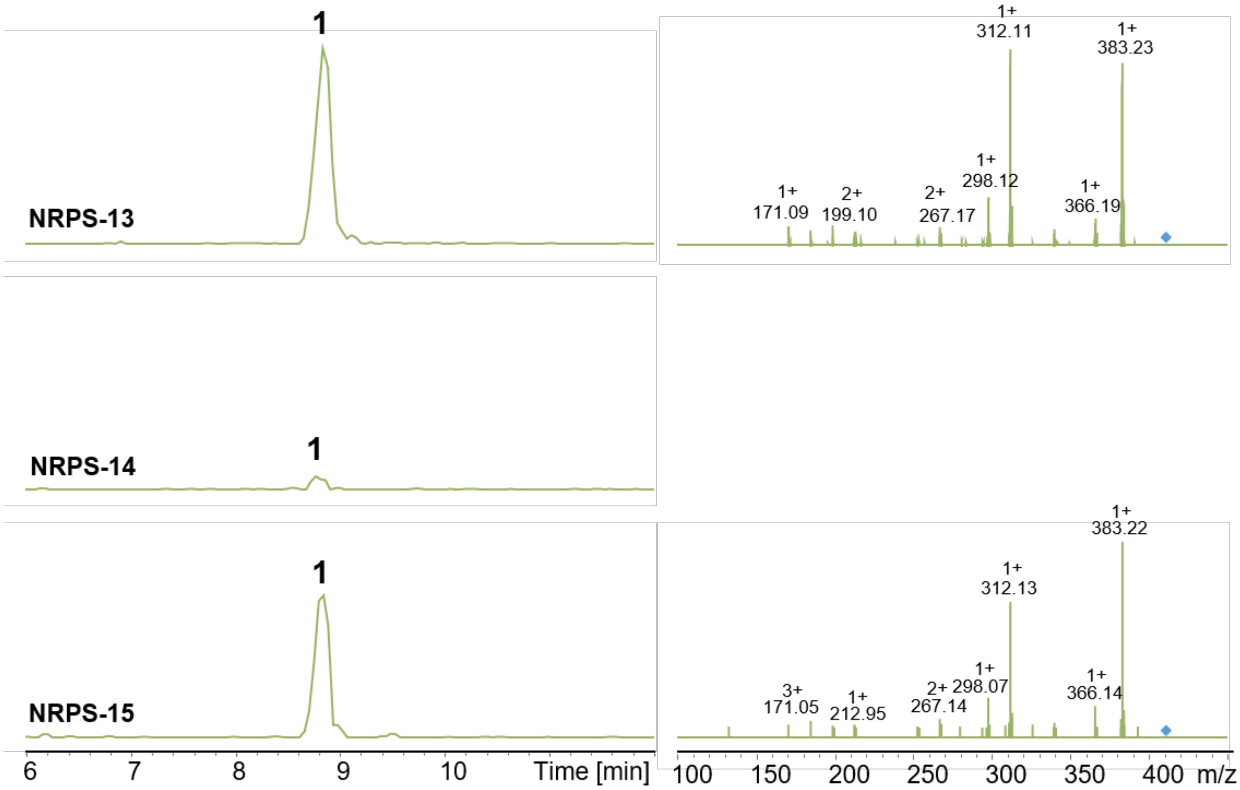

Figure S4. HPLC/MS data (Figure 3) of compounds 1 produced in *E. coli* DH10B::*taA*. EIC/MS^2 of 1 (m/z $[M+H]^+$ = 411.29) produced by NRPS-13 to -15.

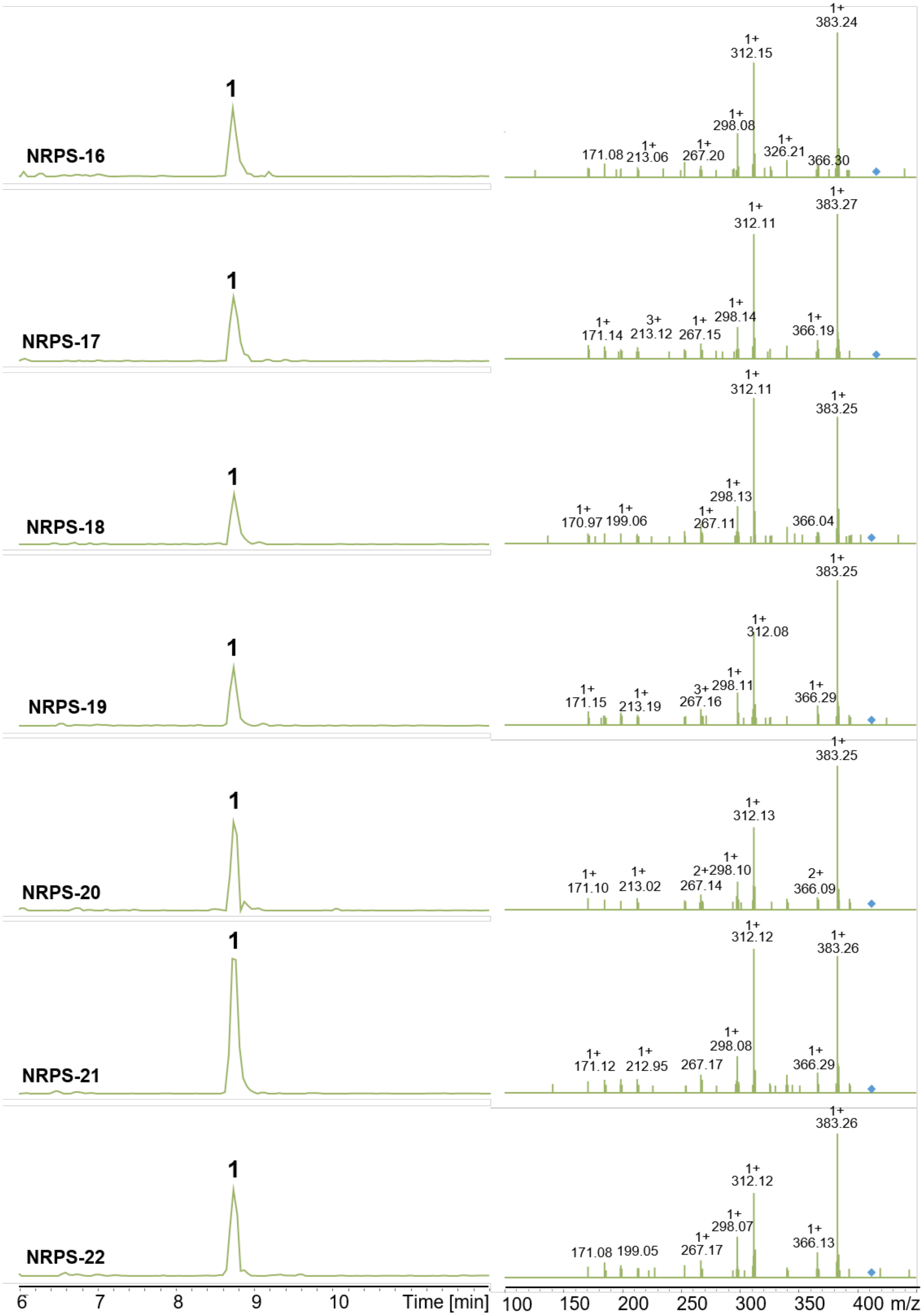

NRPS-16
NRPS-17
NRPS-18
NRPS-19
NRPS-20
NRPS-21
NRPS-22
1
1+ 383.24
1+ 312.15
1+ 298.08
171.08
1+ 213.06
1+ 267.20
1+ 326.21
366.30
1+ 383.27
1+ 312.11
1+ 171.14
3+ 213.12
1+ 267.15
1+ 298.14
1+ 366.19
1+ 312.11
1+ 383.25
1+ 298.13
1+ 170.97
1+ 199.06
1+ 267.11
366.04
1+ 383.25
1+ 312.08
1+ 171.15
1+ 213.19
3+ 267.16
1+ 298.11
1+ 366.29
1+ 383.25
1+ 312.13
1+ 171.10
1+ 213.02
2+ 267.14
1+ 298.10
2+ 366.09
1+ 312.12
1+ 383.26
1+ 171.12
1+ 212.95
1+ 298.08
267.17
1+ 366.29
1+ 383.26
1+ 312.12
1+ 298.07
171.08
199.05
1+ 267.17
1+ 366.13
6
7
8
9
10
Time [min]
100
150
200
250
300
350
400
m/z

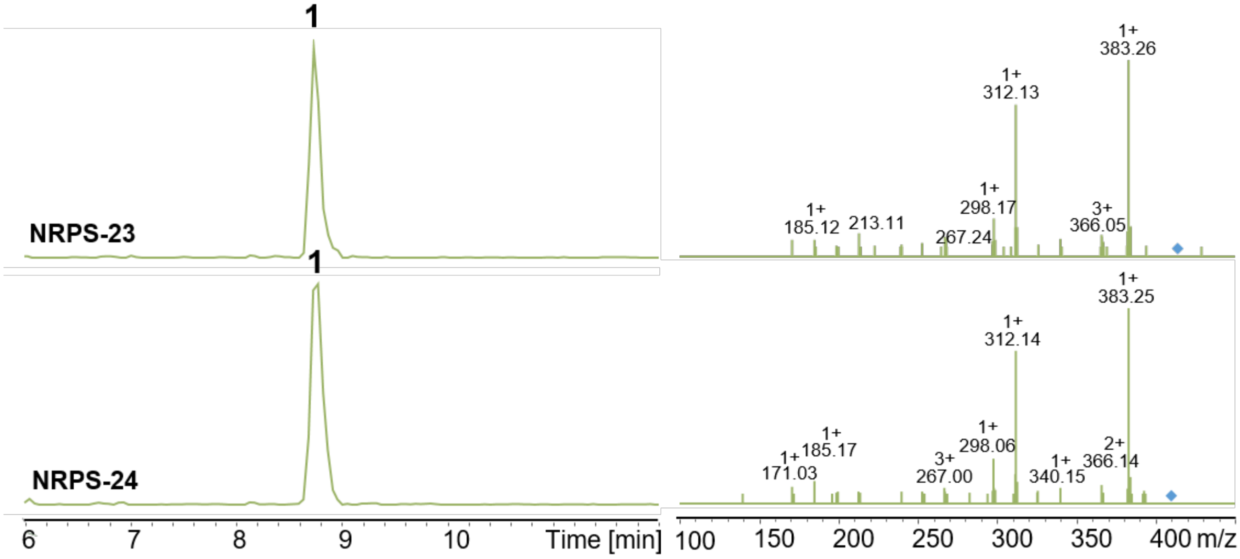

Figure S5. HPLC/MS data (Figure 4) of compound 1 produced in *E. coli* DH10B::*mtaA*. EIC/MS^2 of 1 (m/z $[M+H]^+$ = 411.29) produced by NRPS-16 to -24.

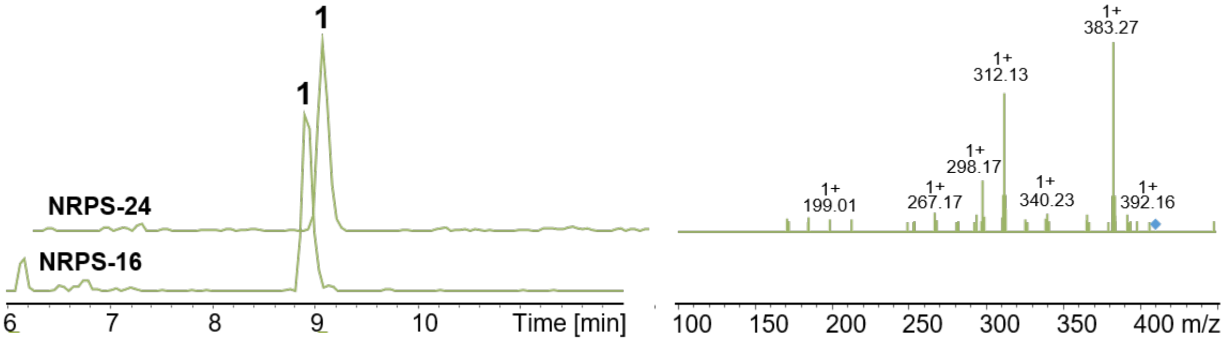

Figure S6. HPLC/MS data (Figure 5) of compound 1 produced in *E. coli* DH10B::*mtaA*. EIC/MS2 of 1 (m/z [M+H]+ = 411.29) produced by NRPS-24. EIC of 1 produced by NRPS-16**.**

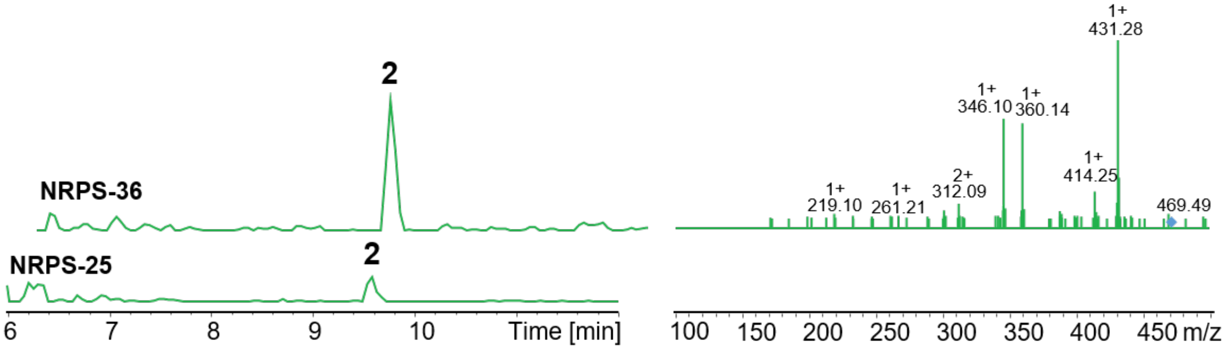

Figure S7. HPLC/MS data (Figure 5) of compound 2 produced in E. coli DH10B::mtaA. EIC/MS2 of 2 (m/z [M+H]+ = 459.30) produced by NRPS-36. EIC of 1 produced by NRPS-25.

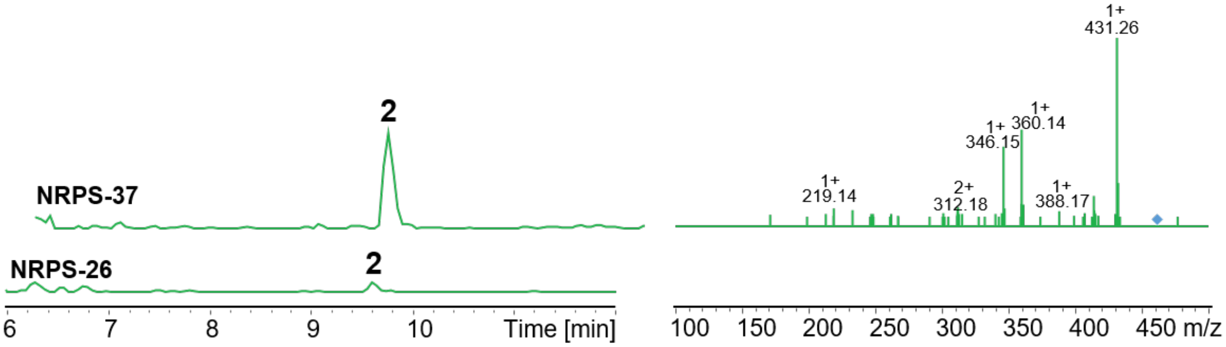

Figure S8. HPLC/MS data (Figure 5) of compound 2 produced in *E. coli* DH10B::*mtaA*. EIC/MS^2 of 2 (m/z [M+H]$^+$ = 459.30) produced by NRPS-37. EIC of 1 produced by NRPS-26.

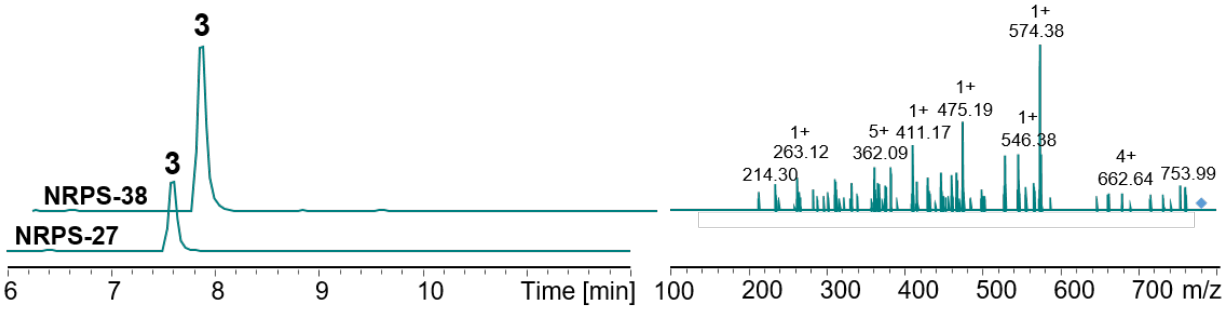

Figure S9. HPLC/MS data (Figure 5) of compound 3 produced in *E. coli* DH10B::*mtaA*. EIC/MS^2 of 3 (m/z [M+H]$^+$ = 778,45) produced by NRPS-38. EIC of 3 produced by NRPS-27.

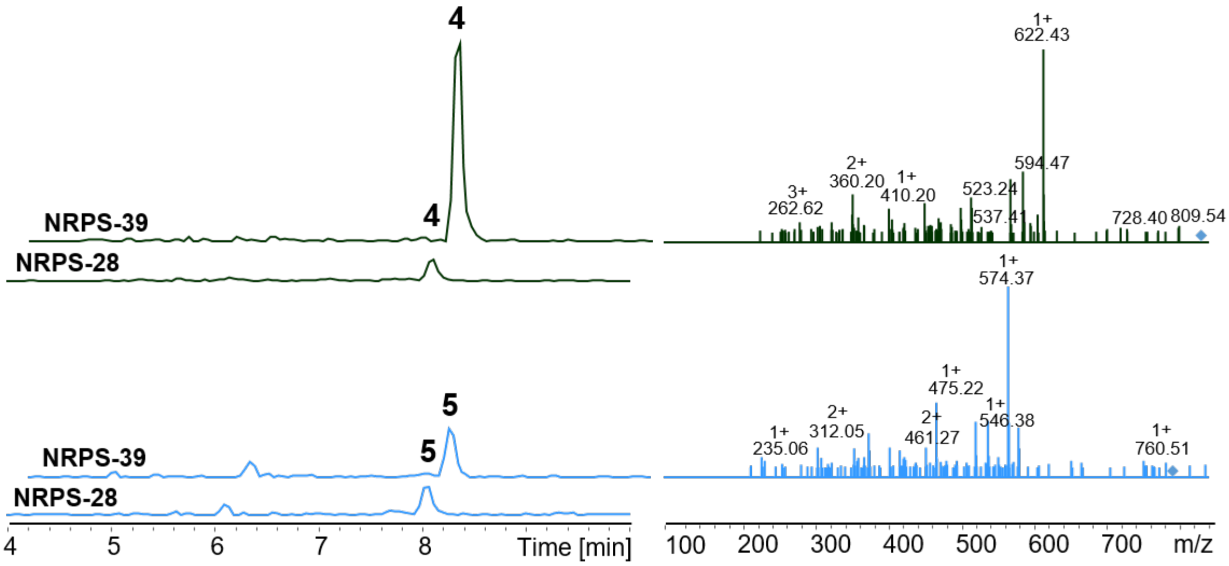

Figure S10. HPLC/MS data (Figure 5) of compounds 4 and 5 produced in *E. coli* DH10B::*mtaA*. EIC/MS^2 of 4 (m/z $[M+H]^+$ = 826.45) and 5 (m/z $[M+H]^+$ = 792.47) produced by NRPS-38. EIC of 4 and 5 produced by NRPS-28.

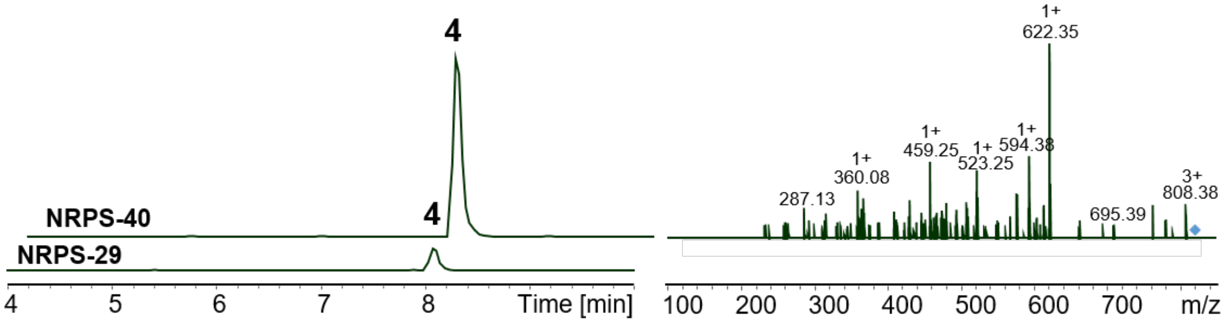

Figure S11. HPLC/MS data (Figure 5) of compound 4 produced in *E. coli* DH10B::*mtaA*. EIC/MS^2 of 4 (m/z $[M+H]^+$ = 826.45) produced by NRPS-38. EIC of 4 produced by NRPS-28.

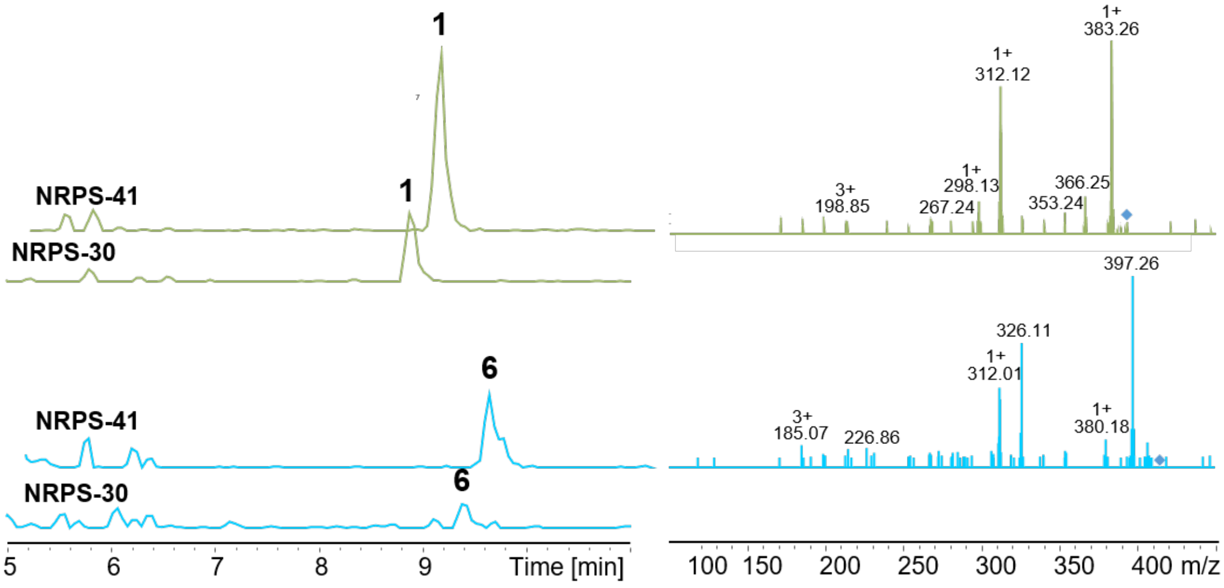

Figure S12. HPLC/MS data (Figure 5) of compounds 1 and 6 produced in E. coli DH10B::mtaA. EIC/MS^2 of 1 (m/z $[M+H]^+$ = 411.29) and 6 (m/z $[M+H]^+$ = 425.31) produced by NRPS-41. EIC of 1 and 6 produced by NRPS-30.

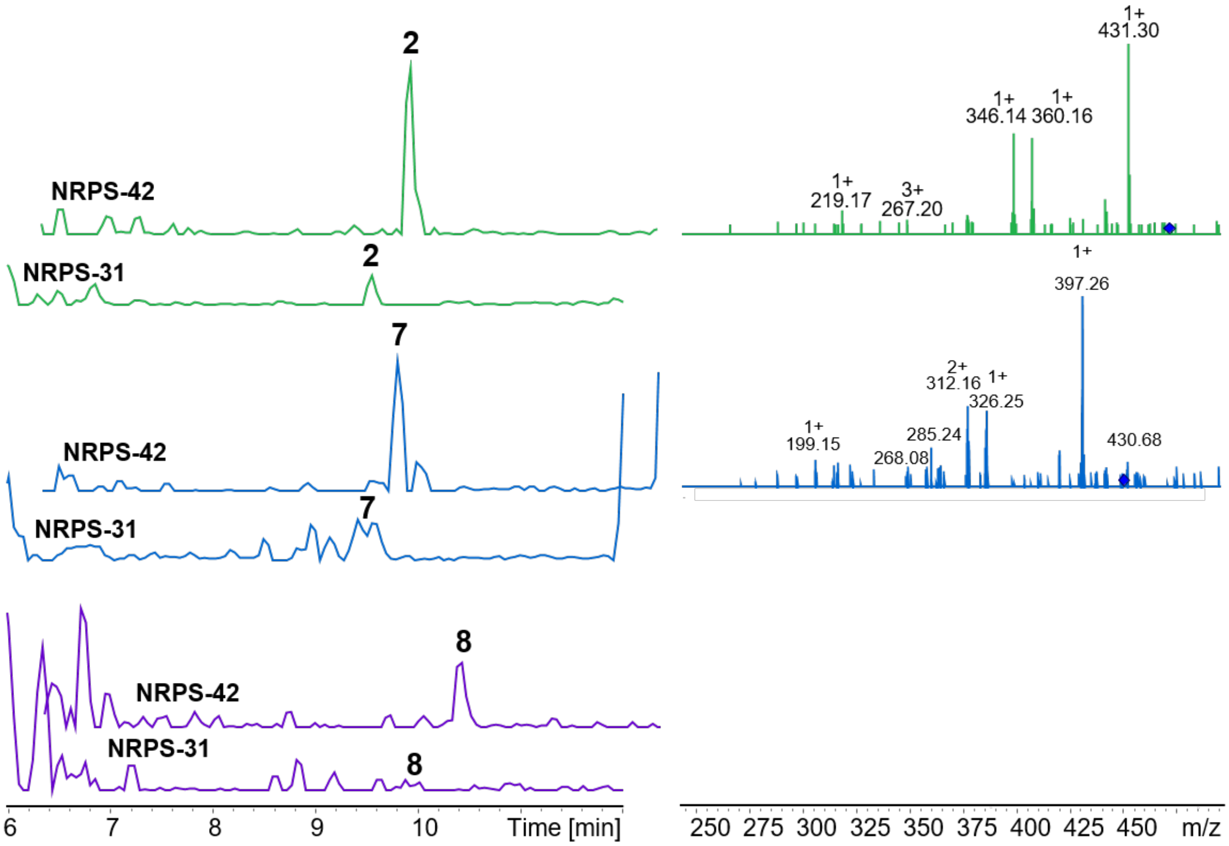

Figure S13. HPLC/MS data (Figure 5) of compounds 2, 7 and 8 produced in *E. coli* DH10B::*mtaA*. EIC/MS^2 of 2 (m/z $[M+H]^+$ = 459.30), 7 (m/z $[M+H]^+$ = 425.31) and 8 m/z $[M+H]^+$ = 472.31) produced by NRPS-42. EIC of 2, 7 and 8 produced by NRPS-31.

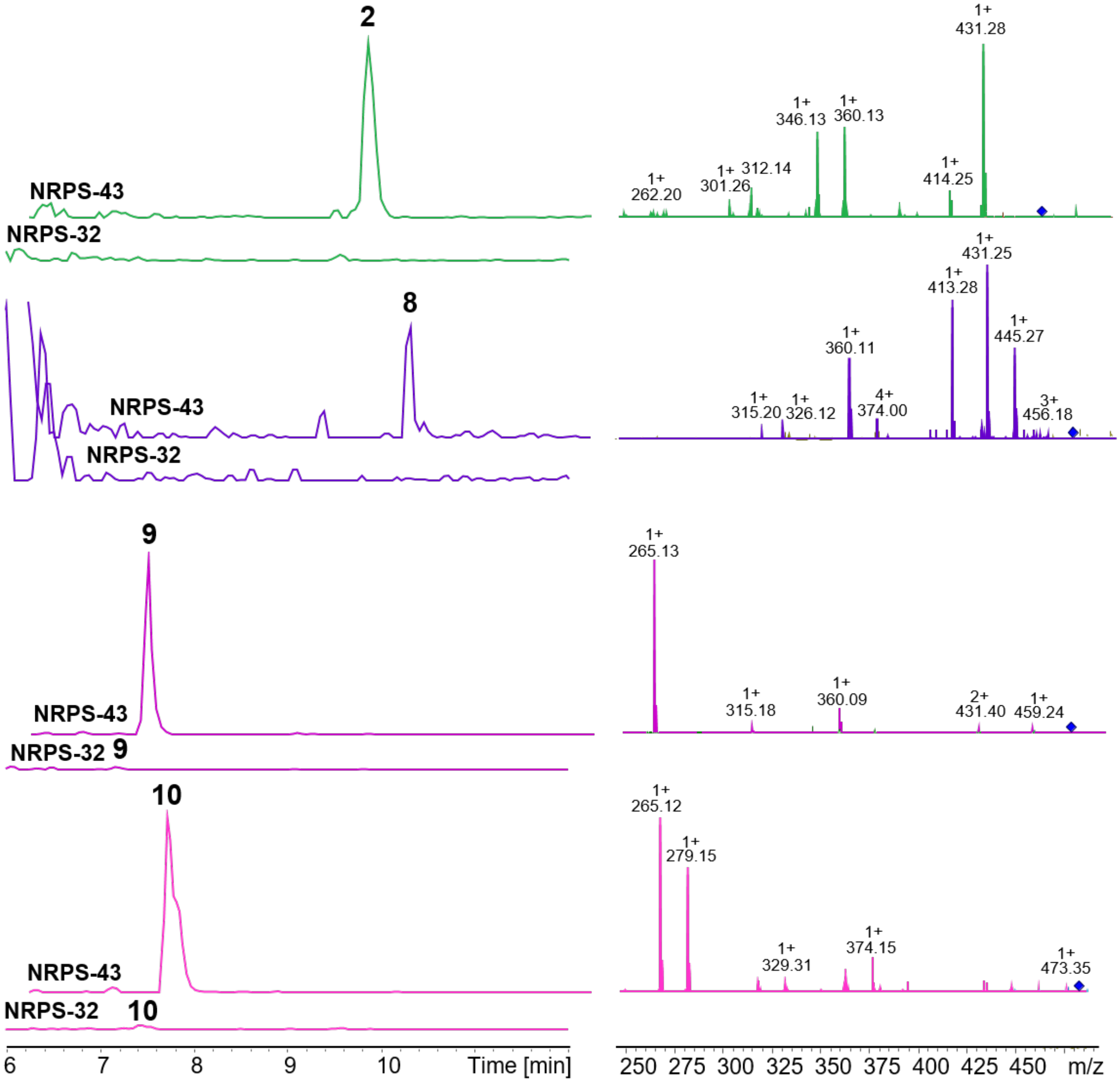

Figure S14. HPLC/MS data (Figure 5) of compounds 2, 8, 9 and 10 produced in *E. coli* DH10B::*mtaA*. EIC/MS^2 of 2 (m/z $[M+H]^+$ = 459.30), 8 (m/z $[M+H]^+$ = 472.31), 9 (m/z $[M+H]^+$ = 476.62) and 10 m/z $[M+H]^+$ = 490.65) produced by NRPS-43. EIC of 2, 8, 9 and 10 produced by NRPS-32.

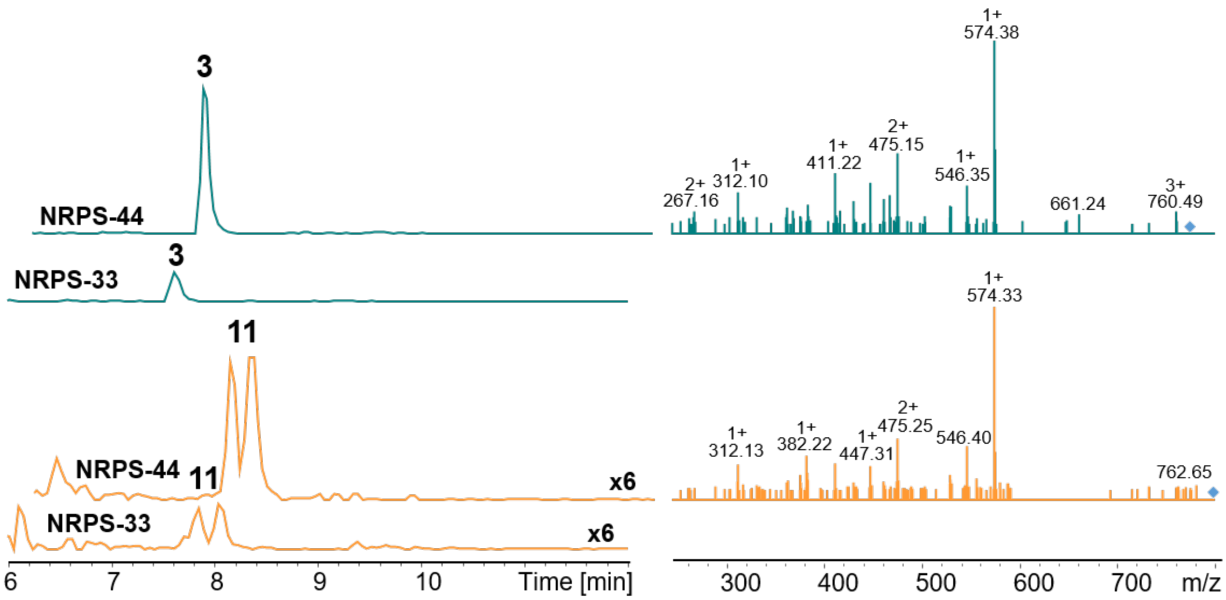

Figure S15. HPLC/MS data (Figure 5) of compounds 3 and 11 produced in E. coli DH10B::mtaA. EIC/MS^2 of 3 (m/z [M+H]$^+$ = 778.45) and 11 (m/z [M+H]$^+$ = 792.47) produced by NRPS-44. EIC of 3 and 11 produced by NRPS-33.

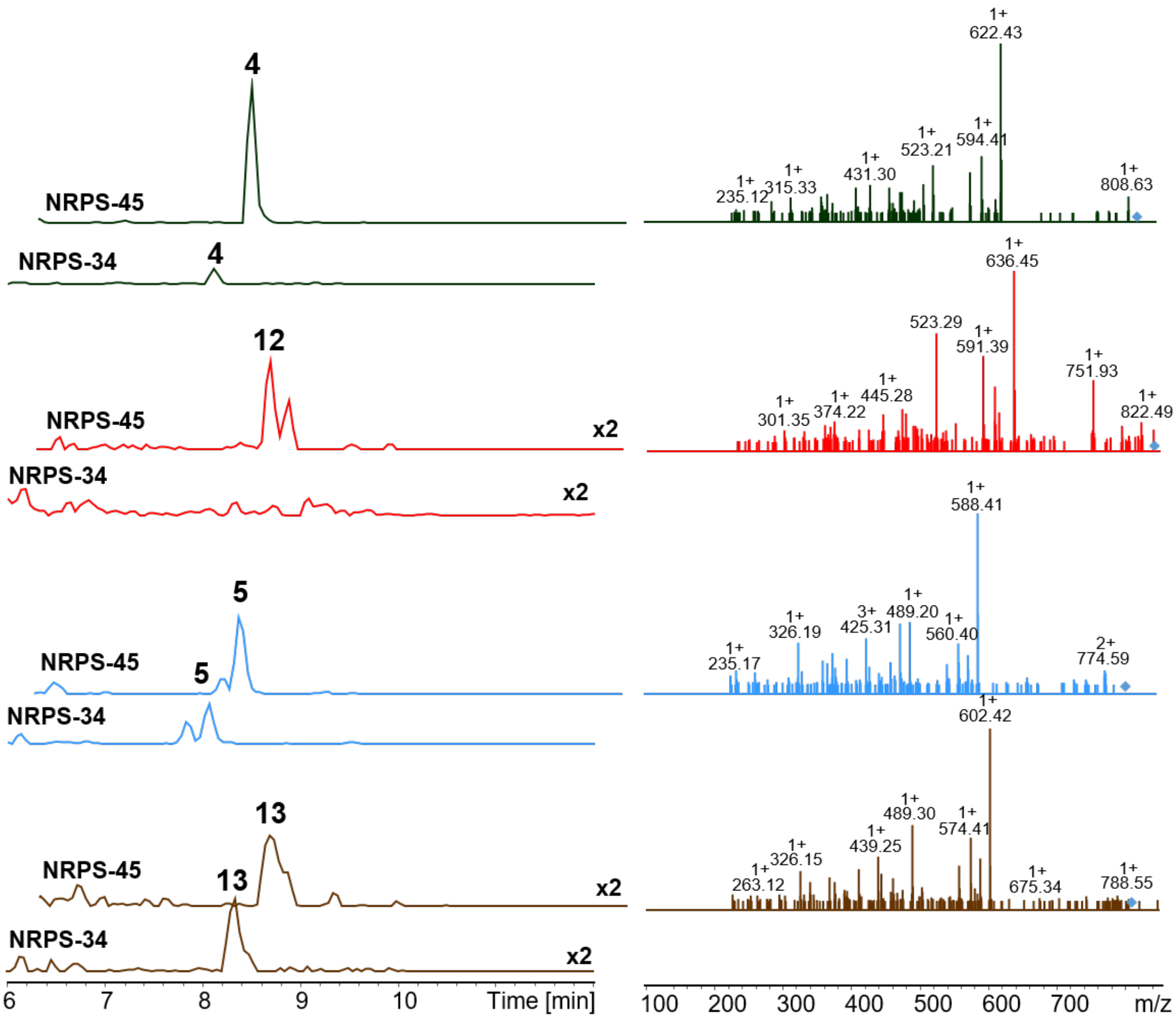

Figure S16. **HPLC/MS data (Figure 5) of compounds 4, 12, 5 and 13 produced in *E. coli* DH10B::*mtaA*.** EIC/MS^2 of 4 (m/z $[M+H]^+$ = 826.45), 12 (m/z $[M+H]^+$ = 840.47), 5 (m/z $[M+H]^+$ = 792.47) and 13 m/z $[M+H]^+$ = 806.48) produced by NRPS-45. EIC of 2, 12, 5 and 13 produced by NRPS-34.

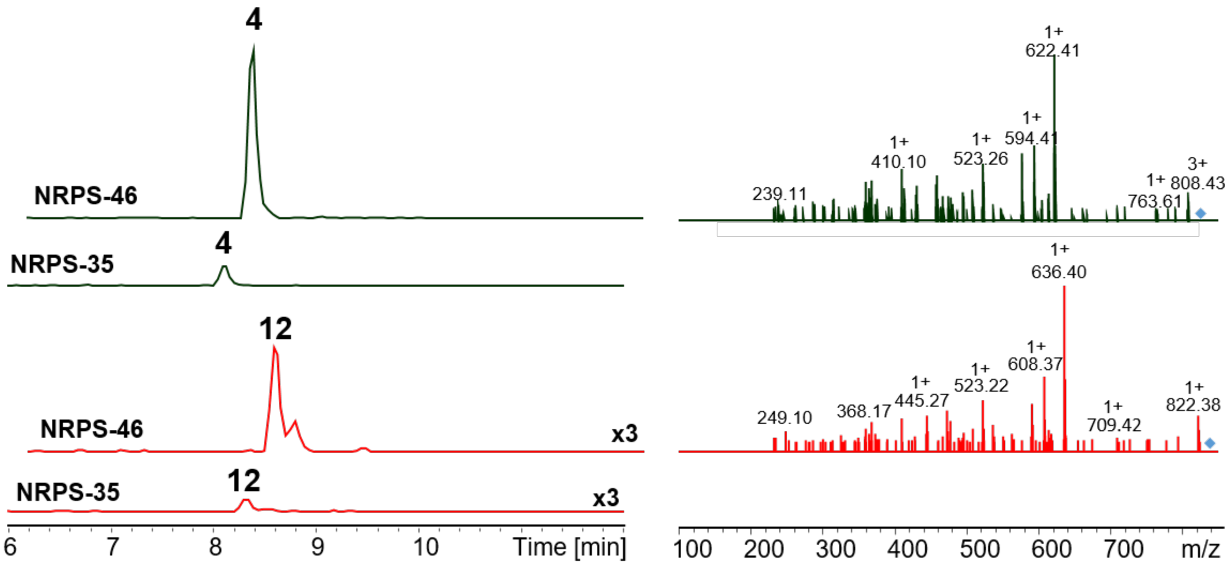

Figure S17. **HPLC/MS data (Figure 5) of compounds 4 and 12 produced in *E. coli* DH10B::*mtaA*.** EIC/MS^2 of 4 (m/z [M+H]$^+$ = 826.45) and 12 (m/z [M+H]$^+$ = 840.47) produced by NRPS-46. EIC of 4 and 12 produced by NRPS-35.

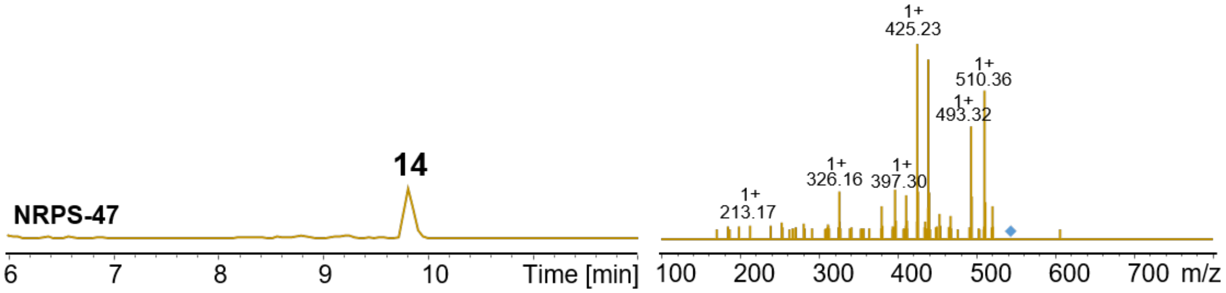

Figure S18. HPLC/MS data (Figure 6) of compound 14 produced in *E. coli* DH10B::*mtaA*. EIC/MS^2 of 14 (m/z [M+H]$^+$ = 538.40) produced by NRPS-47.

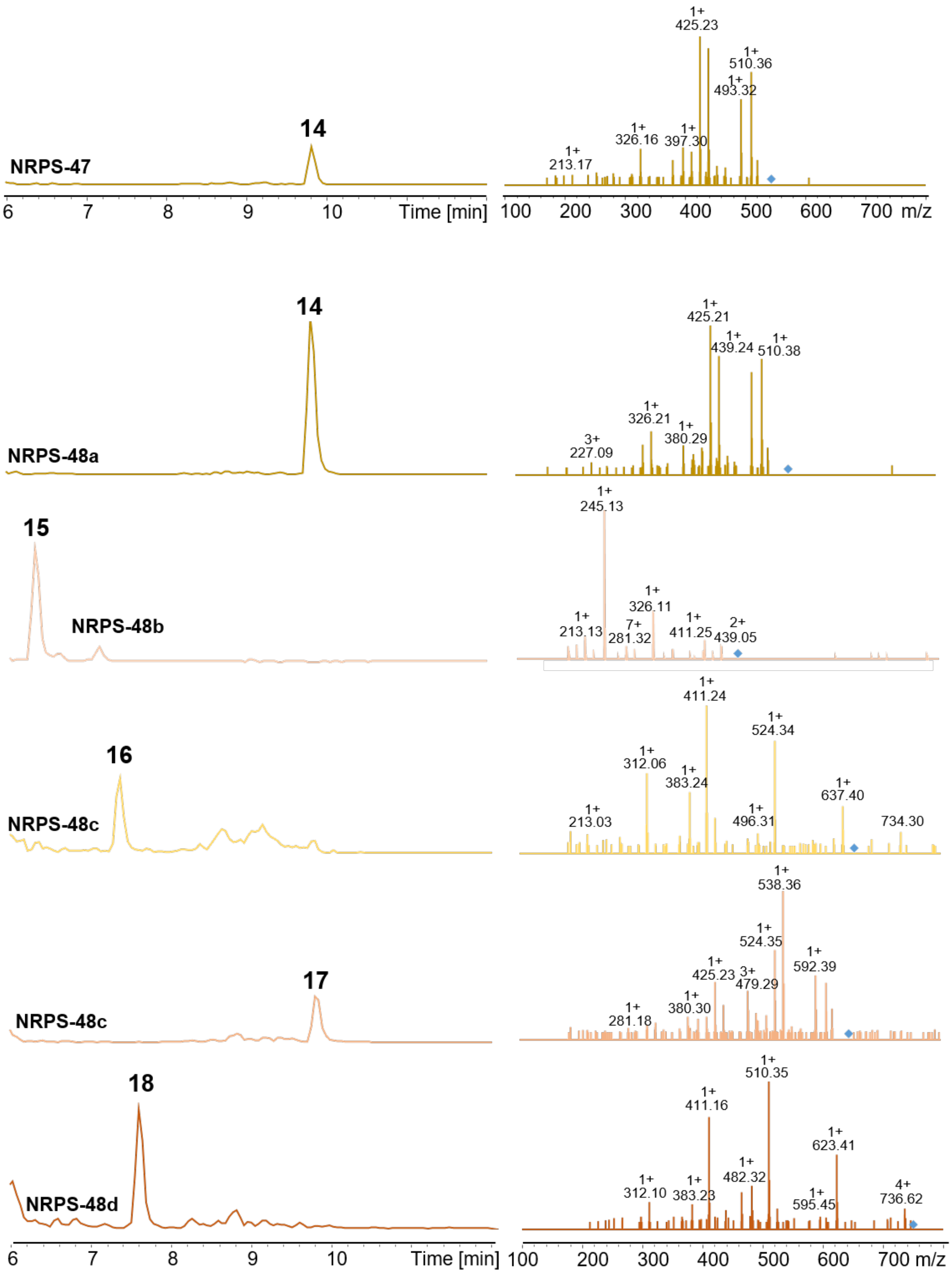
NRPS-47
14
1+ 425.23
1+ 510.36
1+ 493.32
1+ 326.16
1+ 397.30
1+ 213.17
6 7 8 9 10 Time [min]
100 200 300 400 500 600 700 m/z
NRPS-48a
14
1+ 425.21
1+ 439.24
1+ 510.38
1+ 326.21
1+ 380.29
3+ 227.09
NRPS-48b
15
1+ 245.13
1+ 326.11
1+ 213.13
7+ 281.32
1+ 411.25
2+ 439.05
NRPS-48c
16
1+ 411.24
1+ 524.34
1+ 312.06
1+ 383.24
1+ 637.40
1+ 213.03
1+ 496.31
734.30
NRPS-48c
17
1+ 538.36
1+ 524.35
1+ 425.23
3+ 479.29
1+ 592.39
1+ 380.30
1+ 281.18
NRPS-48d
18
1+ 510.35
1+ 411.16
1+ 623.41
1+ 482.32
1+ 312.10
1+ 383.23
1+ 595.45
4+ 736.62
6 7 8 9 10 Time [min]
100 200 300 400 500 600 700 m/z

Figure S19. HPLC/MS data (Figure 5) of compounds 14, 15, 16, 17 and 18 produced in *E. coli* DH10B::*mtaA*. EIC/MS^2 of 14 (m/z $[M+H]^+$ = 538.40) produced by NRPS-47 and -48a. EIC/MS^2 of 15 (m/z $[M+H]^+$ = 470.35) produced by NRPS-47. EIC/MS^2 of 16 (m/z $[M+H]^+$ = 655.47) produced by NRPS-48c. EIC/MS^2 of 17 (m/z $[M+7]^+$ = 637.46) produced by NRPS-48c. EIC/MS^2 of 18 m/z $[M+H]^+$ = 754.54) produced by NRPS-48d.

215

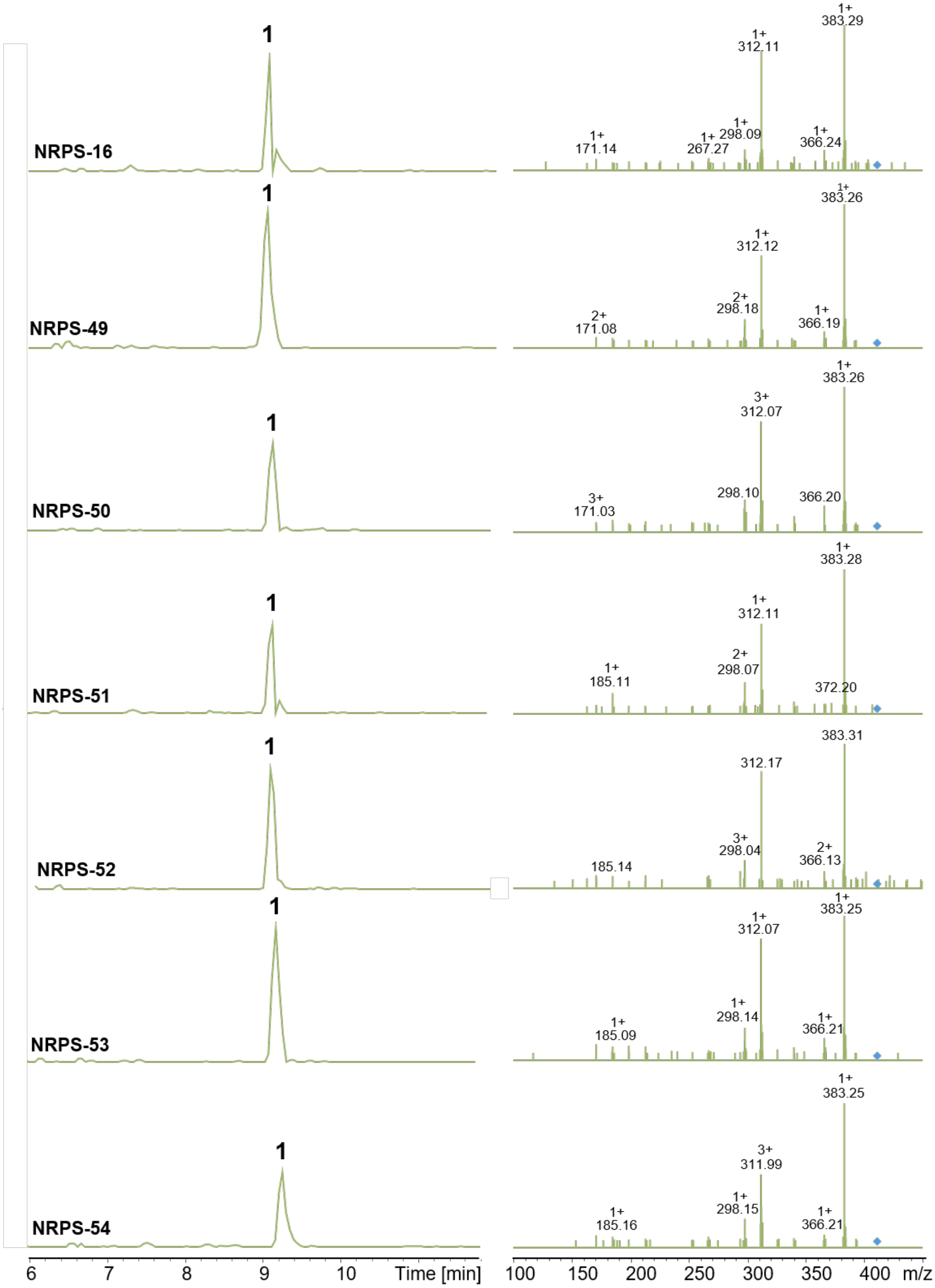

NRPS-16
NRPS-49
NRPS-50
NRPS-51
NRPS-52
NRPS-53
NRPS-54
1
1+ 383.29
1+ 312.11
1+ 298.09
1+ 267.27
1+ 171.14
1+ 366.24
1+ 383.26
1+ 312.12
2+ 298.18
2+ 171.08
1+ 366.19
1+ 383.26
3+ 312.07
298.10
3+ 171.03
366.20
1+ 383.28
1+ 312.11
2+ 298.07
1+ 185.11
372.20
383.31
312.17
3+ 298.04
185.14
2+ 366.13
1+ 383.25
1+ 312.07
1+ 298.14
1+ 185.09
1+ 366.21
1+ 383.25
3+ 311.99
1+ 298.15
1+ 185.16
1+ 366.21
6
7
8
9
10
Time [min]
100
150
200
250
300
350
400
m/z

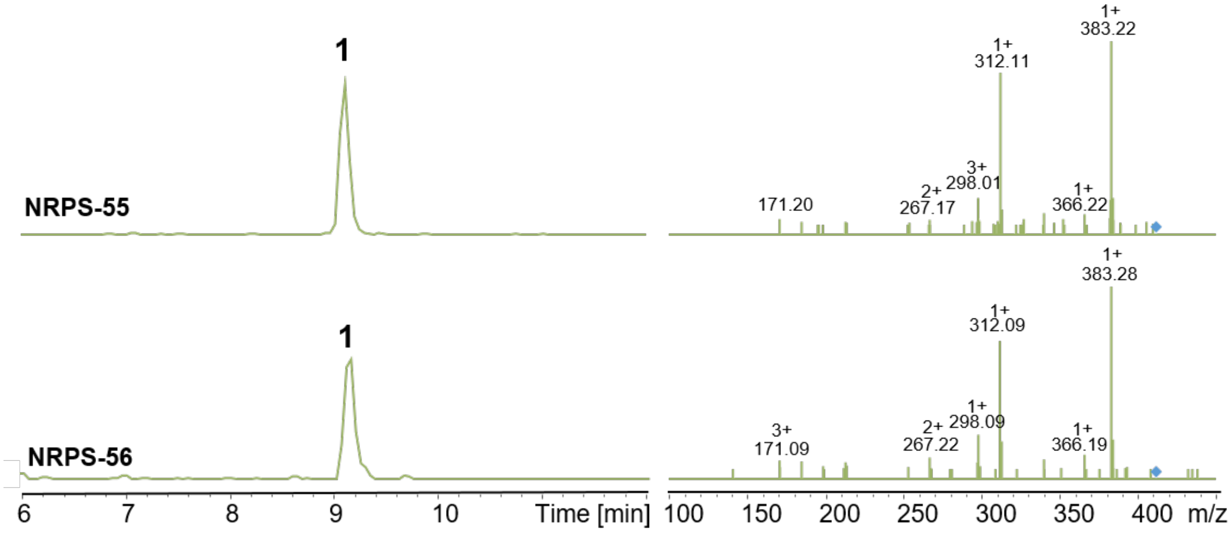

Figure S20. HPLC/MS data (Figure S25) of compound 1 produced in *E. coli* DH10B::*mtaA*. EIC/MS^2 of 1 (m/z $[M+H]^+$ = 411.29) produced by NRPS-16 and NRPS-49 to -56.

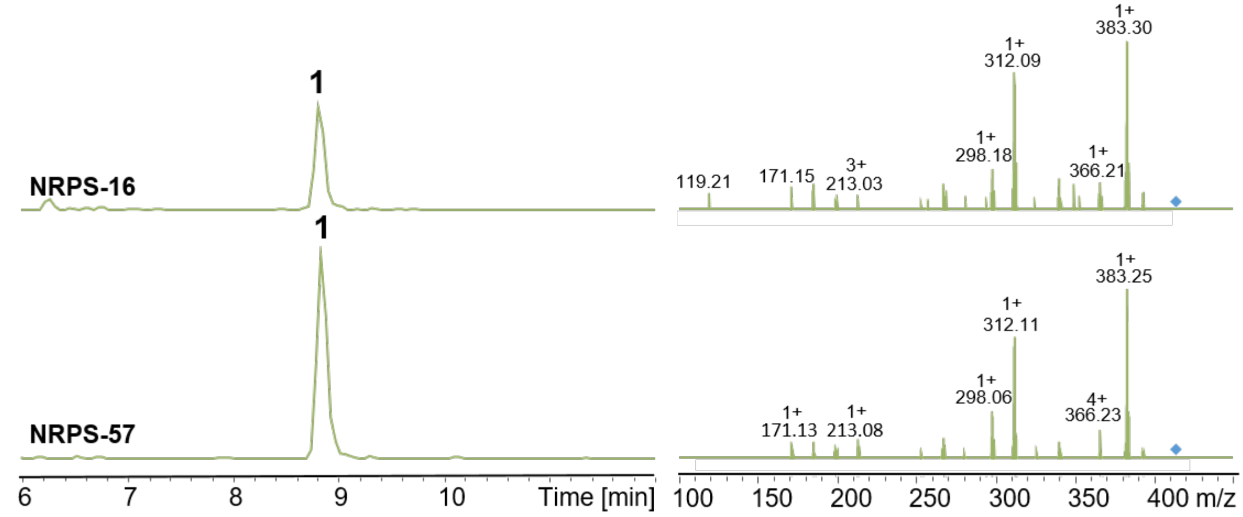

Figure S21. HPLC/MS data (Figure S26) of compound 1 produced in E. coli DH10B::mtaA. EIC/MS^2 of 1 (m/z $[M+H]^+$ = 411.29) produced by NRPS-16 and NRPS-57.

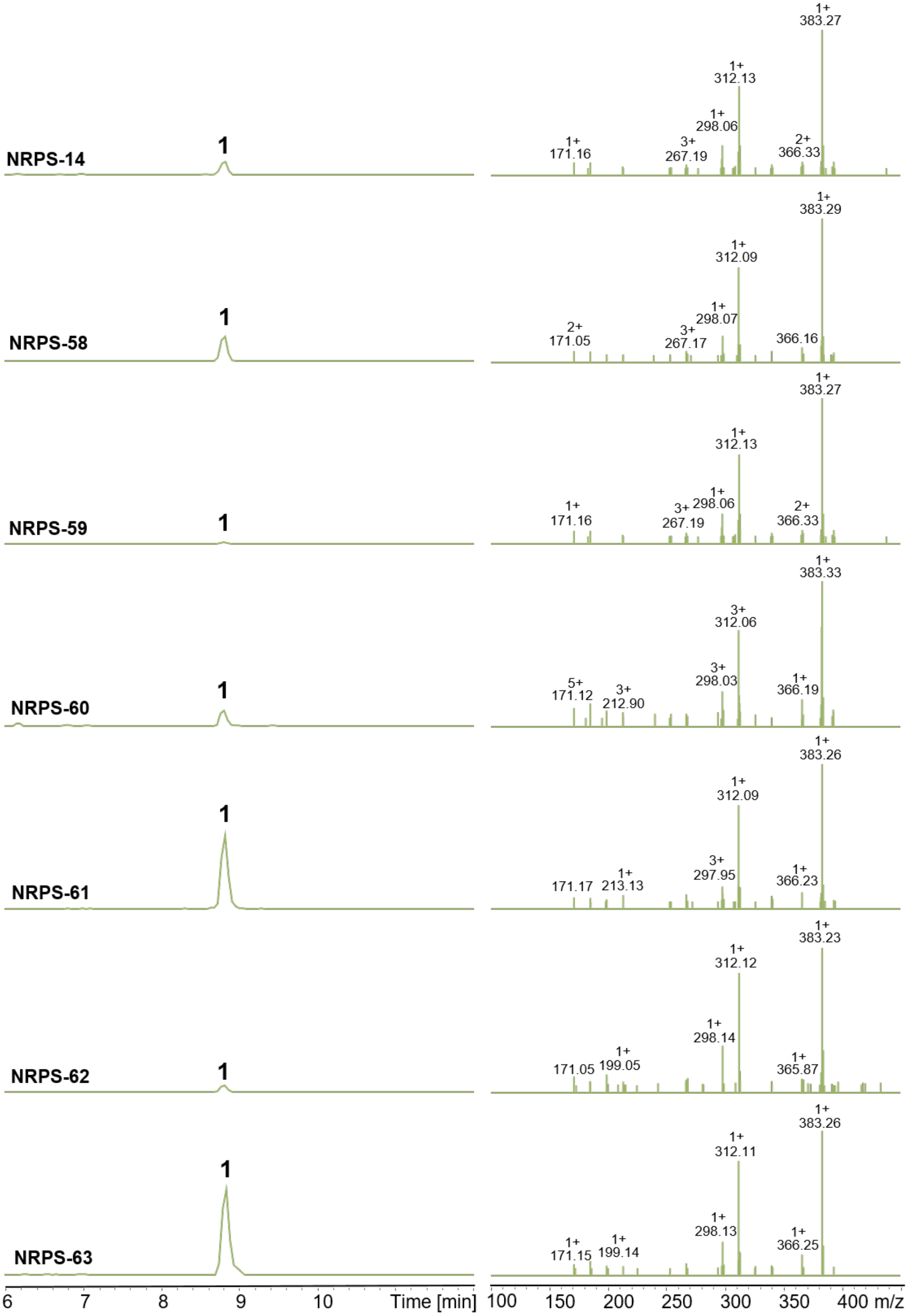

NRPS-14
1
1+ 171.16
3+ 267.19
1+ 298.06
1+ 312.13
2+ 366.33
1+ 383.27
NRPS-58
1
2+ 171.05
3+ 267.17
1+ 298.07
1+ 312.09
366.16
1+ 383.29
NRPS-59
1
1+ 171.16
3+ 267.19
1+ 298.06
1+ 312.13
2+ 366.33
1+ 383.27
NRPS-60
1
5+ 171.12
3+ 212.90
3+ 298.03
3+ 312.06
1+ 366.19
1+ 383.33
NRPS-61
1
171.17
1+ 213.13
3+ 297.95
1+ 312.09
1+ 366.23
1+ 383.26
NRPS-62
1
171.05
1+ 199.05
1+ 298.14
1+ 312.12
1+ 365.87
1+ 383.23
NRPS-63
1
1+ 171.15
1+ 199.14
1+ 298.13
1+ 312.11
1+ 366.25
1+ 383.26
6 7 8 9 10 Time [min]
100 150 200 250 300 350 400 m/z

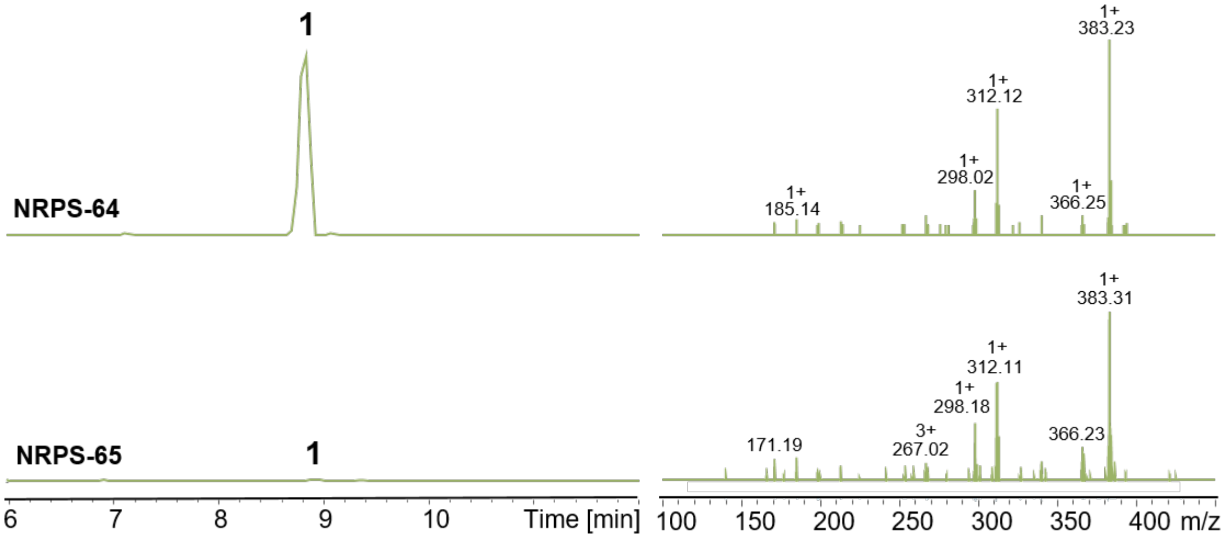

Figure S22. HPLC/MS data (Figure S27) of compound 1 produced in *E. coli* DH10B::*mtaA*. EIC/MS^2 of 1 (m/z $[M+H]^+$ = 411.29) produced by NRPS-14 and NRPS-58 to NRPS-65.

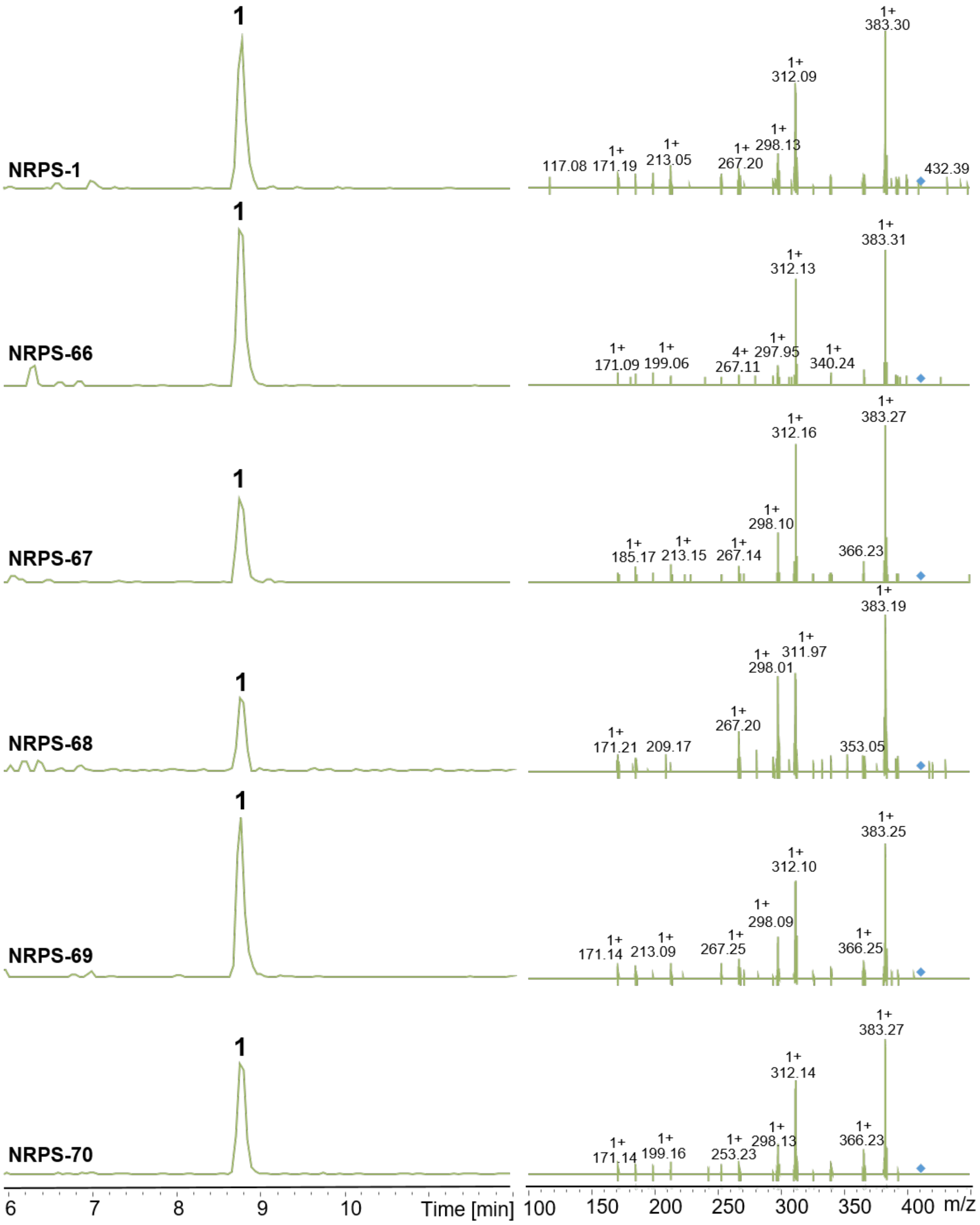
1
NRPS-1
NRPS-66
NRPS-67
NRPS-68
NRPS-69
NRPS-70
6
7
8
9
10
Time [min]
100
150
200
250
300
350
400
m/z
1+ 383.30
1+ 312.09
1+ 298.13
1+ 267.20
1+ 213.05
1+ 171.19
117.08
432.39
1+ 383.31
1+ 312.13
1+ 297.95
4+ 267.11
1+ 199.06
1+ 171.09
1+ 340.24
1+ 383.27
1+ 312.16
1+ 298.10
1+ 267.14
1+ 213.15
1+ 185.17
366.23
1+ 383.19
1+ 311.97
1+ 298.01
1+ 267.20
1+ 171.21
209.17
353.05
1+ 383.25
1+ 312.10
1+ 298.09
1+ 267.25
1+ 213.09
1+ 171.14
1+ 366.25
1+ 383.27
1+ 312.14
1+ 298.13
1+ 253.23
1+ 199.16
1+ 171.14
1+ 366.23

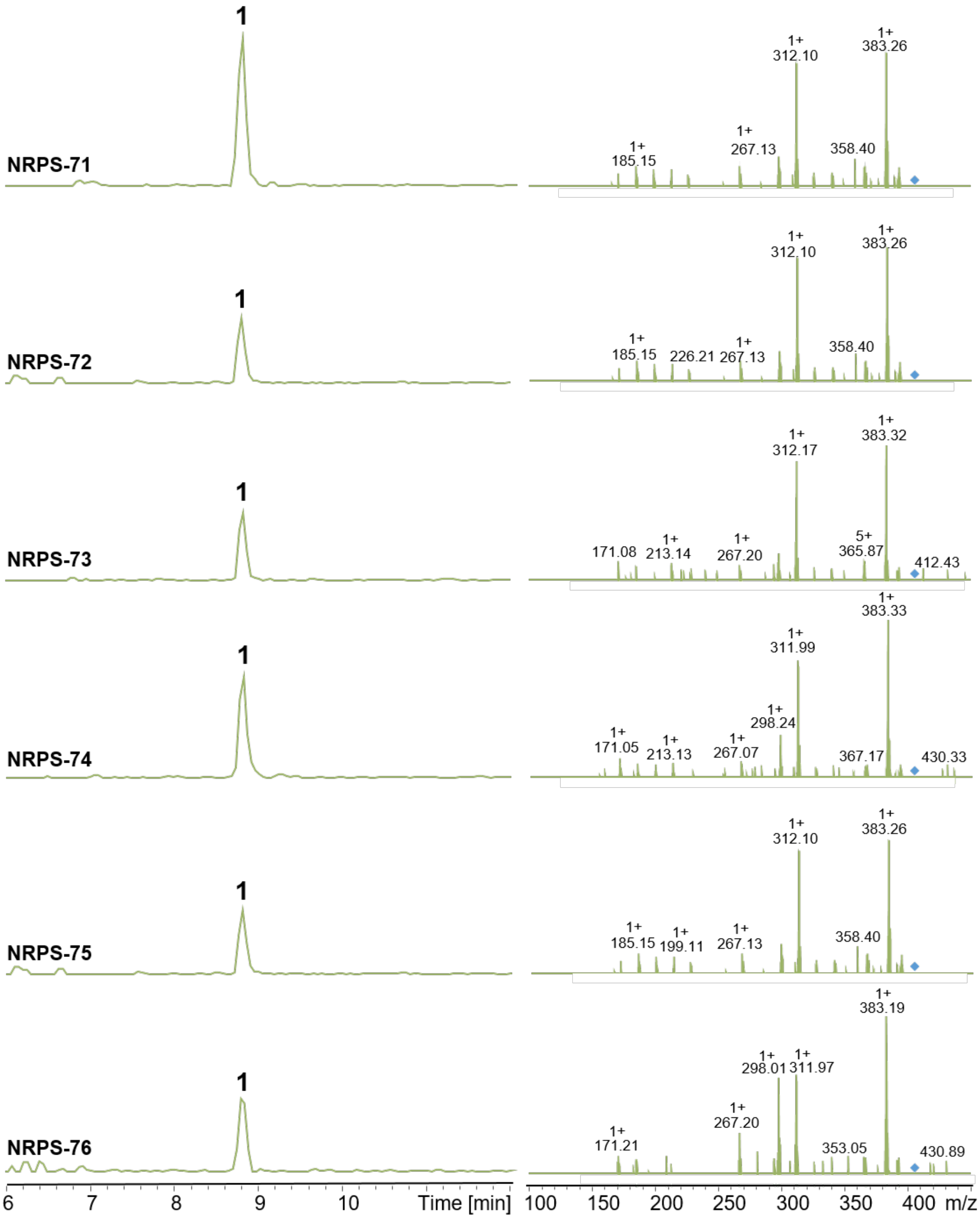
1
NRPS-71
1+
312.10
1+
383.26
1+
185.15
1+
267.13
358.40
1
NRPS-72
1+
312.10
1+
383.26
1+
185.15
226.21
1+
267.13
358.40
1
NRPS-73
1+
312.17
1+
383.32
171.08
1+
213.14
1+
267.20
5+
365.87
412.43
1
NRPS-74
1+
383.33
1+
311.99
1+
298.24
1+
171.05
1+
213.13
1+
267.07
367.17
430.33
1
NRPS-75
1+
312.10
1+
383.26
1+
185.15
1+
199.11
1+
267.13
358.40
1
NRPS-76
1+
383.19
1+
298.01
1+
311.97
1+
267.20
1+
171.21
353.05
430.89
6
7
8
9
10
Time [min]
100
150
200
250
300
350
400
m/z

231

232

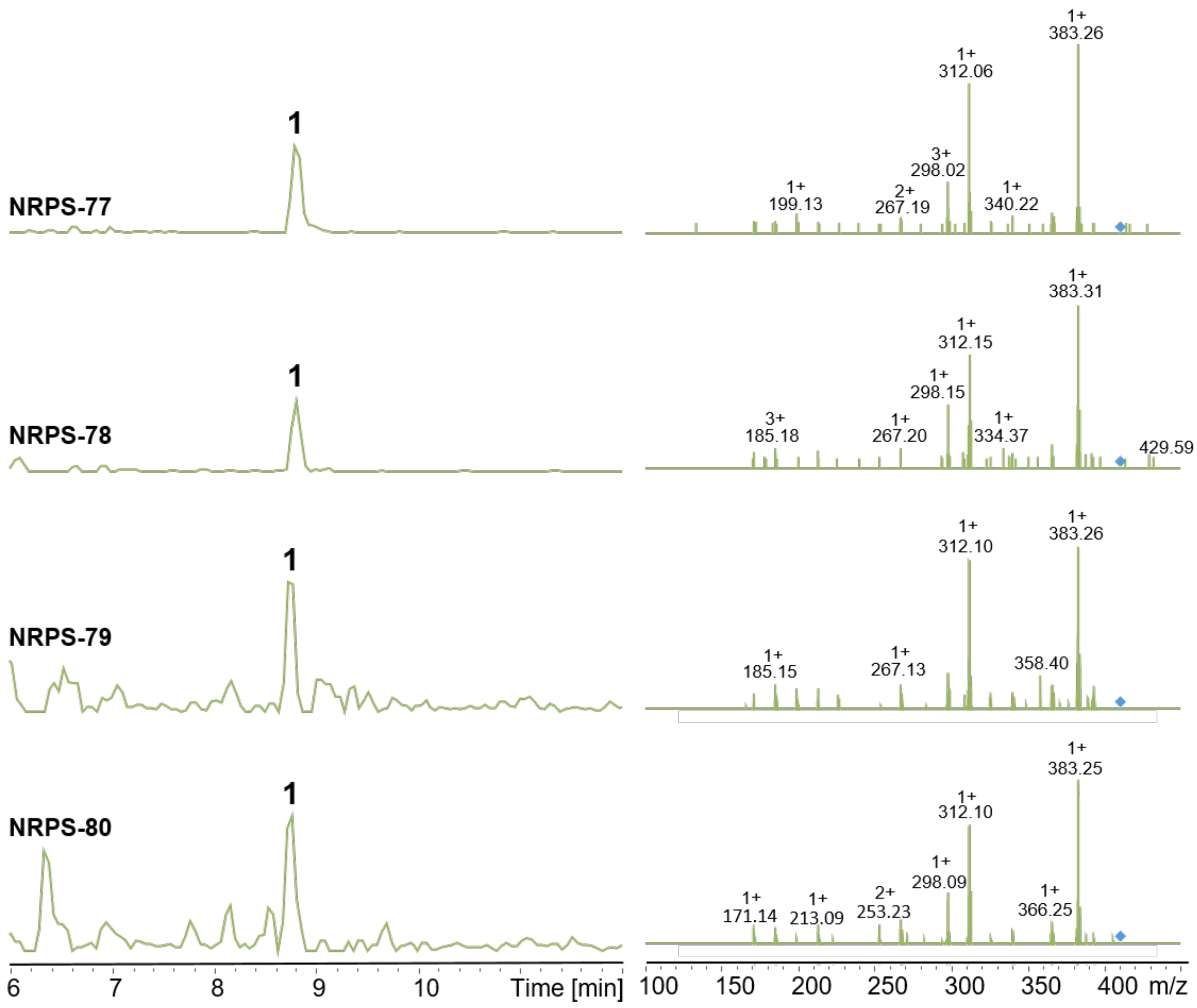

Figure S23. HPLC/MS data (Figure S28) of compound 1 produced in *E. coli* DH10B::*mtaA*. EIC/MS^2 of 1 (m/z $[M+H]^+$ = 411.29) produced by NRPS-1 and NRPS-66 to NRPS-80.

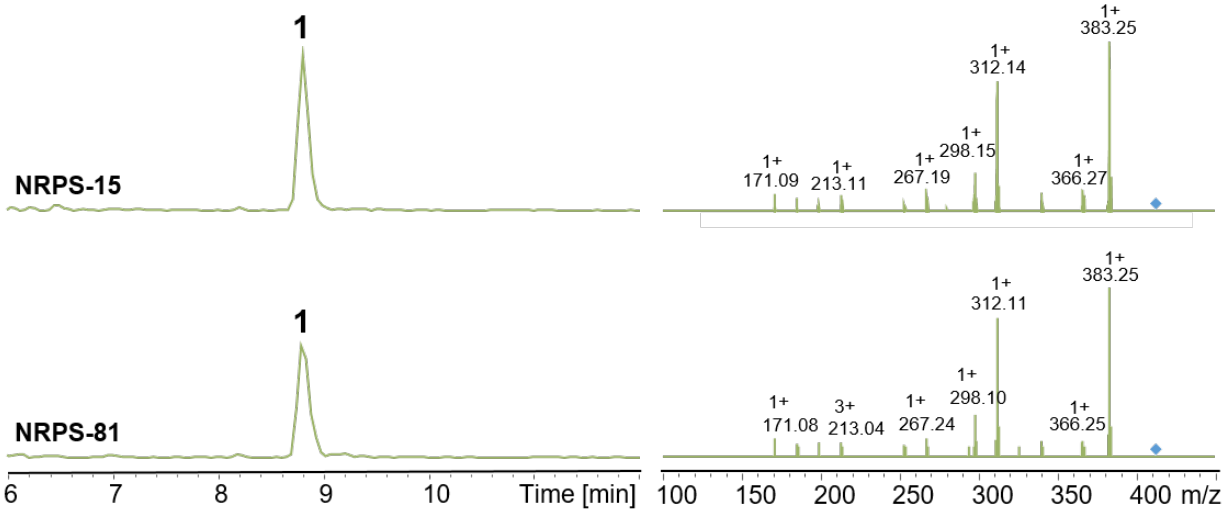

Figure S24. HPLC/MS data (Figure S29) of compound 1 produced in *E. coli* DH10B::*mtaA*. EIC/MS^2 of 1 (m/z $[M+H]^+$ = 411.29) produced by NRPS-15 and NRPS-81.

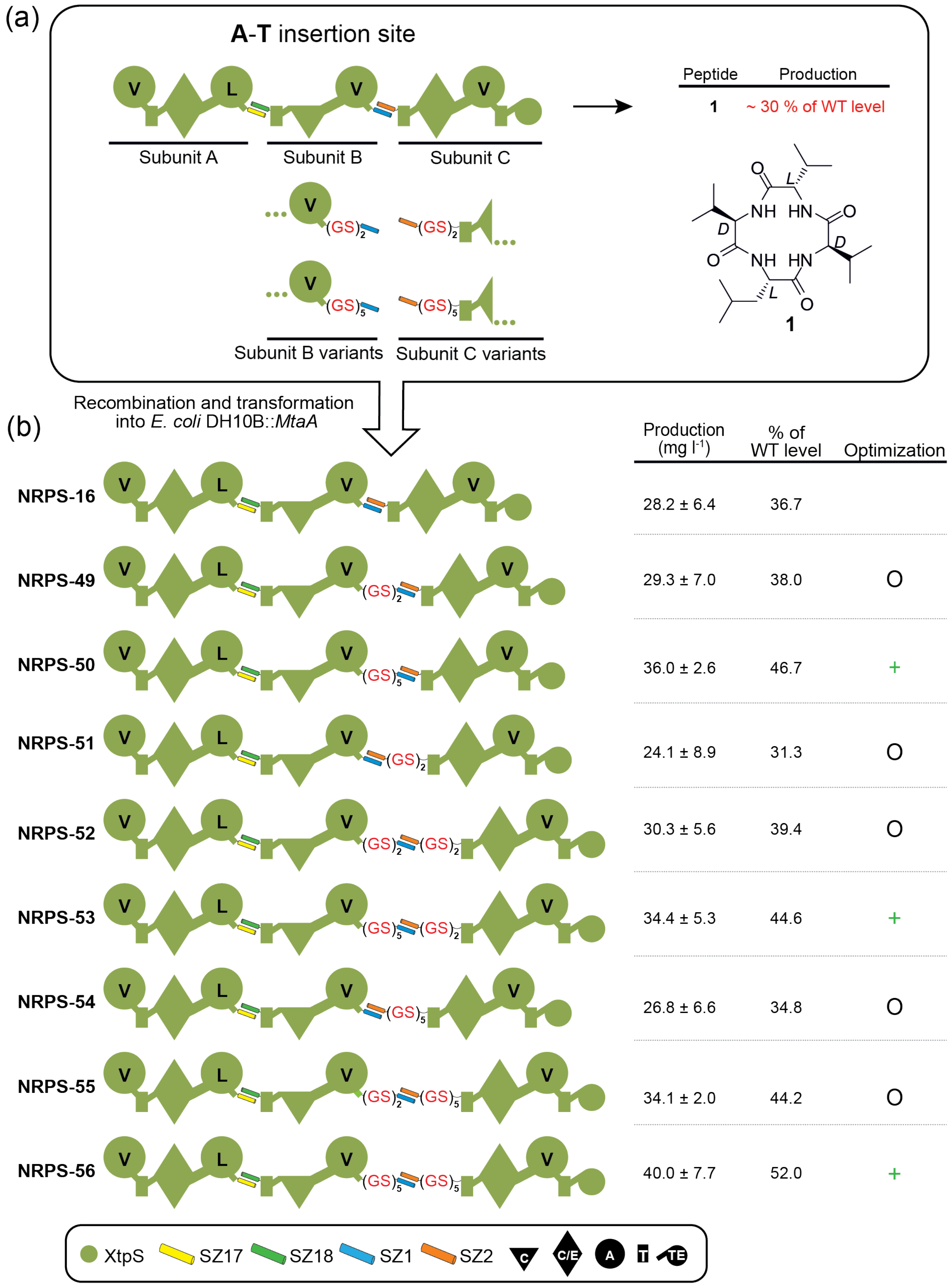

(a)
A-T insertion site
V
L
V
V
Subunit A
Subunit B
Subunit C
(GS)2
(GS)5
Subunit B variants
Subunit C variants
Peptide
Production
1
~ 30 % of WT level
O
L
NH
HN
O
D
D
O
NH
HN
L
O
1
Recombination and transformation into E. coli DH10B::MtaA
(b)
Production (mg l-1)
% of WT level
Optimization
NRPS-16
28.2 ± 6.4
36.7
NRPS-49
29.3 ± 7.0
38.0
O
NRPS-50
36.0 ± 2.6
46.7
+
NRPS-51
24.1 ± 8.9
31.3
O
NRPS-52
30.3 ± 5.6
39.4
O
NRPS-53
34.4 ± 5.3
44.6
+
NRPS-54
26.8 ± 6.6
34.8
O
NRPS-55
34.1 ± 2.0
44.2
O
NRPS-56
40.0 ± 7.7
52.0
+
XtpS
SZ17
SZ18
SZ1
SZ2
C
C/E
A
T
TE

242

Figure S25. More GS-optimized chimeric tri-partite XtpS NRPSs split at the A-T position. (a) Between each experimental approach, the production of non-optimized NRPS-16 varies, but is on average at ~30% of WT level. A set of modified subunit B and C variants were constructed by inserting GS stretches of varying length (4 AAs or 10 AAs) between subunit 2 and SZ1 and subunit 3 and SZ2. (b) Generated modified subunits were re-combined with non-modified subunits and transformed into *E. coli* DH10B::*MtaA* to obtain NRPS-49 to -56. Production titres of NRPS-49 to -56 were compared with each other and rated with from –, – –, – – – to O, +, ++, +++. Corresponding peptide yields (mg/L) and standard deviations are obtained from biological triplicate experiments. For domain assignment, the following symbols are used: (A, large circles), (T, rectangle), (C, triangle), (C/E, diamond), (TE, small circle); substrate specificities are assigned for all A domains and indicated by capital letters.

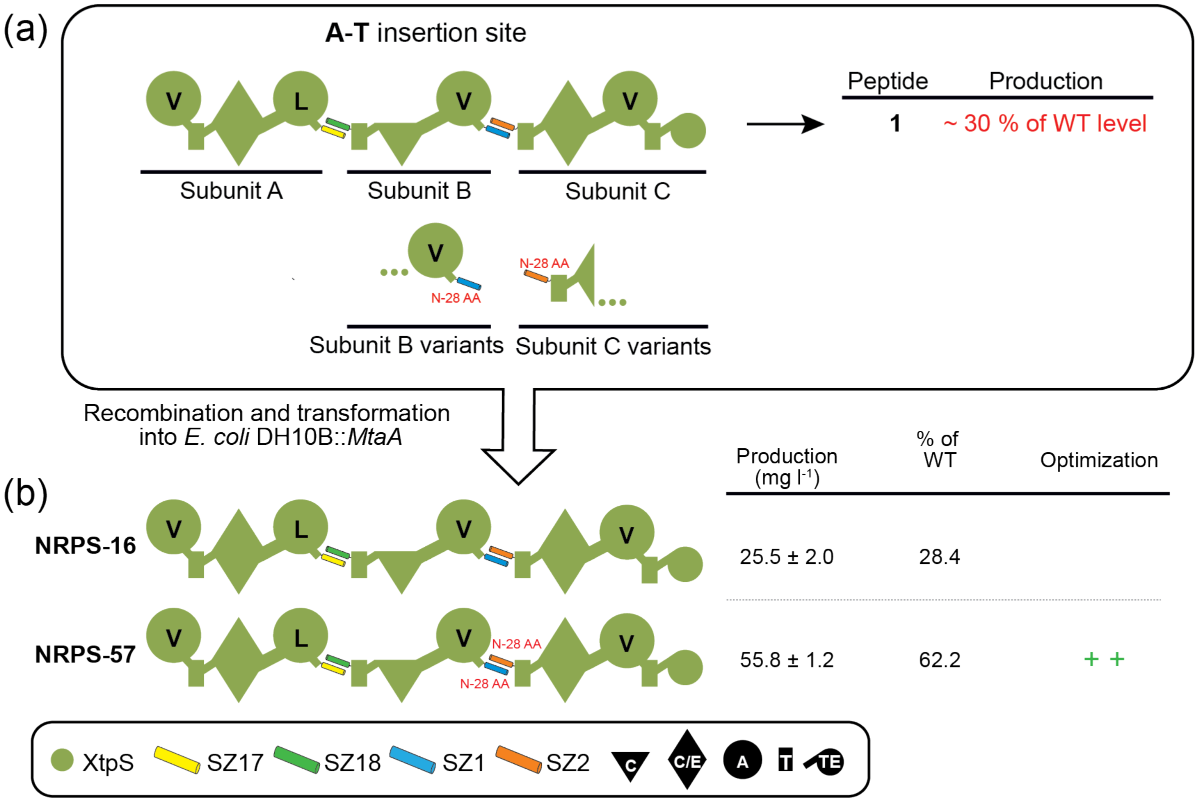

Figure S26. SZ1:2 truncation of chimeric di-partite XtpS NRPSs split at the A-T position. (a) Between each experimental approach, the production of non-optimized NRPS-16 varies, but is on average at ~30% of WT level. Subunit B and C variants were N-terminally truncated by 28 AA, respectively. (b) Generated modified subunits were re-combined with non-modified subunits and transformed into *E. coli* DH10B::*MtaA* to obtain NRPS-16 and -57.Corresponding peptide yields (mg/L) and standard deviations are obtained from biological triplicate experiments. Rating of production titres and domain assignment is as described before.

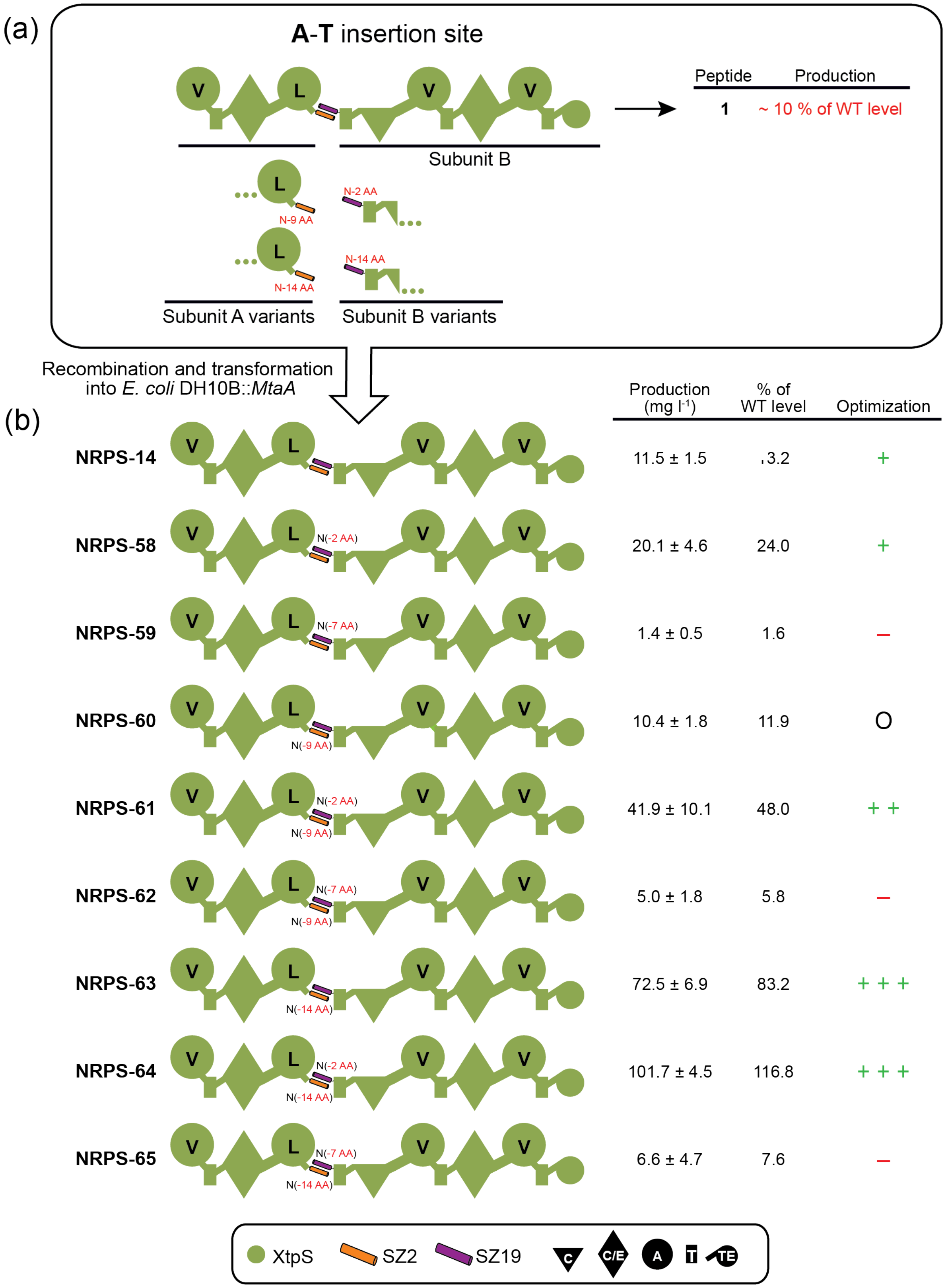
(a)
A-T insertion site
V
L
V
V
Subunit B
Peptide
Production
1
~ 10 % of WT level
N-9 AA
N-2 AA
N-14 AA
N-14 AA
Subunit A variants
Subunit B variants
Recombination and transformation into E. coli DH10B::MtaA
(b)
Production (mg l-1)
% of WT level
Optimization
NRPS-14 11.5 ± 1.5 13.2 +
NRPS-58 N(-2 AA) 20.1 ± 4.6 24.0 +
NRPS-59 N(-7 AA) 1.4 ± 0.5 1.6 –
NRPS-60 N(-9 AA) 10.4 ± 1.8 11.9 O
NRPS-61 N(-2 AA) N(-9 AA) 41.9 ± 10.1 48.0 + +
NRPS-62 N(-7 AA) N(-9 AA) 5.0 ± 1.8 5.8 –
NRPS-63 N(-14 AA) 72.5 ± 6.9 83.2 + + +
NRPS-64 N(-2 AA) N(-14 AA) 101.7 ± 4.5 116.8 + + +
NRPS-65 N(-7 AA) N(-14 AA) 6.6 ± 4.7 7.6 –
XtpS
SZ2
SZ19
C
C/E
A
T
TE

Figure S27. SZ2:19 truncation of chimeric di-partite XtpS NRPSs split at the A-T position. (a) Between each experimental approach, the production of non-optimized NRPS-14 varies, but is on average at ~10% of WT level. A set of modified subunit A and B variants were constructed by N-terminally truncating SZ2 by 9 AAs and 14 AAs and SZ19 by 2 AAs and 7 AAs, respectively. (b) Generated modified subunits were re-combined with non-modified subunits and transformed into E. coli DH10B::MtaA to obtain NRPS-14, -59 to -66. Corresponding peptide yields (mg/L) and standard deviations are obtained from biological triplicate experiments. Rating of production titres and domain assignment is as described before.

(a)

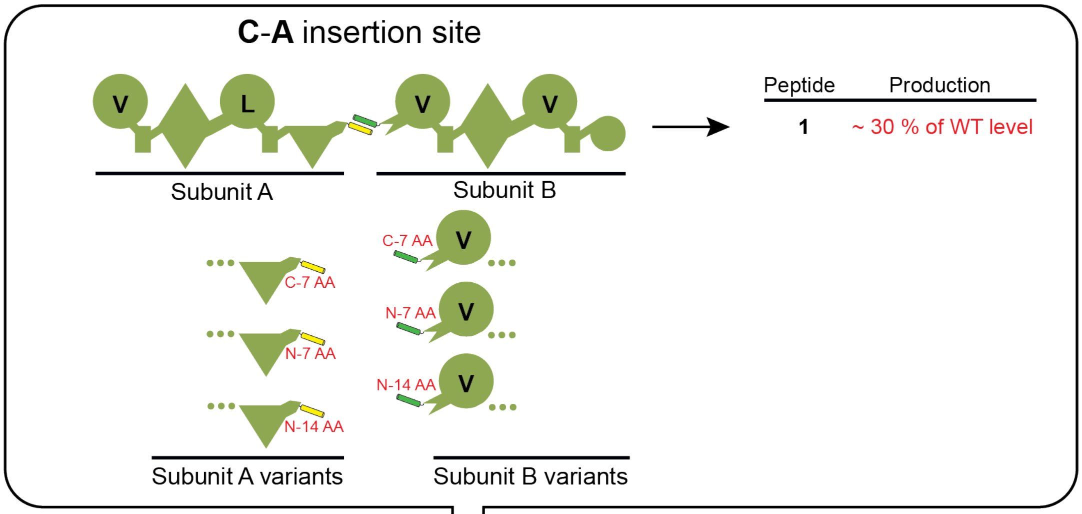

Recombination and transformation into *E. coli* DH10B::*MtaA*

(b)

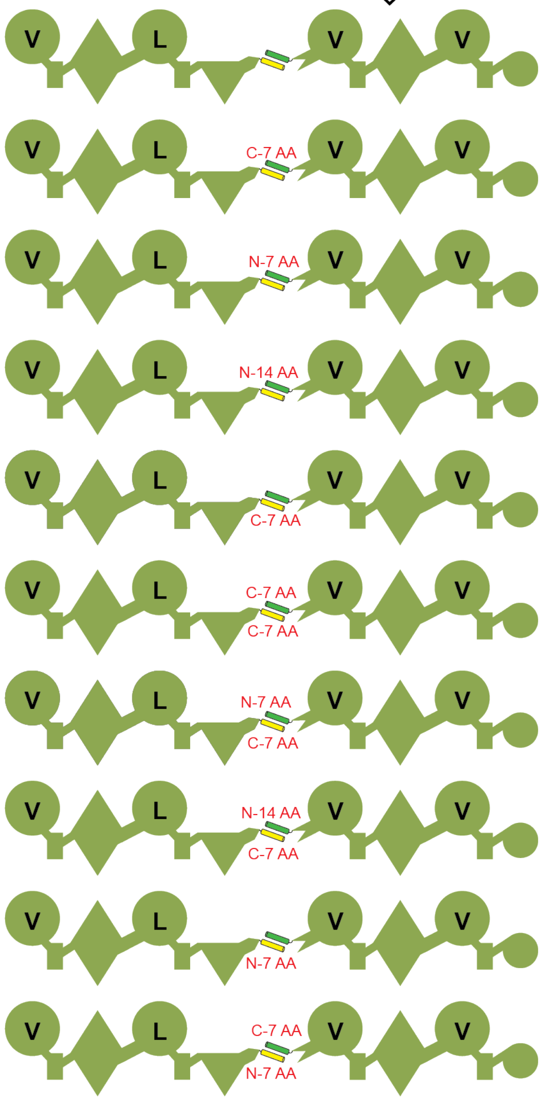

	Production (mg l^{-1})	% of WT level	Optimization
**NRPS-1**	24.7 ± 0.9	27.6	
**NRPS-66**	24.5 ± 2.5	27.3	O
**NRPS-67**	16.9 ± 1.9	18.8	–
**NRPS-68**	11.7 ± 0.8	13.0	– –
**NRPS-69**	24.7 ± 1.5	27.5	O
**NRPS-70**	28.0 ± 1.9	31.1	O
**NRPS-71**	19.9 ± 1.9	22.1	–
**NRPS-72**	7.5 ± 1.1	8.3	– –
**NRPS-73**	9.3 ± 0.3	10.4	– –
**NRPS-74**	14.6 ± 1.9	16.2	–

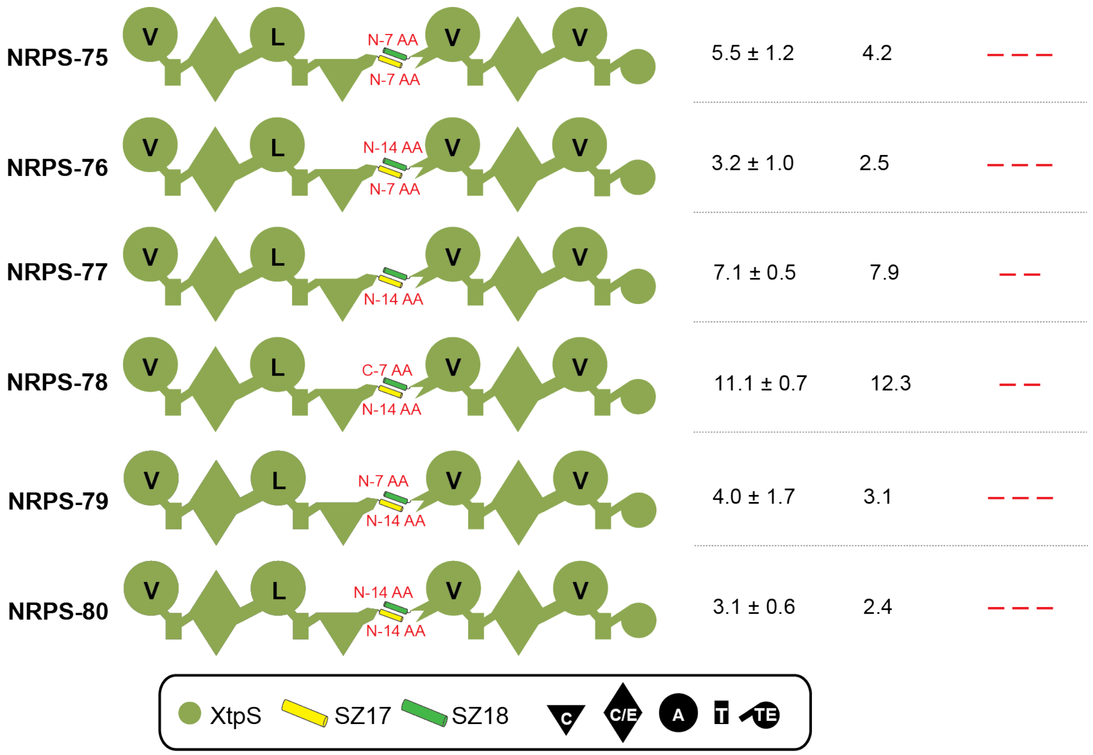

Figure S28. SZ17:18 truncation of chimeric di-partite XtpS NRPSs split at the C-A position. (a) Between each experimental approach, the production of non-optimized NRPS-1 varies, but is on average at ~30% of WT level. A set of modified subunit A and B variants were constructed by N- terminally truncating SZ17 and SZ18 by 7 AAs and 14 AAs, respectively and C-terminally truncating SZ17 and SZ18 by 7 AAs. (b) Generated modified subunits were re-combined with non-modified subunits and transformed into E. coli DH10B::MtaA to obtain NRPS-1, -67 to -81. Corresponding peptide yields (mg/L) and standard deviations are obtained from biological triplicate experiments. Rating of production titres and domain assignment is as described before.

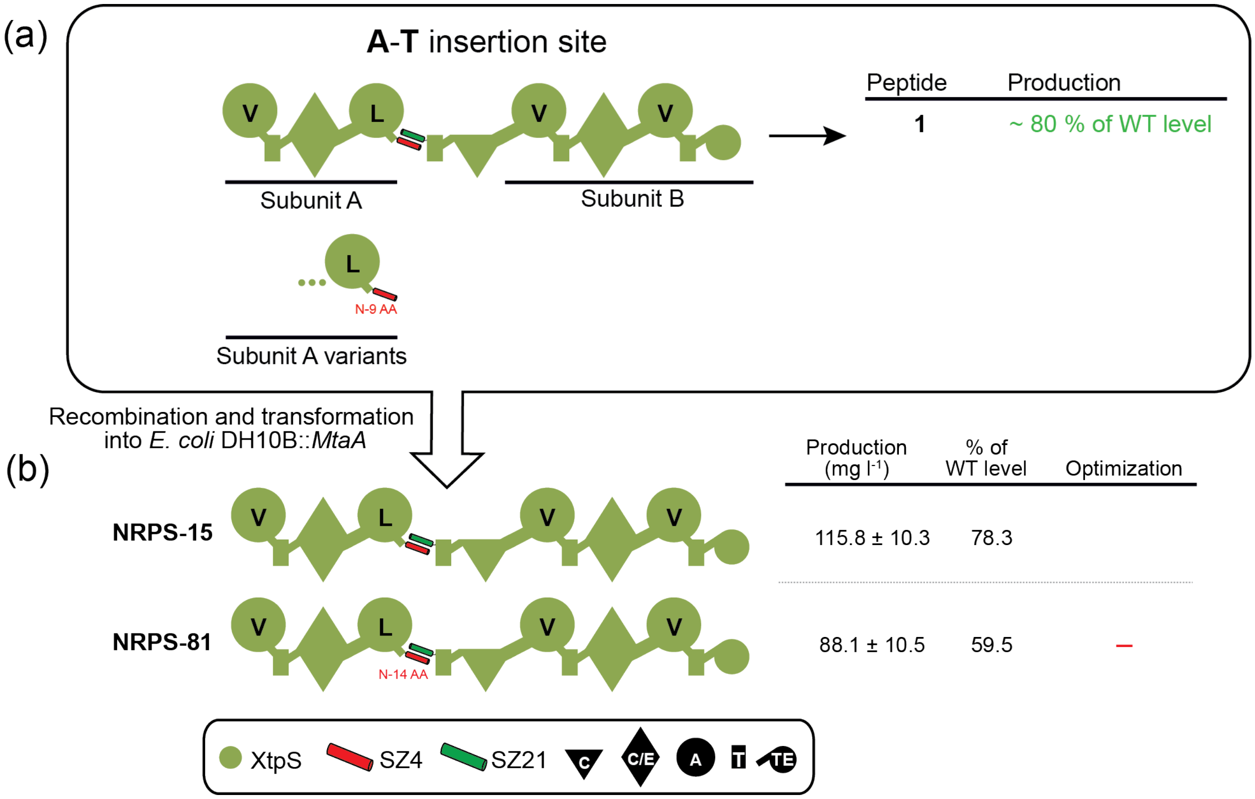

Figure S29. SZ4:21 truncation of chimeric di-partite XtpS NRPSs split at the A-T position. (a) Between each experimental approach, the production of non-optimized NRPS-15 varies, but is on average at ~30% of WT level. Modified subunit A was constructed by N- terminally truncating SZ4 by 14 AAs. (b) Generated modified subunits were re-combined with non-modified subunits and transformed into E. coli DH10B::MtaA to obtain NRPS-1, -67 to -81. Corresponding peptide yields (mg/L) and standard deviations are obtained from biological triplicate experiments. Rating of production titres and domain assignment is as described before.

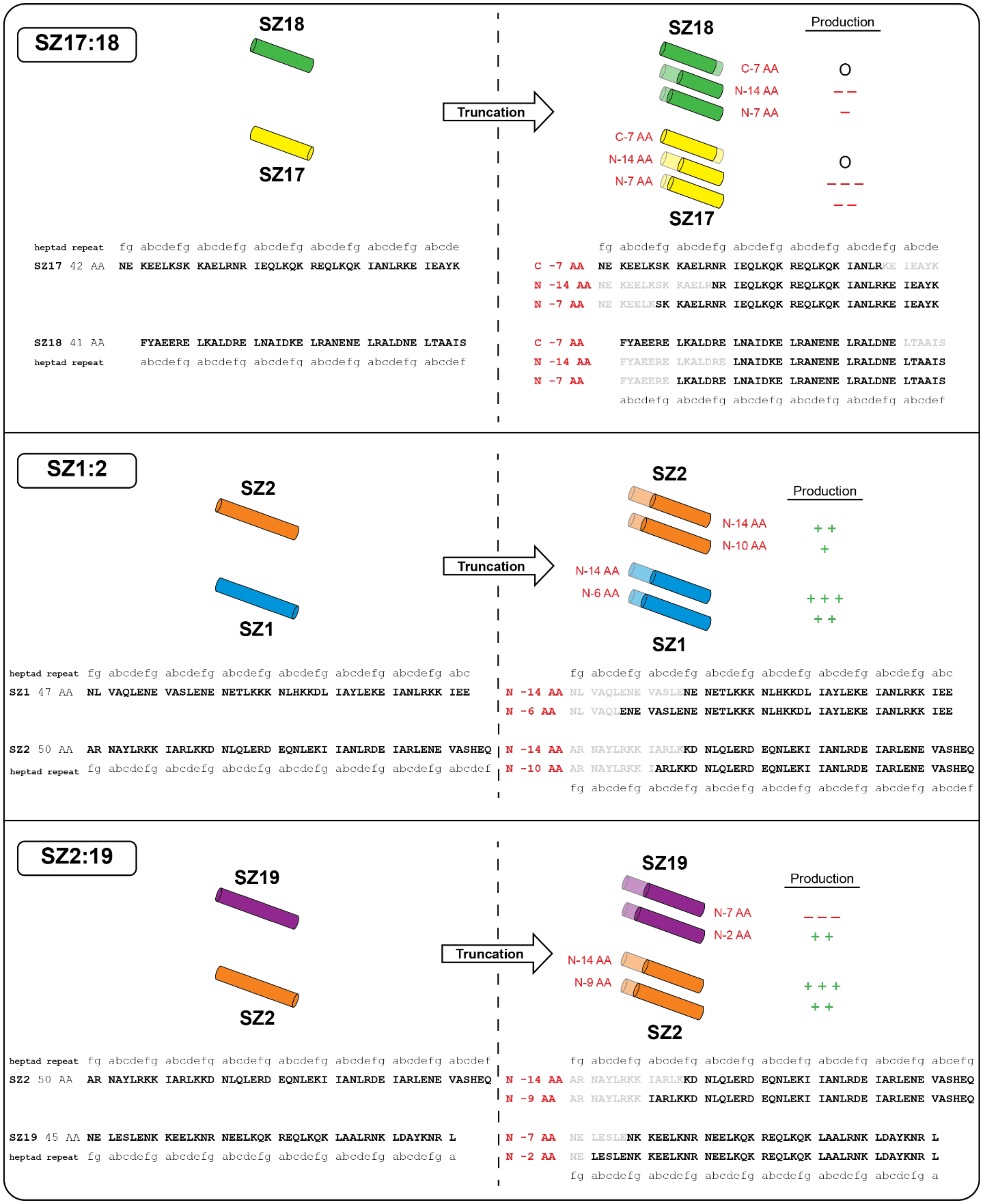
SZ17:18
SZ18
SZ17
Truncation
Production
C-7 AA
N-14 AA
N-7 AA
O
heptad repeat fg abcdefg abcdefg abcdefg abcdefg abcdefg abcde
SZ17 42 AA NE KEELKSK KAELRNR IEQLKQK REQLKQK IANLRKE IEAYK
C -7 AA NE KEELKSK KAELRNR IEQLKQK REQLKQK IANLRKE IEAYK
N -14 AA NE KEELKSK KAELRNR IEQLKQK REQLKQK IANLRKE IEAYK
N -7 AA NE KEELKSK KAELRNR IEQLKQK REQLKQK IANLRKE IEAYK
SZ18 41 AA FYAEERE LKALDRE LNAIDKE LRANENE LRALDNE LTAAIS
heptad repeat abcdefg abcdefg abcdefg abcdefg abcdefg abcdef
C -7 AA FYAEERE LKALDRE LNAIDKE LRANENE LRALDNE LTAAIS
N -14 AA FYAEERE LKALDRE LNAIDKE LRANENE LRALDNE LTAAIS
N -7 AA FYAEERE LKALDRE LNAIDKE LRANENE LRALDNE LTAAIS
SZ1:2
SZ2
SZ1
N-14 AA
N-10 AA
N-6 AA
+ +
+
+ + +
heptad repeat fg abcdefg abcdefg abcdefg abcdefg abcdefg abcdefg abc
SZ1 47 AA NL VAQLENE VASLENE NETLKKK NLHKKDL IAYLEKE IANLRKK IEE
N -14 AA NL VAQLENE VASLENE NETLKKK NLHKKDL IAYLEKE IANLRKK IEE
N -6 AA NL VAQLENE VASLENE NETLKKK NLHKKDL IAYLEKE IANLRKK IEE
SZ2 50 AA AR NAYLRKK IARLKKD NLQLERD EQNLEKI IANLRDE IARLENE VASHEQ
heptad repeat fg abcdefg abcdefg abcdefg abcdefg abcdefg abcdefg abcdef
N -14 AA AR NAYLRKK IARLKKD NLQLERD EQNLEKI IANLRDE IARLENE VASHEQ
N -10 AA AR NAYLRKK IARLKKD NLQLERD EQNLEKI IANLRDE IARLENE VASHEQ
SZ2:19
SZ19
N-7 AA
N-2 AA
N-9 AA
heptad repeat fg abcdefg abcdefg abcdefg abcdefg abcdefg abcdefg abcdef
SZ2 50 AA AR NAYLRKK IARLKKD NLQLERD EQNLEKI IANLRDE IARLENE VASHEQ
fg abcdefg abcdefg abcdefg abcdefg abcdefg abcdefg abcefg
N -14 AA AR NAYLRKK IARLKKD NLQLERD EQNLEKI IANLRDE IARLENE VASHEQ
N -9 AA AR NAYLRKK IARLKKD NLQLERD EQNLEKI IANLRDE IARLENE VASHEQ
SZ19 45 AA NE LESLENK KEELKNR NEELKQK REQLKQK LAALRNK LDAYKNR L
heptad repeat fg abcdefg abcdefg abcdefg abcdefg abcdefg abcdefg a
N -7 AA NE LESLENK KEELKNR NEELKQK REQLKQK LAALRNK LDAYKNR L
N -2 AA NE LESLENK KEELKNR NEELKQK REQLKQK LAALRNK LDAYKNR L

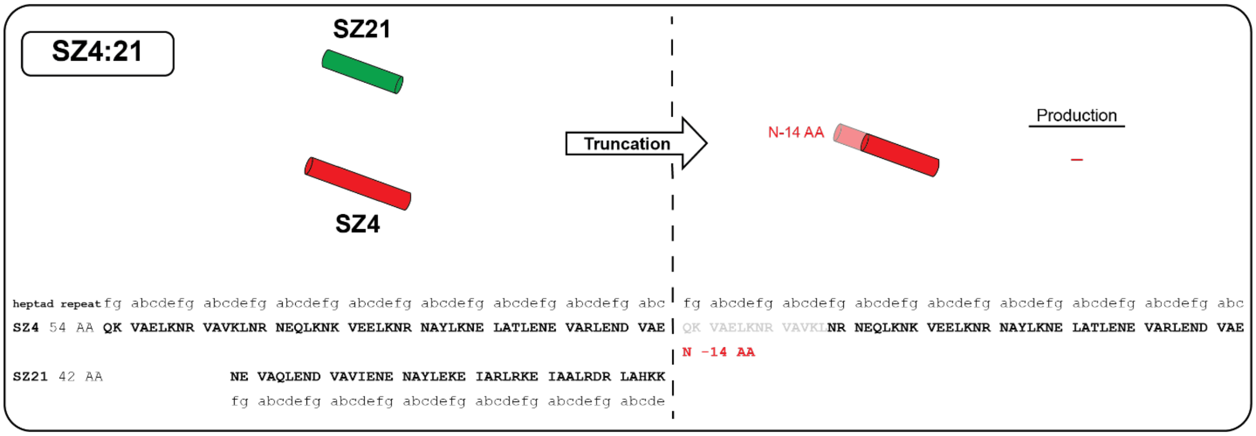

Figure S30. Overview of truncated SYNZIPs. Left row: schematic representation of SYNZIPs and their corresponding AAs sequences. Right row. Truncated SYNZIPs variants and corresponding AAs sequence. Impact of truncated SYNZIPs on type S NRPSs is indicated and rated from –, – –, – – – to O, +, ++, +++.

3. References

1. Kegler, C.; Nollmann, F. I.; Ahrendt, T.; Fleischhacker, F.; Bode, E.; Bode, H. B., Rapid determination of the amino acid configuration of xenotetrapeptide. *Chembiochem* **2014,** *15* (6), 826-8.
2. Abbood, N.; Duy Vo, T.; Watzel, J.; Bozhueyuek, K. A. J.; Bode, H. B., Type S Non-Ribosomal Peptide Synthetases for the Rapid Generation of Tailormade Peptide Libraries. *Chemistry* **2022,** *28* (26), e202103963.
3. Hanahan, D., Studies on transformation of Escherichia coli with plasmids. *J Mol Biol* **1983,** *166* (4), 557-80.
4. Schimming, O.; Fleischhacker, F.; Nollmann, F. I.; Bode, H. B., Yeast homologous recombination cloning leading to the novel peptides ambactin and xenolindicin. *Chembiochem* **2014,** *15* (9), 1290-4.
5. Bozhueyuek, K. A. J.; Watzel, J.; Abbood, N.; Bode, H. B., Synthetic Zippers as an Enabling Tool for Engineering of Non-Ribosomal Peptide Synthetases*. *Angew Chem Int Ed Engl* **2021,** *60* (32), 17531-17538.
6. Shao, Z.; Zhao, H.; Zhao, H., DNA assembler, an in vivo genetic method for rapid construction of biochemical pathways. *Nucleic Acids Res* **2009,** *37* (2), e16.
7. Yin, J.; Hoffmann, M.; Bian, X.; Tu, Q.; Yan, F.; Xia, L.; Ding, X.; Stewart, A. F.; Müller, R.; Fu, J.; Zhang, Y., Direct cloning and heterologous expression of the salinomycin biosynthetic gene cluster from Streptomyces albus DSM41398 in Streptomyces coelicolor A3(2). *Sci Rep* **2015,** *5*, 15081.
8. Wang, H.; Li, Z.; Jia, R.; Yin, J.; Li, A.; Xia, L.; Yin, Y.; Müller, R.; Fu, J.; Stewart, A. F.; Zhang, Y., ExoCET: exonuclease in vitro assembly combined with RecET recombination for highly efficient direct DNA cloning from complex genomes. *Nucleic Acids Res* **2018,** *46* (5), e28.
9. Gibson, D. G.; Young, L.; Chuang, R. Y.; Venter, J. C.; Hutchison, C. A., 3rd; Smith, H. O., Enzymatic assembly of DNA molecules up to several hundred kilobases. *Nat Methods* **2009,** *6* (5), 343-5.
10. Fu, C.; Donovan, W. P.; Shikapwashya-Hasser, O.; Ye, X.; Cole, R. H., Hot Fusion: an efficient method to clone multiple DNA fragments as well as inverted repeats without ligase. *PLoS One* **2014,** *9* (12), e115318.
